# Toxicity and inhibition mechanism of gallic acid on physiology and fermentation performance of *Escherichia coli*

**DOI:** 10.1186/s40643-022-00564-w

**Published:** 2022-07-23

**Authors:** Lina Liu, Xiaolong Ma, Muhammad Bilal, Linlin Wei, Shijie Tang, Hongzhen Luo, Yuping Zhao, Zhaoyu Wang, Xuguo Duan

**Affiliations:** 1 School of Life Science and Food Engineering, Huaiyin Institute of Technology, Huaian, 223003 China; 2 College of Light Industry and Food Engineering, Nanjing Forestry University, Nanjing, 210037 Jiangsu China

**Keywords:** *Escherichia coli*, Gallic acid, Physiological mechanism, Fermentation characteristics, Transcriptional analysis

## Abstract

Gallic acid is a natural phenolic acid that has a stress inhibition effect on *Escherichia coli*. This study by integrates fermentation characteristics and transcriptional analyses to elucidate the physiological mechanism of *E. coli* 3110 response to gallic acid. Compared with the control (without stress), the cell growth was severely retarded, and irregular cell morphology appeared in the case of high levels of gallic acid stress. The glucose consumption of *E. coli* was reduced successively with the increase of gallic acid content in the fermentation medium. After 20 h of gallic acid stress, cofactor levels (ATP, NAD^+^ and NADH) of *E. coli* 3110 were similarly decreased, indicating a more potent inhibitory effect of gallic acid on *E. coli*. The transcriptional analysis revealed that gallic acid altered the gene expression profiles related to five notable differentially regulated pathways. The genes related to the two-component system were up-regulated, while the genes associated with ABC-transporter, energy metabolism, carbon metabolism, and fatty acid biosynthesis were down-regulated. This is the first report to comprehensively assess the toxicity of gallic acid on *E. coli*. This study has implications for the efficient production of phenolic compounds by *E. coli* and provides new ideas for the study of microbial tolerance to environmental stress and the identification of associated tolerance targets.

**Graphical abstract:**

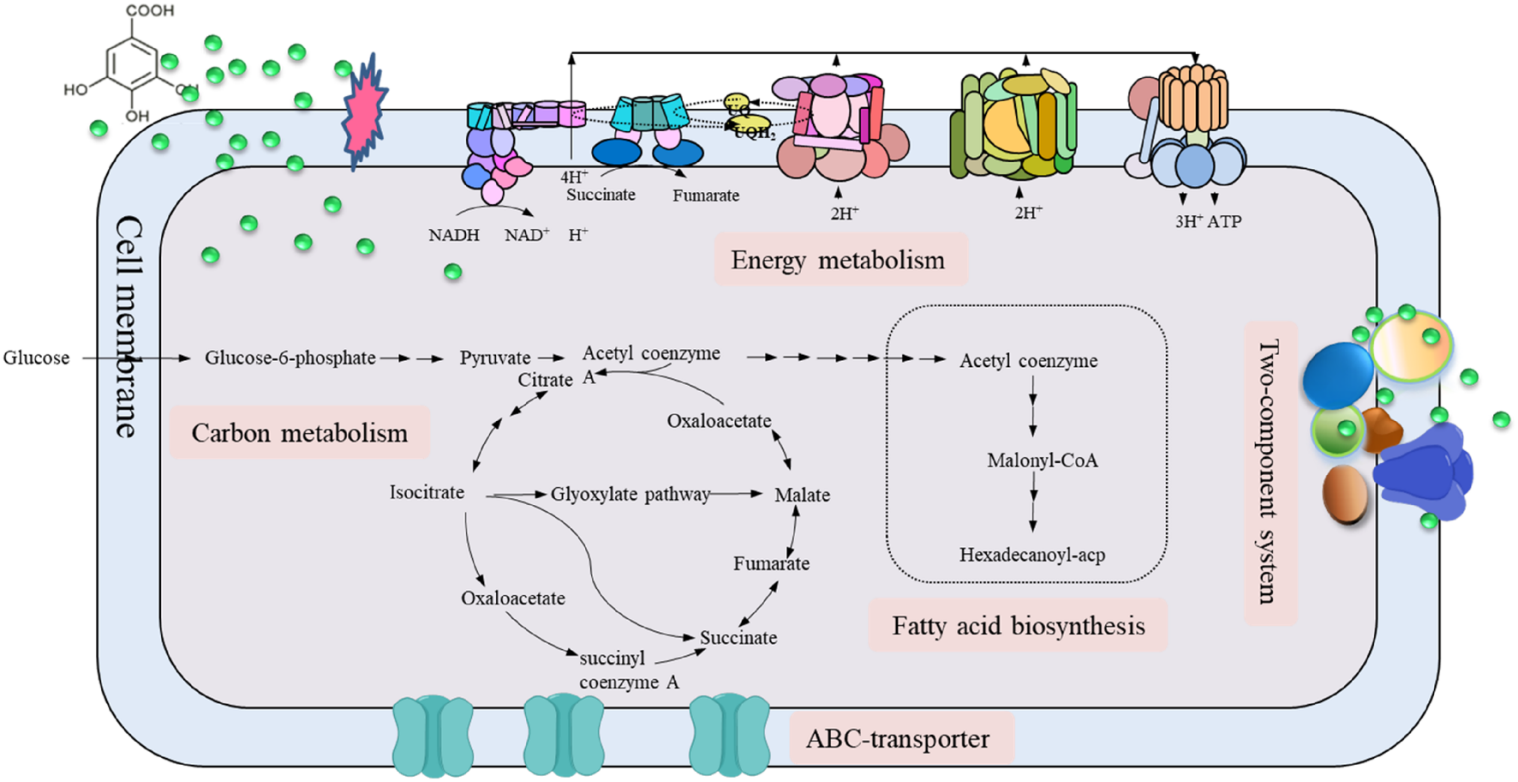

**Supplementary Information:**

The online version contains supplementary material available at 10.1186/s40643-022-00564-w.

## Introduction

Gallic acid is a natural phenolic acid with many significant biological properties, such as antioxidant, antibacterial, anti-ageing, etc. Therefore, it is widely used in producing animal and vegetable oils, milk powder, fish products, bread, and other food products as an additive (Limpisophon and Schleining 2016; Wang et al. 2017, 2016). The gallic acid content is low in nature, mainly found in blueberries, walnuts, apples, and other plants, which significantly limits its large-scale commercial production. Due to the scarcity of raw materials, unfavorable reaction conditions, and environmental contamination, the enzymatic conversion method—the primary industrial way of producing gallic acid—is also significantly constrained in the process of producing gallic acid. (Rewatkar et al. 2016). As a green and efficient method for producing biological products, microbial fermentation has been widely employed in the industrial production of natural products due to its advantages, including cheap raw materials, mild reaction conditions, no pollutant emission and simple extraction (Liu et al. 2021). During the microbial fermentation process, the presence of phenolic compounds can significantly inhibit the microorganisms’ growth and the production of target metabolites. For example, phenolic acids are highly toxic to *Solventogenic clostridia* because of their low water solubility and hydrophobicity (Liu et al. 2018). In addition, the physiological metabolism of a typical robust strain *Saccharomyces cerevisiae* DQ1 can be inhibited by phenolic compounds during ethanol fermentation (Gu et al. 2015). Phenolic acids also have an inhibitory effect on foodborne microorganisms, such as *Staphylococcus aureus*, which can inhibit the growth in broth and meat broth (Oliveira et al. 2010; Tassou and Nychas 1994).

**Fig. 1:**
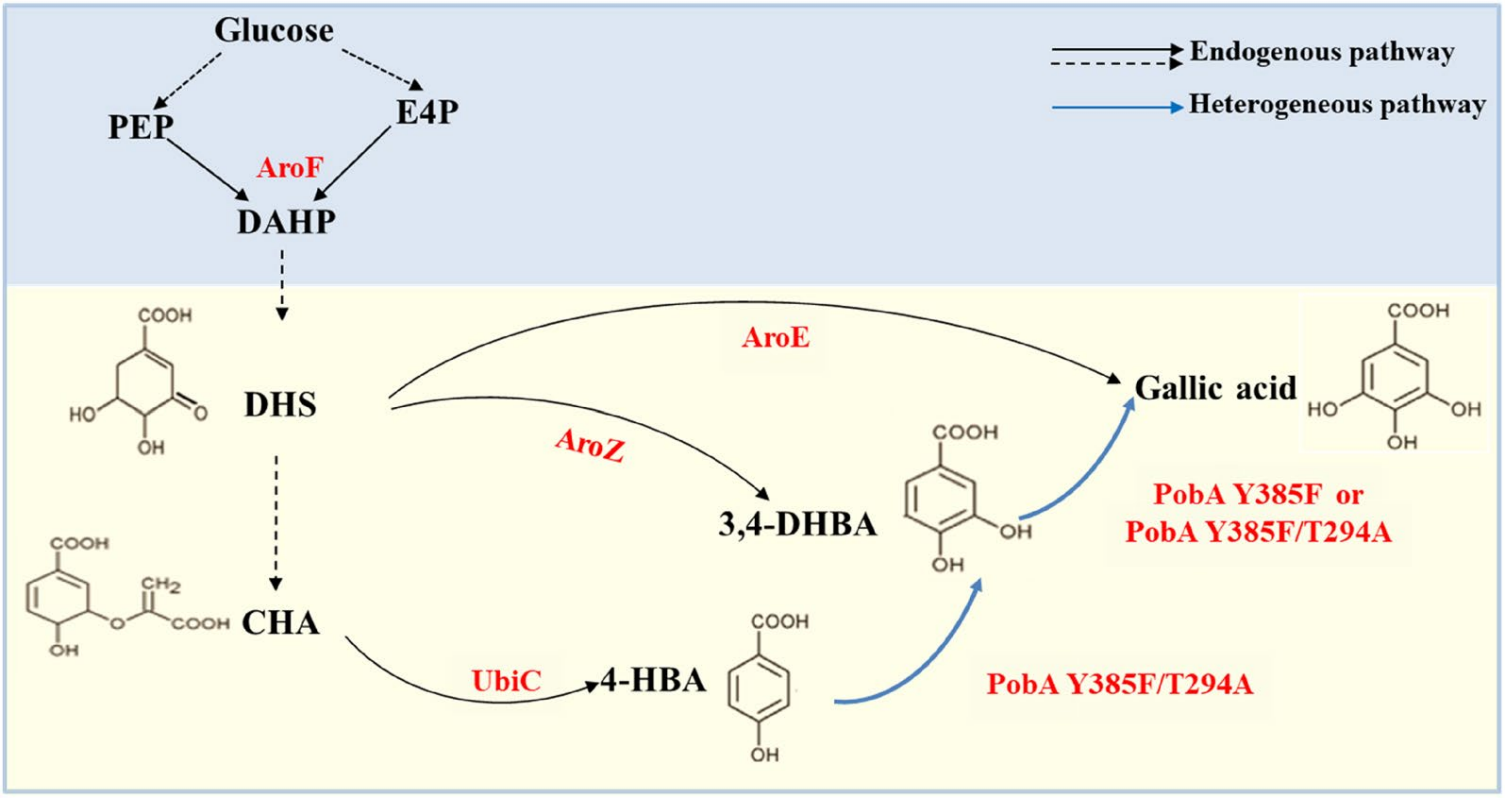
Gallic acid endogenous pathway and heterogeneous pathway of *E. coli.* *E4P* erythrose 4-phosphate, *PEP* phosphoenolpyruvate, *DAHP* 3-deoxy-d-arobino-heptulosonate 7-phosphate, *DHS* 3-dehydroshikimate, *CHA* chorismate, *4-HBA* 4-hydroxybenzoic acid, *3,4-DHBA* protocatechuic acid, *AroF* 3-deoxy-7-phosphoheptulonate synthase, *AroZ* 3-dehydroshikimate dehydratase, *AroE* shikimate dehydrogenase, *UbiC* chorismate lyase

*Escherichia coli* is an ideal strain for producing target products by microbial fermentation in the industry. Due to its advantages, including easy culture conditions, rapid growth, metabolic plasticity, and the abundance of tools for genetic and genomic engineering (Pontrelli et al. 2018), *E. coli* is superior to yeast, a commonly used eukaryotic microbe for industrial production, in the biosynthesis of some natural products. With the rapid rise and development of genetic engineering technology and metabolic engineering breeding strategies, a large number of genetically engineered strains of *E. coli* have recently been constructed, significantly improving the yield and production efficiency of the target product (Fang et al. 2021; Guo et al. 2020; Liu et al. 2016b; Wang et al. 2021). In the gallic acid endogenous pathway of *E. coli*, 3-deoxy-d-arobino-heptulosonate 7-phosphate (DAHP) is formed by condensation of Phosphoenolpyruvate (PEP) from glycolysis and erythrose 4-phosphate (E4P) from pentose phosphate by DAHP synthetase (AroF). Then DAHP generates 3-dehydroshikimate (DHS) through the shikimic acid pathway. Finally, shikimic acid dehydrogenase (AroE) catalyzes the formation of gallic acid from DHS (Fig. 1). Still, this catalytic process is so inefficient that the presence of gallic acid can hardly be detected (Kambourakis et al. 2000). Kambourakis et al. successfully constructed a gallic acid heterologous synthetic pathway by expressing 3-dehydroshikimate dehydratase (AroZ) and p-hydroxybenzoate hydroxylase mutant PobA Y385F/T294A from *Pseudomonas aeruginos* in *E. coli* KL7 (Fig. 1), which resulted in a yield of 20 g/L gallic acids. This is the highest yield of gallic acid reported in the literature, but the tolerance mechanism of *E. coli* KL7 to gallic acid needs further study. Recently, another heterologous synthetic pathway has been successfully constructed by expressing chorismate lyase (UbiC) and the mutant PobA Y385F/T294A (Fig. 1). With the construction of this pathway, the gallic acid was 1.3 g/L. Some progress has been made in gallic acid production by *E. coli*, but the yield is still low (Chen et al. 2017).

As a phenolic compound, gallic acid has a stress inhibition effect on *E. coli*, which can affect the integrity of the cell membrane and damage the hydrophobic structure after entering the cell, thus affecting the physiological metabolism of the cell (Mills et al. 2009) (Additional file 1: Fig. S1). This situation seriously limits the ability of *E. coli* to synthesize gallic acid. To realize the efficient synthesis of gallic acid by *E. coli*, the premise is to reduce or remove the stress inhibition of gallic acid in *E. coli*. However, the comprehensive physiological mechanism of *E. coli* response to phenolics remains unclear, and there are relatively few studies on this issue. Therefore, it is imperative to systematically study the stress inhibition mechanism of gallic acid in *E. coli. E. coli* K12 wild-type strain W3110 (F-, IN (rrnD-rrnE) 1,λ-) is a standard prototrophic wild-type strain and is well-characterized, which is a commonly used strain for the production of high value products, such as tryptophan (Liu et al. 2016a), methionine (Niu et al. 2020), homoserine (Li et al. 2016). To clarify the overall physiological mechanism of *E. coli* response to gallic acid, the transcriptional analysis of *E. coli* W3110 under gallic acid stress was carried out by RNA-seq. A comprehensive insight into the physiological mechanism of *E. coli* against gallic acid stress was obtained by the identification and functional classification of differentially expressed genes*.* It is instructive to realize the efficient production of gallic acid using *E. coli* via an engineering strategy for constructing more robust strains based on the analytical results from our study.

## Materials and methods

### Microorganism and cultivation conditions

*E. coli* strain W3110 was obtained from Novagen (Madison, USA). Seed cultures (500 µL) of W3110 were used to inoculate 100 mL portions of fermentation medium in a 500 mL flask in a rotary shaker (200 rpm). The flask fermentation medium contains (per litre) (pH 7.2): 15 g K_2_HPO_4_, 10 g glucose, 2 g citric acid, 2 g yeast, 1.6 g (NH_4_)_2_SO_4_, 1 g MgSO_4_·7H_2_O, 0.075 g FeSO_4_·7H_2_O, and 0.0129 g CaCl_2_. The flask fermentation was performed for 28 h with pH maintained at 6.5–7.4 by 20% NH_4_OH to the medium every 8 h and temperature kept at 37 °C. When the initial glucose in the medium was used up, 10 g/L glucose was added to the medium every 8 h. To investigate the fermentation performance of *E. coli* under gallic acid stress, a certain amount of gallic acid was added to the fermentation medium. During the flask fermentation, samples were taken every 4–5 h, and all operations were repeated three times. Fermentation parameters are shown as the mean ± standard deviation of these three replicates.

### Analytical methods

Cell concentration was measured using the optical density at 600 nm (OD_600_). For detecting the concentration of glucose, the supernatant was obtained after centrifugation of the fermentation broth, and then glucose concentration and acetate level were determined using high-performance liquid chromatography (HPLC) at 50 °C with an Aminex HPX-87H column (300 mm × 7.8 mm; Bio-Rid, Hercules, CA), 50 mM H_2_SO_4_ as the mobile phase with a flow rate of 0.5 mL/min. Test kits purchased from Nanjing Jiancheng Bioengineering Institute were used for determining the intracellular levels of ATP, NAD^+^ and NADH, respectively (Jiang et al. 2020). In calculating ATP content, intracellular total protein concentration was determined using the Bradford method according to the reported method (Bradford 1976).

### Scanning electron microscopy (SEM) image of *E. coli* W3110

Biomass samples from the fermentation broth were obtained after fermentation for 20 h, centrifuged at 12,000 × g for 10 min, and then washed twice with 2.5% glutaraldehyde solution. The cell morphology of *E. coli* W3110 was observed by SEM (JEOL, JSM-840A) according to the method in the literature (Falero et al. 2020).

### RNA extraction and transcriptome sequencing

Biomass samples from the fermentation broth were obtained after fermentation for 20 h, centrifuged at 12,000 × g for 10 min, and then washed twice with phosphate-buffered saline buffer (pH 7.4). *E. coli* W3110 cells were immediately quenched in liquid nitrogen for 2 min, and then the total RNA of each biomass sample was extracted by the Trizol reagent. A Bioanalyzer (Agilent 2100, CA) was employed to evaluate the RNA quality, and the quantity and concentration of the extracted RNA were determined by Nanodrop 2000 (Thermo Scientific, MA). The RNA library and sequencing were constructed by Novogene Bioinformatics Technology Co., Ltd. (Tianjin, China), employing Illumina HiSeq X Ten. Raw transcriptome sequencing data for all samples can be downloaded from the National Center for Biotechnology Information with the access number PRJNA783627 (website: https://dataview.ncbi.nlm.nih.gov/object/PRJNA783627). The differential expression genes (DEGs) were identified by the threshold (fold change > 1.5) and statistically significant differences adjusted *p* value (padj) < 0.05. Gene annotation was carried out by adopting the open data from the Candida Genome Database (CGD). The Kyoto Encyclopedia of Genes and Genomes (KEGG) database was used to analyse the gene function. Z score is used to clearly and intuitively display the transcriptional expression profile of different genes in the heatmap. Z score is calculated as follows, x: the FPKM values of differential expression genes; µ: the average of FPKM; δ: the total standard deviation of FPKM.

$$ {\text{Z score}} = \left( {{\text{x}} - \mu } \right)/\delta $$

## Results

### Impact of gallic acid on fermentation characteristics of *E. coli* W3110

**Fig. 2:**
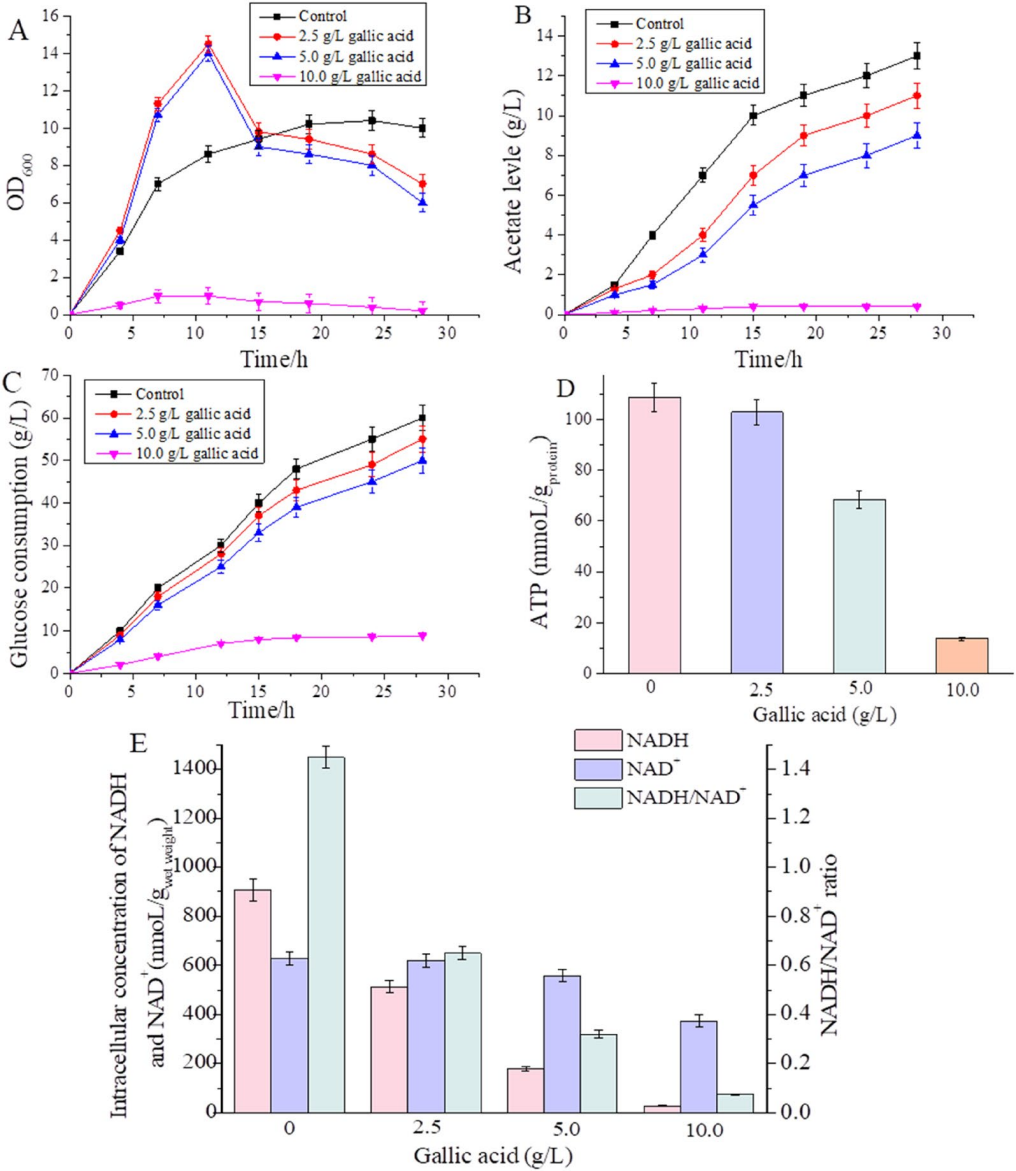
Shaking flask fermentation of *E. coli* under different concentrations of gallic acid stress. **A** Biomass; **B** acetate level; **C** glucose consumption; **D** ATP content; **E** intracellular concentration of NADH, NAD^+^ at 20 h, and the ratio of NADH/ NAD^+^

To investigate the effects of gallic acid on macro fermentation parameters of *E. coli,* W3110 was cultured by flask fermentation under different gallic acid concentrations. As shown in Fig. 2, compared to control (without stress), cell biomass increased at the early stage of fermentation but decreased significantly with the extension of fermentation time under low concentration gallic acid (< = 5.0 g/L) stress, whereas the growth of *E. coli* was severely inhibited under high concentration of gallic acid (10.0 g/L) stress. When the gallic acid concentration was low (< = 5.0 g/L), the biomass of *E. coli* was higher than that of the control during the first 10 h of fermentation processes (Fig. 2A). It has been reported that aromatic acids in *E. coli* could be catabolized, and then enter the TCA (Tricarboxylic acid) cycle, which can be used as a carbon source for the growth and metabolism of *E. coli* (DíAz et al. 2001)*.* Therefore, during the early stages of growth, when the inhibition effect is not significant, low concentration of gallic acid may be advantageous to *E. coli* growth as carbon sources. With the increase of gallic acid content in the fermentation medium, the glucose consumption of *E. coli* decreased successively (Fig. 2C). Acetate is a common by-product of *E.coli* fermentation (Liu et al. 2016a); compared to control, the overall acetate levels decreased with the rise of gallic acid concentration during the entire fermentation process (Fig. 2B). The carbon source for acetate synthesis comes from glucose metabolites (Liu et al. 2016b); thus, the decreased glucose consumption may lead to lowered acetate synthesis.

In addition to the macro fermentation parameters, the levels of micro fermentation parameters (NADH, NAD^+^ and ATP) of *E. coli* W3110 were also measured under gallic acid stress. Metabolic cofactors such as NADH, NAD^+^ and ATP play essential roles in a large number of cellular reactions. The content of NADH, NAD^+^, NADH/NAD^+^ and ATP in cells was detected. The highest intracellular levels of NADH (908 nmoL/g_wet weight_), NAD^+^ (629 nmoL/g_wet weight_) and ATP (108 mmoL/g_protien_) were simultaneously achieved at 20 h of fermentation without gallic acid stress; whereas, as the gallic acid concentration increased, those three data was decreased (Fig. 2D, E). Under 2.5 g/L, 5 g/L and 10 g/L gallic acid stress, the ratio of NADH/NAD^+^ was, respectively, 57.1%, 21.4% and 5.4% of the control (Fig. 2E). ATP is an essential carrier of energy conversion in organisms and plays a vital role in many physiological processes of cells. ATP levels often decrease when cells are in apoptotic, necrotic, or toxic states (Osorio et al. 2003). In addition, NADH is primarily involved in electron transport, matter and energy metabolism in cells, and levels of NADH, NAD^+^ and NADH/NAD^+^ ratio can be used to evaluate the strength of glycolysis and TCA cycles (Hou et al. 2010). The decreased levels of micro fermentation parameters reflected the decrease in energy metabolism.

### Effect of gallic acid on *E. coli* W3110 cell morphology

**Fig. 3:**
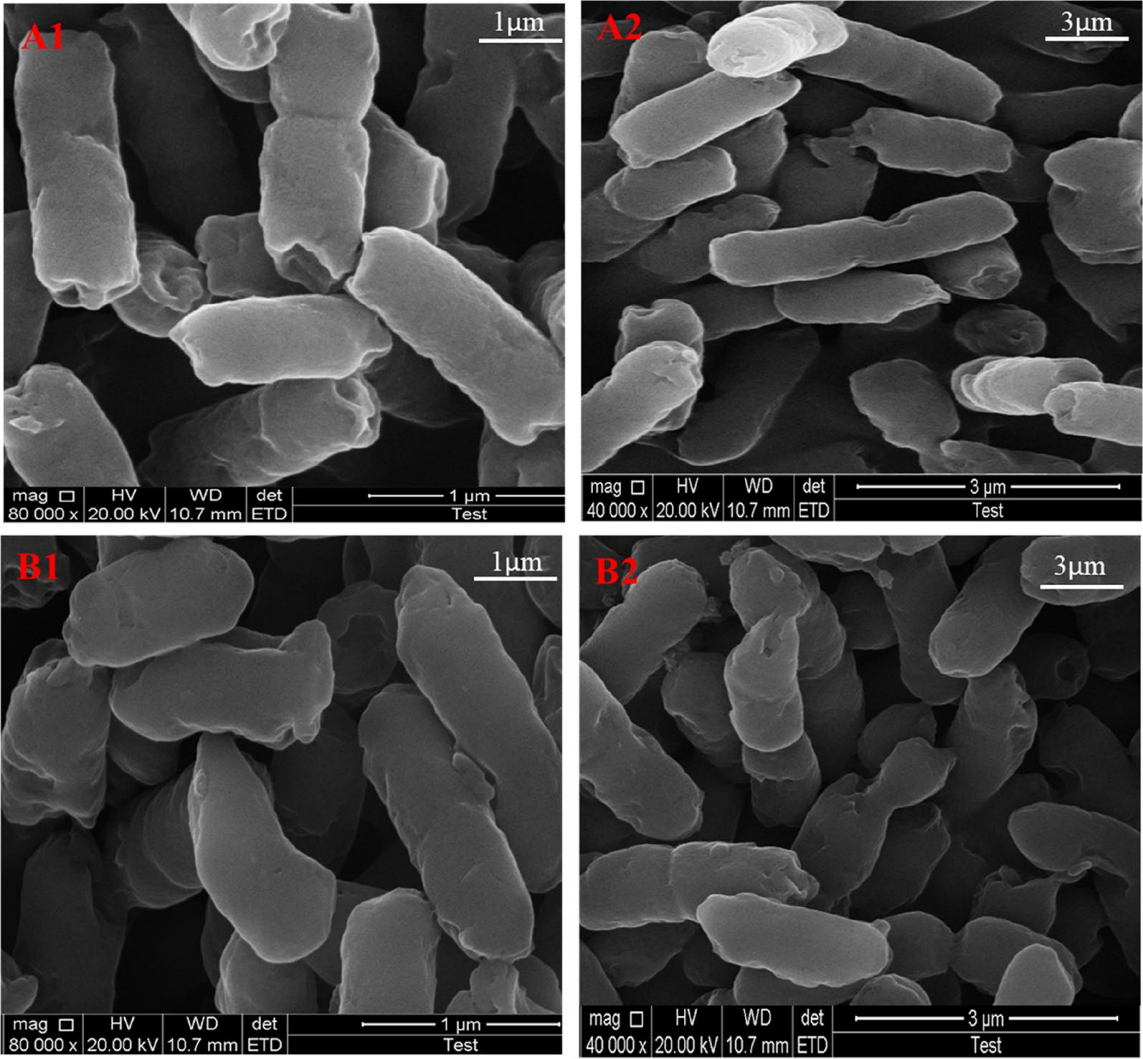
Scanning electron microscopy image of *E. coli* W3110 cells under gallic acid stress (gallic acid treatment) and fermentation without stress (control). (A1) Control (magnified 80,000 times); (A2) control (magnified 40,000 times); (B1) gallic acid treatment (magnified 80,000 times); (B2) gallic acid treatment (magnified 40,000 times)

Scanning electron microscopy was employed to study the morphological changes of *E. coli* W3110 cells under high concentrations (10 g/L) of gallic acid stress. The results showed that in the absence of gallic acid, the cell morphology of *E. coli* W3110 displayed regular short rods; however, under gallic acid stress, the *E. coli* W3110 cells showed a variety of irregular morphology (Fig. 3). It has been reported that gallic acid, as a phenolic compound, has a stress inhibition effect on *E. coli*, which can increase membrane fluidity and permeability, induce membrane leakage, and destroy its integrity (Mills et al. 2009). In this study, the *E. coli* W3110 cells showed a variety of irregular morphology with gallic acid stress (Fig. 3), which was consistent with the previously reported ones.

### Transcriptional analysis of *E. coli* W3110 response to gallic acid stress

#### Overall differential expression genes of *E. coli* W3110 under gallic acid stress

**Fig. 4:**
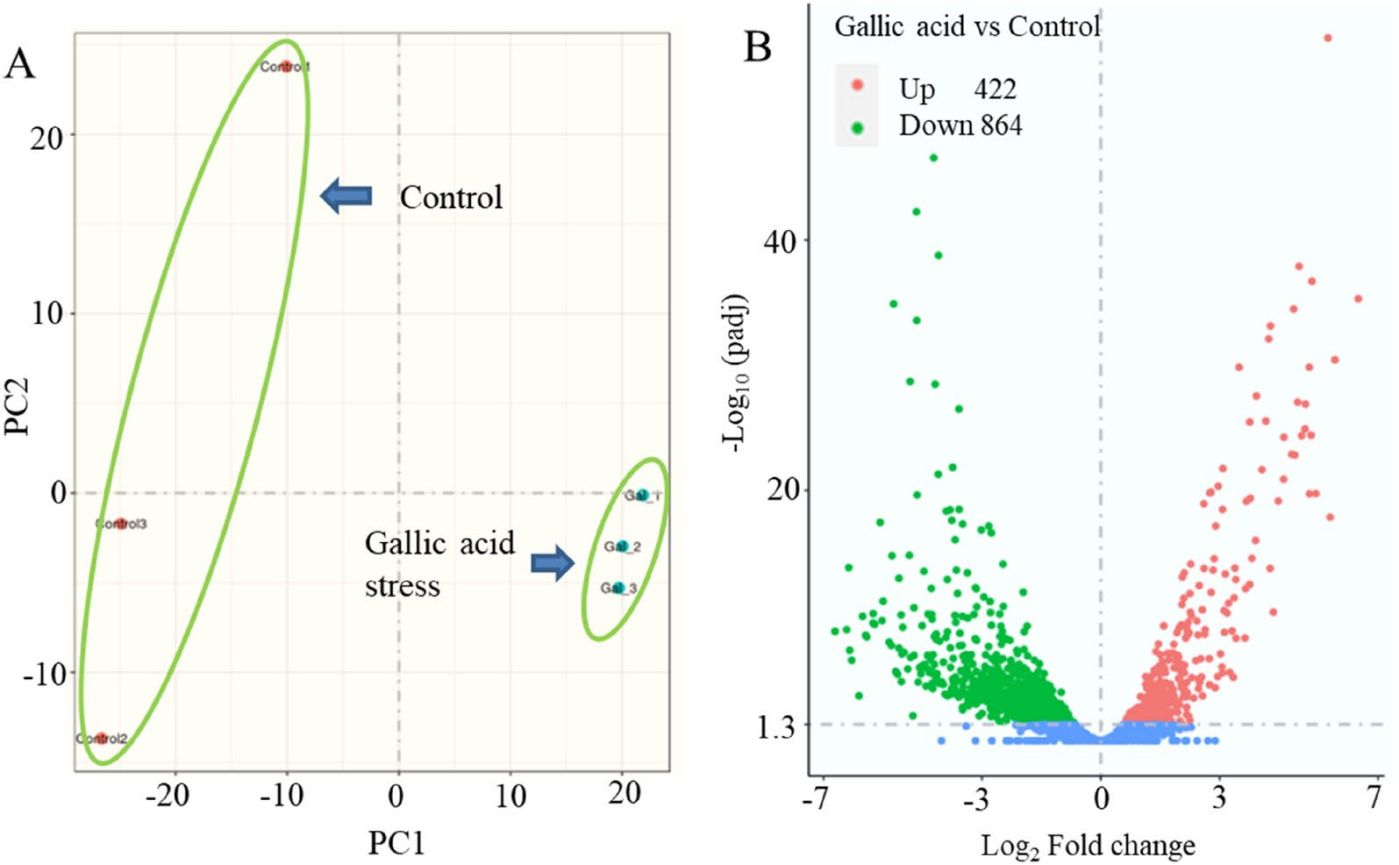
Principal component analysis of *E. coli* W3110 under gallic acid stress and fermentation without stress (control) (**A**). The Venn diagram of the down-regulated genes and up-regulated genes. (**B**)

**Fig. 5:**
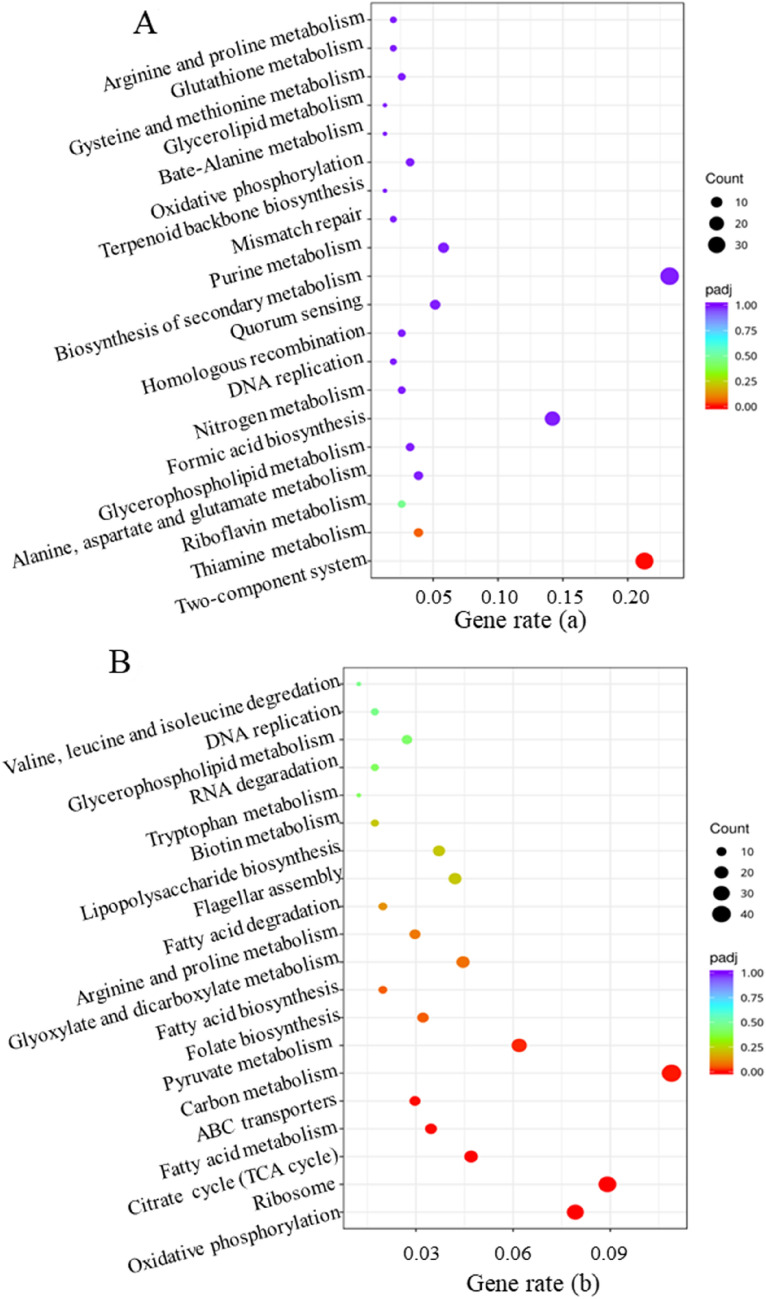
Metabolic pathway significance analysis of *E. coli* W3110 under gallic acid stress versus fermentation without stress. **A** Up-regulated metabolic pathways, gene rate (a) showed the ratio of differentially expressed genes (DEGs) in related pathways to total up-regulated DEGs; **B** down-regulated metabolic pathways, gene rate (b) showed the ratio of DEGs in related pathways to total down-regulated DEGs

To explore the gene transcription difference under gallic acid stress, transcriptomics analysis, which is the starting point for studying gene structure and function and can reveal the molecular mechanism of specific biological processes at the overall level (Ba et al. 2020), was carried out. Gene expression profiles of *E. coli* W3110 were generated from cell samples collected at 20 h. To ensure the reproducibility of the experiment, three biological replicates were conducted under the control (without gallic acid stress) and treatment (10 g/L gallic acid stress). The detailed list of differential expression genes (DEGs) in *E. coli* W3110 under stress fermentations was summarized in Additional file 1: Tables S1 and S2. According to a Pearson correlation analysis of the samples, under the same conditions (gallic acid stress or without gallic acid stress), there is a significant correlation between the samples, whereas there are significant differences between the samples under different treatment conditions (gallic acid stress versus without gallic acid stress) (Additional file 1: Fig. S2). Principal component analysis (PCA) was carried out to evaluate biological alteration based on the statistics. PCA is a multivariate technique to extract the critical information from data and displays the pattern of similarity of the observations and variables as points in maps. In the PCA score plot, the horizontal axis represents the first principal component, namely, PC1, which represents the maximum difference between groups; the vertical axis represents the second principal component, PC2, which represents the secondary differences between groups. The distances between the groups measure the overall variation among the gene expression profiles of the different strains. As shown in Fig. 4A, groups of the control and gallic acid treatment were distributed in different areas of the PCA score plot; groups of the control are in the negative region of PC1, while groups of gallic acid treatment are located in the positive region of PC1, indicating significant differences between the two groups. In the present study, 1286 genes showed different expression levels under gallic acid treatment compared to the control. To further analyze DEGs in samples, all genes (up-regulated and down-regulated) were displayed (Fig. 4B). Under gallic acid stress, 422 genes were up-regulated, while 864 genes were down-regulated compared to the cells without stress. Under gallic acid stress, the metabolic pathways in *E. coli* were considerably altered (Fig. 5). Based on the Kyoto Encyclopedia of Genes and Genomes (KEGG) database analysis, these DEGs are mainly concentrated in several metabolic pathways. Genes commonly up-regulated under gallic acid stress were involved in the two-component system (Fig. 5A). The down-regulated genes were involved in many metabolic pathways, including oxidative phosphorylation, citrate cycle (TCA cycle), pyruvate metabolism, carbon metabolism, fatty acid biosynthesis, ABC transporters, etc. (Fig. 5B). The detailed features of the metabolism of *E. coli* under gallic acid stress are discussed below by clustering some important metabolic pathways.

#### Two-component systems are altered under gallic acid stress

**Fig. 6:**
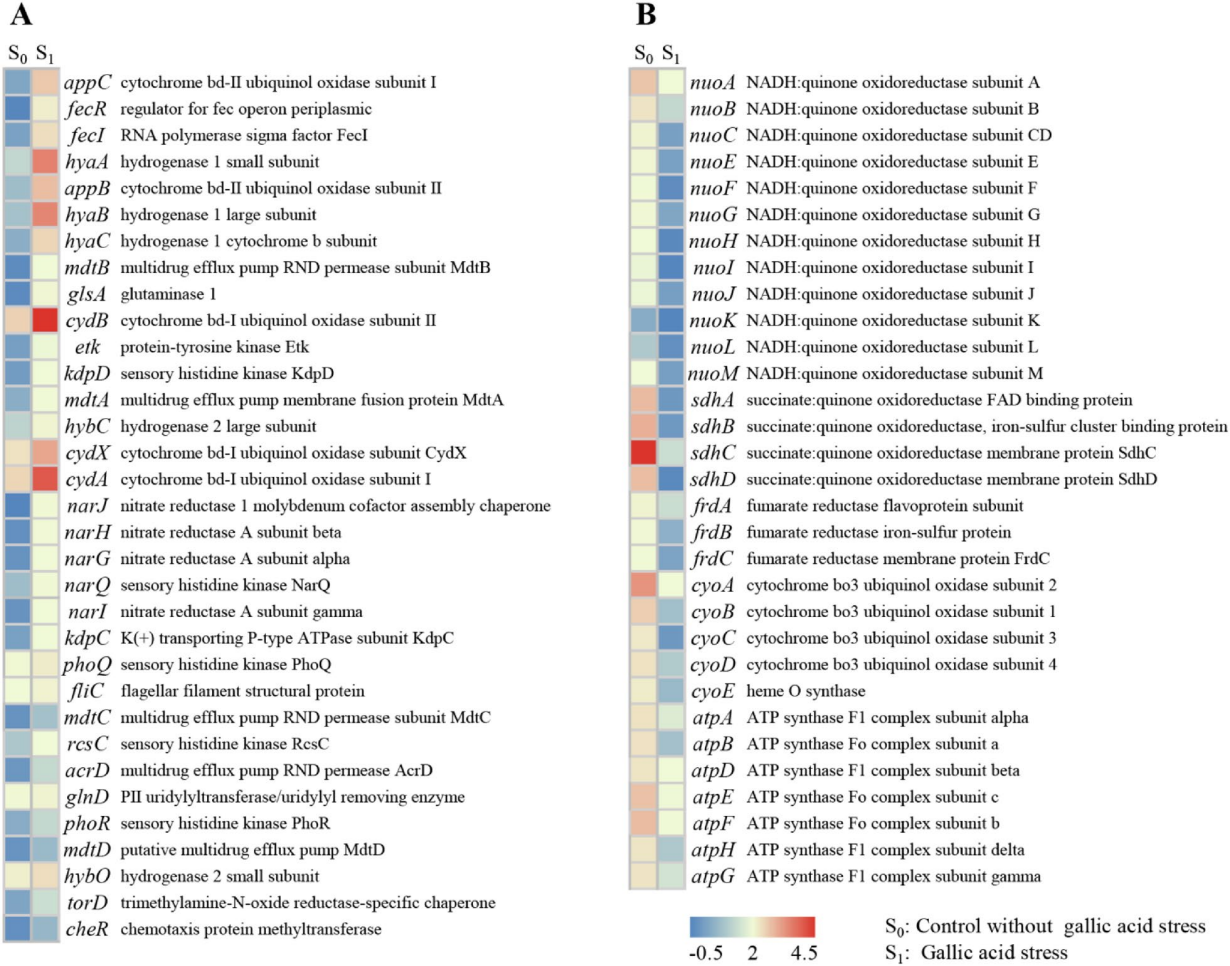
Heatmap of genes related to the **A** Two-component system, **B** energy metabolism. Z scores were utilized to show the transcription characteristics with a heatmap

Two-component systems (TCSs) are the most widespread sensory systems in bacteria that function in helping organisms respond quickly and accurately to various stimuli in the surrounding environment (Ba et al. 2020; Papon and Stock 2019). The DEGs associated with the two-component system showed up-regulated in heat maps under gallic acid stress involving many aspects, such as electron transport, chemotaxis, substance efflux, etc. (Fig. 6A). For example, some hydrogenase genes *hyaA*, *hyaB*, and *hyaC* were up-regulated by 35.1, 42.1, and 18.5-folds, respectively. Those three genes anchor hydrogenase to the cell membrane and conduct electrons to the membrane-bound electron transport chain, which is closely related to cellular energy metabolism (Voordouw 1992). Genes of *appC*, *appB*, *cydB*, *cydX*, and *cydA* encoding cytochrome bd-II ubiquinol oxidase are also associated with the transfer of electrons in the transport chain (Hoeser et al. 2014). Those five genes were up-regulated by 28.2, 23.8, 3.4, 2.0, and 3.0-folds, respectively. Moreover, gene *cheR* encoding a chemotaxis protein CheR was up-regulated by 1.1-fold (padj < 0.05). In addition to the above genes, some genes *mdtC*, *acrD*, and *mdtD* involved in multidrug efflux pumps were up-regulated by different degrees. The above results show that TCSs were altered under gallic acid stress. However, the response characteristics of TCSs in *E. coli* to gallic acid stress were still not elucidated thus far. Therefore, advanced system biology methods should be employed to illustrate the TCSs under gallic acid stress.

#### Influence of gallic acid stress on ABC transporter

Gallic acid stress for *E. coli* affected the expression level of genes involved in another membrane transporter system, the ATP-binding cassette (ABC)-transporter. ABC transporters are ubiquitous membrane proteins that use the energy of ATP hydrolysis to translocate solutes across cellular membranes (Hollenstein et al. 2007). In contrast to the gene expression characteristics of TCSs, it's worth noting that the 12 genes associated with ABC-transporter were down-regulated in heat maps under gallic acid stress. The down-regulated genes mainly involve genes encoding outer membrane proteins and genes encoding proteins involved in oligopeptide transport (Table 1). It was well known that OmpF encoding by gene *ompF* and OmpC encoding by gene *ompC* are two major outer membrane proteins in *E. coli* that serve as a barrier to hazardous substances entering the cell (Bhattacharjee et al. 2019). In this work, genes *ompF* and *ompC* were down-regulated by 31.1 and 8.1-folds, respectively. Previous reports suggest that the two major outer membrane proteins (OmpF and OmpC) are involved in non-specific solute transport (Igwe et al. 2016). The decreased expression levels of *ompF* and *ompC* may reduce the absorption of gallic acid, which is consistent with previous reports. Therefore, the expression levels of OmpF and OmpC can be reduced by genetic engineering to reduce the absorption rate of gallic acid by *E. coli* and enhance its tolerance. Finally, it is expected to construct a high yield gallic acid production strain. Opp systems related to the absorption of oligopeptides are sensitive to the amount of available peptide substrate in the surrounding medium (Jones et al., 2015). Genes of *oppA*, *oppC*, *oppD*, and *oppF* from Opp systems were down-regulated by 3.8, 2.4, 2.5, 2.0, and 3.6-folds, respectively. Carbon and nitrogen availability in the culture medium can change the oppA promoter activity and alter the expression of Opp systems (Wang et al. 2002). In this work, carbon and nitrogen availability was changed by adding another carbon source (10 g/L gallic acids), so the decreased expression levels of Opp systems may adapt *E. coli* to the changing environment (Table 1).

**Table 1:** Down-regulated DEGs involved in ABC transporter of *E. coli* W3110 under gallic acid stress versus fermentation without stress

Number	Gene ID	Gene	Fold^a^	Gene description
1	b0929	*ompF*	31.1	Outer membrane porin F
2	b2215	*ompC*	8.1	Outer membrane porin C
3	b1243	*oppA*	3.8	Oligopeptide ABC transporter periplasmic binding protein
4	b1247	*oppF*	3.6	Murein tripeptide ABC transporter/oligopeptide ABC transporter ATP binding subunit OppF
5	b1245	oppC	2.4	Murein tripeptide ABC transporter/oligopeptide ABC transporter inner membrane subunit OppC
6	b0463	*acrA*	3.2	Multidrug efflux pump membrane fusion lipoprotein AcrA
7	b0433	*ampG*	0.9	Muropeptide:H( +) symporter
8	b1246	*oppD*	2.5	Murein tripeptide ABC transporter/oligopeptide ABC transporter ATP binding subunit OppD
10	b3035	*tolC*	1.9	Outer membrane channel TolC
11	b0462	*acrB*	2.4	Multidrug efflux pump RND permease AcrB
12	b1329	*mppA*	1.5	Murein tripeptide ABC transporter periplasmic binding protein

^a^Down-regulated folds

#### Expression profiles of genes involved in energy metabolism

It is well-known that the primary cellular resources NADH and ATP are vital for cell physiological metabolism (Wenk et al. 2020). Cofactor NADH is involved in ATP synthesis during oxidative phosphorylation (ecj00190), which includes five parts NADH oxidoreductase (complex I), succinate oxidoreductase/fumarate reductase (complex II), cytochrome bc1complex (complex III), cytochrome c oxidase (complex IV), ATP synthase (complex V). As shown in Fig. 6B, 12 NADH oxidoreductase encoding genes of *nuoA*, *nuoB*, *nuoC*, *nuoE*, *nuoF*, *nuoG*, *nuoH*, *nuoI*, *nuoJ*, *nuoK*, *nuoL*, and *nuoM* were down-regulated by 1.4–10.9 folds; 4 succinate oxidoreductase encoding genes of *sdhA*, *sdhB*, *sdhC*, and *sdhD* were down-regulated by 45.0–63.6 folds; 3 fumarate reductase encoding genes of *frdA*, *frdB* and *frdC* were down-regulated by 2.2–2.5 folds; 5 cytochrome oxidase coding genes of *cyoA*, *cyoB*, *cyoC*, *cyoC*, and *cyoE* were down-regulated by 6.7–21.8 folds; 7 ATP synthase coding genes of *atpA*, *atpB*, *atpD*, *nuoE*, *atpF*, *atpH*, and *atpG* were down-regulated by 5.9–12.2 folds. Most of the genes associated with energy metabolism were downregulated to varying degrees (Fig. 5B), suggesting that the capacity to decompose carbohydrates and produce energy was decreased under gallic acid stress (Li et al. 2021).

#### Effects of gallic acid stress on carbon metabolism

**Fig. 7:**
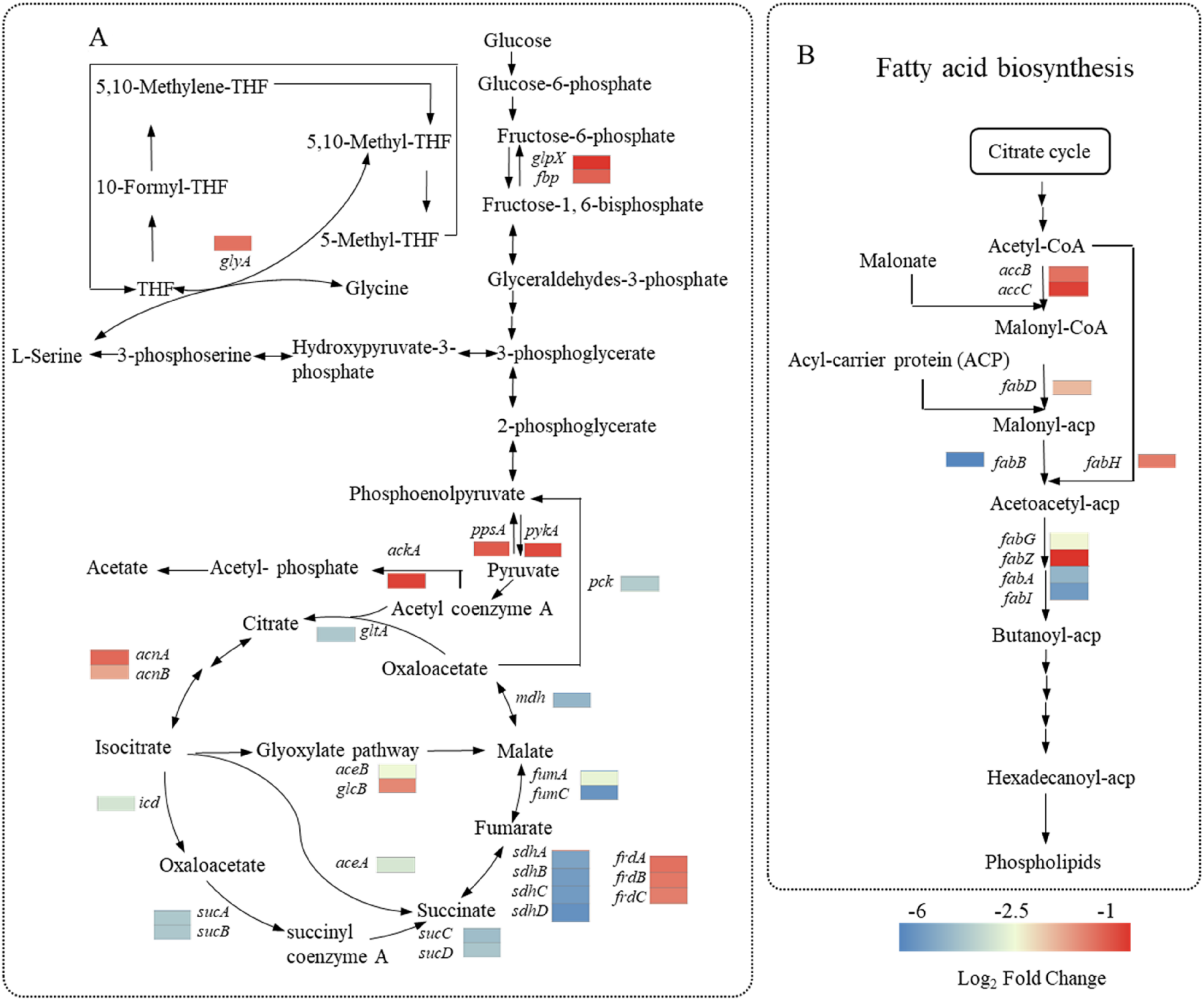
Expression profiles of selected genes of *E. coli* W3110 cells under gallic acid stress versus fermentation without stress. Genes related to carbon metabolism (**A**) and fatty acid biosynthesis (**B**)

Gallic acid stress for *E. coli* also affected the expression of genes involved in carbon metabolism. According to KEGG pathway analysis and statistical analysis of DEGs, 27 genes were down-regulated in carbon metabolism (ecj01200) with gallic acid stress (Fig. 5B). As shown in Fig. 7A, the down-regulated genes covered six metabolic pathways: glycolysis, gluconeogenesis, tricarboxylic acid (TCA) cycle, glyoxylate pathway, and acetate and tetrahydrofolate biosynthesis pathway. In gluconeogenesis, *fbp*, *glpX*, and *ppsA* encode fructose-1,6-bisphosphatase I, fructose-1,6-bisphosphatase II phosphoenolpyruvate synthase were down-regulated with 1.9-, 1.1-, and 1.8-fold (padj < 0.05), respectively; meanwhile, gene *pykA* encoding pyruvate kinase II from glycolysis was down-regulated with 1.4-fold (padj < 0.05). Moreover, it's worth noting that most genes *(gltA*, *acn*, *icd*, *sucA*, *sdhA*, and *mdh*, etc.) from the TCA cycle and genes (*aceB* and *glcB*) from the glyoxylate pathway were downregulated to varying degrees. Those down-regulated genes encode the enzymes associated with the TCA cycle and glyoxylate pathway, suggesting that metabolic flow from the two metabolic pathways may be down-regulated.

In addition to the above metabolic pathway, the acetate biosynthesis pathway and tetrahydrofolate biosynthesis pathway were also affected. Acetate is a common by-product during *E. coli* fermentation process (Liu et al. 2016a). In the acetate biosynthesis pathway, the gene *ackA* was down-regulated by 1.3-fold (padj < 0.05), consistent with the reduced acetate concentration under gallic acid stress (Fig. 2B). Serine hydroxymethyltransferase catalyzes the formation of tetrahydrofolate, an essential intermediate metabolite, which plays a vital role in the metabolism of genetic material and protein (Mishra et al. 2019). The gene *glyA* encoding serine hydroxymethyltransferase was down-regulated by 2.3-fold. The down-regulated gene *glyA* could affect tetrahydrofolate formation and further effectively block the physiological metabolism of cells. The physiology of E. coli rapidly adapts to changes in environmental variables. In response to environmental stress, *E. coli* often adopts the general energy conservation strategy, reflecting that the rapid decrease of central carbon metabolism intermediates coincided with the downregulation of genes related to cell growth (Jozefczuk et al. 2014).

#### Fatty acid biosynthesis is altered under gallic acid stress

In cells, fatty acid biosynthesis is closely related to the biosynthesis of phospholipids in cell membranes, and the primary use for fatty acids is in phospholipid biosynthesis (Satoh et al. 2020; Thomanek et al. 2019). Under gallic acid stress, the genes involved in fatty acid biosynthesis significantly changed (Fig. 5B). To explore the effect of gallic acid on membrane lipid biosynthesis, the expression levels involved in the fatty acid biosynthesis pathway (ecj01212) were compared (Fig. 7B). We observed selective down-regulation of genes involved in fatty acid biosynthesis, such as the genes (*accB* and *accC*) encoding acetyl-coenzyme A (acetyl-CoA)/propionyl-CoA carboxylase (ACC), gene *fabH* encoding ketolipoyl ACP synthase, gene *fabD* encoding malonyl-CoA-acyl carrier protein transacylase (FabD), and four genes (*fabG*, *fabZ*, *fabA*, and *fabI*) associated in lipoyl chain extension (Feng et al. 2008). Fatty acid biosynthesis is tightly regulated at multiple levels. ACC encoded by *accA*, *accB*, *accC* and *accD* is one of the key rate-limiting enzymes which catalyze the formation of malonyl-CoA as a precursor in fatty acid biosynthesis (Cronan 2021). In this study, gene*s accB* and *accC* were down-regulated by 2.2- and 2.6-fold, respectively. In addition, genes *fabH* and *fabI* encoding two components of the fatty acid synthase FabH and FabI, which are feedback inhibited by long-chain fatty acyl-ACPs (Hill et al. 1995), were down-regulated by 2.5- and 52.7-fold, respectively. Moreover, other genes associated in fatty acid biosynthesis, such as *fabD*, *fabG*, *fabZ* and *fabA*, were down-regulated by 4.5-, 7.5-, 3.9-, 1.1-, and 31.8-fold, respectively. These results demonstrated that gallic acid stress strongly affects fatty acid biosynthesis possibly hindering membrane lipid biosynthesis. It was reported that the enhancement of the TCA cycle and fatty acid biosynthesis potentially increased the cellular membrane permeability, resulting in cellular structure autolysis (Green et al. 2014). In contrast, the down-regulated fatty acid biosynthesis and TCA cycle in this study may contribute to increased cell membrane resiliency to gallic acid stress. Considering that membrane lipid biosynthesis is closely related to the integrity and fluidity of the cell membrane (Qi et al. 2017), inhibition of membrane phospholipid biosynthesis alters the composition and function of the cell membrane; thus, future affects the cell survival under stress condition (Yan et al. 2016).

### Gene ontology analysis

**Fig. 8:**
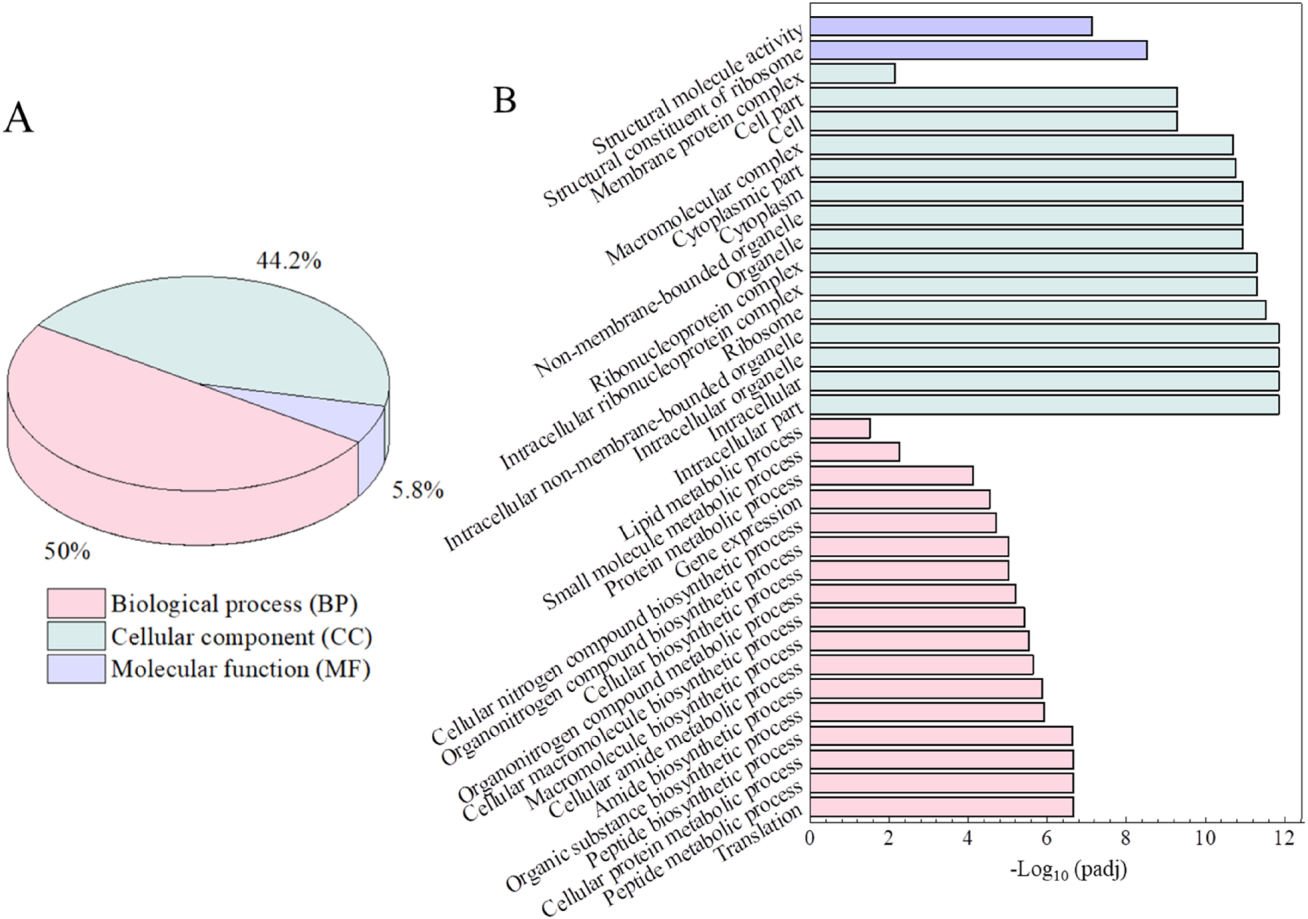
Gene ontology analysis of DEGs of *E. coli* W3110 under gallic acid stress versus fermentation without stress. **A** Proportion of BP, CC, MF. **B** Gene function classification and padj analysis

Gene function analysis refers to the prediction, identification and verification of gene function using bioinformatics and different expression systems (Marzorati et al. 2021), which has been employed to explore metabolic mechanisms inside cells (Che et al. 2020; Lin et al. 2017). Gene Ontology (GO) is a comprehensive database describing gene functions, which can be divided into three parts: biological process (BP), cellular component (CC), and molecular function (MF). The threshold of GO enrichment analysis was padj < 0.05. Statistical analysis of the metabolic pathways involving the DEGs was performed using GO mapping, which covers 34 functional areas. It is worth noting that most of the genes involved in the 34 functional areas are down-regulated, and only a few are up-regulated (Additional file 1: Table S3). Among all functional areas, the parts of BP, CC, and MF showed different proportions, which accounted for 50.0%, 44.2%, and 5.8%, respectively (Fig. 8A). The set of DEGs involved in the biological process plays an important role in different biological processes, such as acting in varieties of translation (GO:0006412), peptide metabolic process (GO:0006518), cellular protein metabolic process (GO:0044267), etc. (Fig. 8B). The cellular component analysis revealed that some DEGs associated with intracellular part (GO:0044424), intracellular organelle (GO:0043229), intracellular ribonucleoprotein complex (GO:0030529), etc. In addition, some DEGs are involved in the structural constituent of ribosome (GO:0003735) and structural molecule activity (GO:0005198), which have notable molecular functions (Fig. 7B).

## Discussion

This study investigated the toxic effects of gallic acid stress on *E. coli* W3110, a commonly used strain for producing high value-added products by engineering modification, by analyzing the levels of macro fermentation parameters and micro fermentation parameters. With an increase in gallic acid concentration, the final cell biomass, total glucose consumption, and acetate level for macro fermentation parameters all gradually dropped (Fig. 2A–C). In addition to the macro fermentation parameters, the levels of micro fermentation parameters (NADH, NAD^+^, NADH/ NAD^+^ and ATP) in *E. coli* W3110 decreased, respectively, under high concentrations of gallic acid (Fig. 2D, E). In addition, a high concentration (10 g/L) of gallic acid stress resulted in changes in the cell morphology of *E. coli* (Fig. 3). Based on the fermentation parameters, gallic acid showed a higher inhibitory effect on the physiological metabolism of *E. coli.*

Gene transcriptomics was employed to explore the specific effect of gallic acid on the internal metabolism of *E. coli*, which showed a total of 1286 differential gene expression genes (422 genes up-regulated and 864 genes down-regulated) under gallic acid stress versus fermentation without stress (Fig. 4B). Statistical analysis of the metabolic pathways involving the identified differentially regulated genes indicated that gallic acid altered expression profiles of genes related to five notable differentially regulated pathways. The genes associated with the two-component system were up-regulated, while the genes pertaining to ABC-transporter, energy metabolism, carbon metabolism, and fatty acid biosynthesis were down-regulated. The two-component system and ABC-transporter are closely related to the transport of substances. Two-component systems (TCSs) are the most widespread sensory systems in bacteria that enables organisms to respond quickly and precisely to a variety of environmental stimuli (Ba et al. 2020; Papon and Stock 2019). The DEGs associated with the two-component system showed up-regulated (Fig. 5A). For ABC-transporter, the most significant change was the decreased expression levels of gene *ompF* and *ompC*, which decreased by 31.1 and 8.1-folds, respectively (Table 1). Two major outer membrane proteins (OmpF and OmpC) encoding by gene *ompF* and *ompC*, respectively, involved in transporting non-specific substances act as barriers to prevent harmful substances from entering the cell (Igwe et al. 2016). *Serratia marcescens*, a Gram-negative enterobacterium, such as *E. coli*, own a high degree of natural antibiotic resistance due to its reduction of outer membrane permeability, which is partially regulated by OmpF and OmpC (Begic and Worobec 2005). Therefore, the decreased expression levels of ompF and ompC may reduce the absorption of gallic acid. Thus, the expression levels of OmpF and OmpC can be reduced by genetic engineering to reduce the absorption rate of gallic acid by *E. coli* and enhance its tolerance.

Energy metabolism, carbon metabolism, and fatty acid biosynthesis involve the metabolism of substances inside cells. In this study, genes involved in energy metabolism, carbon metabolism, and fatty acid biosynthesis were down-regulated. TCA cycle is a hub of energy metabolism and connects protein, carbohydrate, and fat metabolism (Noronha et al. 2015). Moreover, *E. coli* often adopts the general energy conservation strategy under environmental stress, reflecting that the rapid decrease of central carbon metabolism intermediates coincided with the downregulation of genes related to cell growth (Jozefczuk et al. 2014). In this work, suppressed carbon metabolism and decreased biomass under gallic acid stress were consistent with previous reports. Since fatty acid biosynthesis requires a great deal of reducing power, such as NADH or NADPH (Li et al. 2018), the down-regulation of energy metabolism could affect the synthesis of fatty acids. In conclusion, the response mechanism of *E. coli* under gallic acid stress can be divided into two parts. The first is the change of signal transduction process caused by external environmental factors, which affects the cell membrane transport system, finally reducing the absorption of gallic acid. Second, the entry of gallic acid into the cell led to the down-regulation of matter and energy metabolism, involving energy metabolism, carbon metabolism, and fatty acid biosynthesis, which is a protective mechanism of microorganisms in adverse environments.

In addition, GO enrichment analysis showed that the differential expression genes were involved in biological processes, cellular components, and molecular function (Fig. 8), which may suggest that the presence of gallic acid may affect the physiological function of cells through various pathways after entering cells, such as regulating the biosynthesis pathway, changing the synthesis of cell components, affecting the normal play of molecular functions. In this study, it should be noted that genome-wide transcriptomics was only applied to omics technology, which could not accurately indicate the intracellular levels of essential proteins. For providing key inhibitory targets of *E. coli* under gallic acid stress, transcriptomics, proteomics, and even metabolomics should be applied.

With the gradual maturity of genetic modification technology, the construction of heterologous synthesis pathways to improve the yield of the target product has become a common method. However, the yield of gallic acid by *E. coli* fermentation is still low. Gallic acid is a phenolic compound which has a stress inhibition effect on *E. coli*. Therefore, improving the tolerance of *E. coli* to phenolic compounds may be the key problem for yield improvement. The cell membrane acts as a barrier for microbes to cope with adverse environments. Therefore, regulating the expression of outer membrane proteins, such as reducing the expression of OmpF and OmpC and thus decreasing the uptake of extracellular phenolic compounds, may contribute to improving the resistance of *E. coli* under phenolic compounds stress. Moreover, how do phenolic compounds affect the metabolic pathway after entering *E. coli*? Although transcriptomics was used to study the effect of gallic acid on gene levels in *E. coli*, this study was not systematic enough to show the changes in the content of metabolic intermediates and protein levels. Therefore, it is necessary to explore the intracellular metabolism of *E. coli* under the stress of phenolic compounds through the comprehensive application of genomics, transcriptomics, and proteomics, to identify genetic modification targets. Then, phenolic compounds producing strain of *E. coli* with high productivity should be constructed by targeted genetic modification.

### Supplementary Information

**Additional file 1: Fig. S1**. Effects of gallic acid on the cell membrane and intracellular components of E. coli. **Fig. S2**. Pearson correlation between samples. Control-1, Control-2, and Control-3 were three samples of E. coli W3110 fermentation without gallic acid stress; Gallic acid-1, Gallic acid-2, and Gallic acid-3 were three samples under gallic acid stress. **Table S1**. Differential gene expression genes (up-regulated) of E. coli W3110 under gallic acid stress versus fermentation without stress. **Table S2**. Differential gene expression genes (down-regulated) of E. coli W3110 under gallic acid stress versus fermentation without stress. **Table S3**. Gene Ontology analysis of DEGs.

## Data Availability

All data and materials are included in the article.
