# Supplementary material for: Toxicity and inhibition mechanism of gallic acid on physiology and fermentation performance of Escherichia coli
Source: Bioresour Bioprocess. 2022 Jul 23;9(1):76. doi: 10.1186/s40643-022-00564-w (PMC10992115; doi:10.1186/s40643-022-00564-w)
Supplement: Supplementary file 1 — Additional file 1: Fig. S1. Effects of gallic acid on the cell membrane and intracellular components of E. coli. Fig. S2. Pearson correlation between samples. Control-1, Control-2, and Control-3 were three samples of E. coli W3110 fermentation without gallic acid stress; Gallic acid-1, Gallic acid-2, and Gallic acid-3 were three samples under gallic acid stress. Table S1. Differential gene expression genes (up-regulated) of E. coli W3110 under gallic acid stress versus fermentation without stress. Table S2. Differential gene expression genes (down-regulated) of E. coli W3110 under gallic acid stress versus fermentation without stress. Table S3. Gene Ontology analysis of DEGs. [file 40643_2022_564_MOESM1_ESM.doc]

**Toxicity and inhibition mechanism of gallic acid on** **physiology and fermentation performance of *Escherichia coli***

Lina Liu ^1^*, Xiaolong Ma^1^, Muhammad Bilal ^1^, Linlin Wei ^1^, Shijie Tang ^1^, Hongzhen Luo ^1^, Yuping Zhao ^1^, Zhaoyu Wang ^1^, and Xuguo Duan ^2^

^1^ School of Life Science and Food Engineering, Huaiyin Institute of Technology, Huaian 223003, China.

linaliu@hyit.edu.cn (L.L.); [1371969978@qq.com](mailto:1371969978@qq.com) (X.M.); bilaluaf@hyit.edu.cn (M.B.); [2937046918@qq.com](mailto:2937046918@qq.com) (L.W.); [shijie_tang0621@163.com](mailto:shijie_tang0621@163.com); hzluo@hyit.edu.cn (H.L.); zhaoyuping@hyit.edu.cn (Y.Z.); biowzy@hyit.edu.cn;

^2^College of Light Industry and Food Engineering, Nanjing Forestry University, Nanjing, Jiangsu 210037, China; [xguduan@njfu.edu.cn](mailto:xguduan@njfu.edu.cn) (X.D.)

***** Correspondence: linaliu@hyit.edu.cn (L.L.); Tel: +86-13852349408; Fax: +86- 517-83559216

This supplementary material is sent to the journal of Bioresources and Bioprocessing.


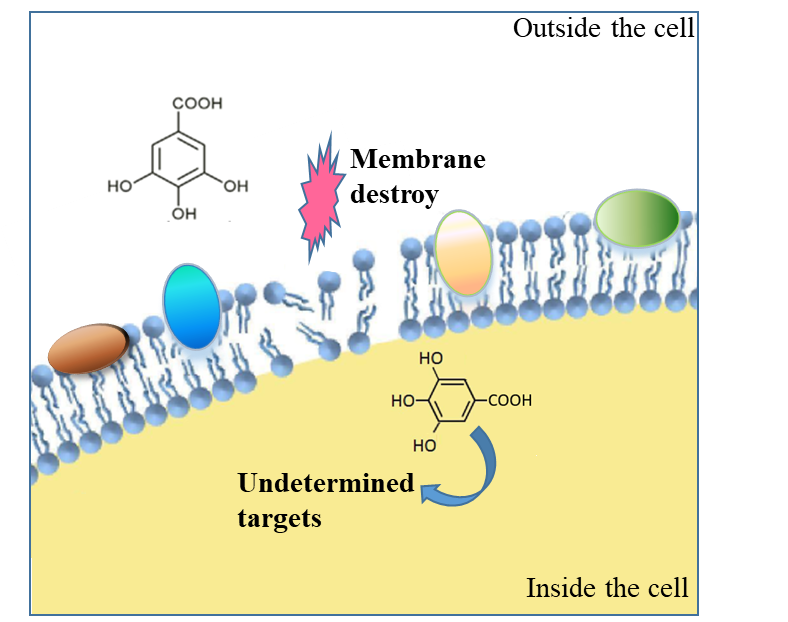


**Fig. S1.** Effects of gallic acid on the cell membrane and intracellular components of *E. coli.*


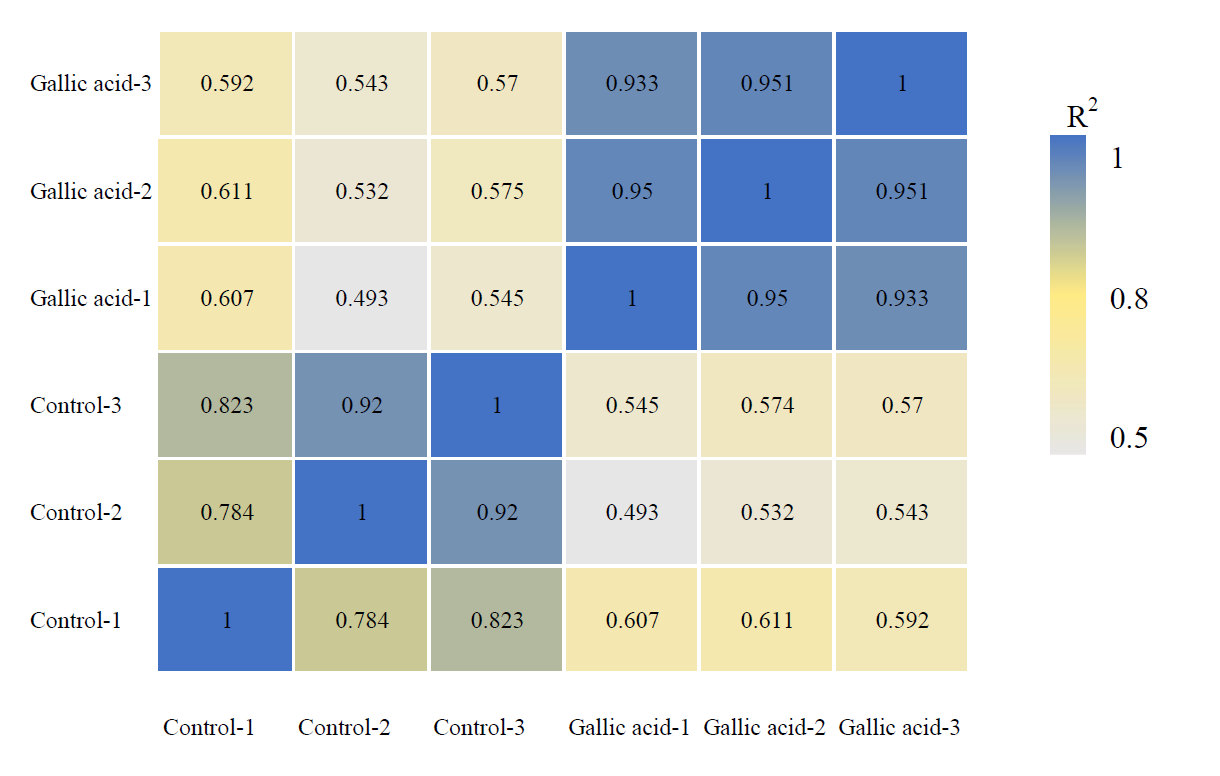


**Fig. S2.** Pearson correlation between samples. Control-1, Control-2, and Control-3 were three samples of *E. coli* W3110 fermentation without gallic acid stress; Gallic acid-1, Gallic acid-2, and Gallic acid-3 were three samples under gallic acid stress

Table S1. Differential gene expression genes (up-regulated) of *E. coli* W3110 under gallic acid stress versus fermentation without stress.

| Gene  id | Gene  name | Treatment-1 | Treatment-2 | Treatment-3 | Control-1 | Control-2 | Control-3 | padj |
| --- | --- | --- | --- | --- | --- | --- | --- | --- |
| b3513 | *mdtE* | 3474.686 | 2925.653 | 4343.971 | 86.00806 | 59.68346 | 53.38185 | 7.13E-57 |
| b3510 | *hdeA* | 4723.888 | 5838.379 | 7619.418 | 222.4346 | 213.4832 | 128.3217 | 1.28E-38 |
| b3506 | *slp* | 6777.195 | 7375.542 | 9808.872 | 230.061 | 247.9159 | 116.0029 | 2.00E-37 |
| b4718 | *gadF* | 537.5964 | 478.3437 | 601.2265 | 8.050015 | 1.147759 | 7.186018 | 4.93E-36 |
| b0978 | *appC* | 4239.41 | 4078.849 | 5387.747 | 190.6583 | 182.4937 | 95.47138 | 3.21E-35 |
| b4292 | *fecR* | 792.1992 | 914.0243 | 1020.484 | 57.62116 | 33.28501 | 47.2224 | 7.18E-34 |
| b4293 | *fecI* | 799.5259 | 948.9304 | 1003.015 | 35.58954 | 61.97898 | 50.30212 | 7.88E-33 |
| b0849 | *grxA* | 3972.902 | 3831.921 | 4026.616 | 34.74217 | 113.6281 | 50.30212 | 3.73E-31 |
| b0977 | *hyaF* | 2923.352 | 2770.515 | 4310.488 | 108.0397 | 105.5938 | 46.19583 | 1.46E-30 |
| b4374 | *yjjG* | 1454.349 | 1378.147 | 1575.126 | 115.666 | 165.2773 | 112.9231 | 1.46E-30 |
| b2142 | *yohK* | 958.8815 | 1314.799 | 1189.351 | 98.29492 | 64.2745 | 62.62101 | 2.81E-28 |
| b4115 | *adiC* | 4083.718 | 3967.667 | 5236.348 | 206.7583 | 137.7311 | 76.99305 | 8.76E-28 |
| b0972 | *hyaA* | 6373.311 | 6629.585 | 9656.018 | 316.9164 | 192.8235 | 118.056 | 1.25E-27 |
| b3514 | *mdtF* | 4081.886 | 3931.468 | 5114.065 | 343.6085 | 169.8683 | 213.5274 | 2.85E-26 |
| b4116 | *adiY* | 544.9231 | 677.4381 | 841.426 | 57.19748 | 52.79691 | 41.06296 | 3.46E-26 |
| b0976 | *hyaE* | 1841.749 | 1575.949 | 2368.512 | 52.11326 | 83.7864 | 26.69092 | 1.24E-25 |
| b0975 | *hyaD* | 2391.251 | 2449.895 | 3624.828 | 102.1081 | 78.04761 | 31.82379 | 3.87E-25 |
| b0980 | *appA* | 4644.21 | 4568.829 | 6616.403 | 170.7451 | 224.9608 | 75.96647 | 4.19E-25 |
| b0979 | *appB* | 3588.25 | 3647.047 | 4738.48 | 251.6689 | 113.6281 | 118.056 | 5.60E-25 |
| b4592 | *appX* | 216.1376 | 252.1 | 292.6066 | 11.4395 | 10.32983 | 4.106296 | 1.33E-23 |
| b1493 | *gadB* | 327.8697 | 355.5257 | 540.0848 | 17.79477 | 14.92087 | 7.186018 | 1.50E-23 |
| b4372 | *holD* | 975.3666 | 1060.113 | 1256.316 | 106.3449 | 157.243 | 127.2952 | 1.80E-22 |
| b2141 | *yohJ* | 715.2688 | 798.9632 | 847.249 | 67.36592 | 41.31932 | 29.77065 | 2.26E-22 |
| b1492 | *gadC* | 3981.144 | 4586.928 | 5940.933 | 288.1058 | 200.8578 | 100.6042 | 1.29E-21 |
| b0589 | *fepG* | 606.2842 | 704.5873 | 687.116 | 66.94223 | 96.41175 | 94.44481 | 4.69E-21 |
| b4373 | *rimI* | 760.1449 | 744.6648 | 885.0986 | 98.29492 | 137.7311 | 112.9231 | 1.39E-20 |
| b1379 | *hslJ* | 348.934 | 333.5478 | 390.1421 | 57.19748 | 50.50139 | 50.30212 | 1.58E-20 |
| b0973 | *hyaB* | 9433.123 | 9760.797 | 15499.42 | 319.0348 | 389.0903 | 96.49795 | 1.81E-20 |
| b3509 | *hdeB* | 1459.844 | 1510.015 | 2123.946 | 66.09486 | 49.35363 | 15.39861 | 1.89E-20 |
| b4060 | *yjcB* | 1692.467 | 1913.375 | 2170.53 | 83.46595 | 176.7549 | 164.2518 | 4.21E-20 |
| b3507 | *dctR* | 334.2806 | 340.0119 | 486.2219 | 23.72636 | 8.034313 | 18.47833 | 7.17E-20 |
| b3686 | *ibpB* | 2831.769 | 3219.124 | 3652.488 | 363.9454 | 219.222 | 174.5176 | 7.45E-20 |
| b2143 | *cdd* | 970.7874 | 1088.555 | 1195.174 | 208.0293 | 161.834 | 163.2253 | 1.17E-19 |
| b0484 | *copA* | 2628.453 | 3193.267 | 3800.974 | 482.1535 | 346.6232 | 312.0785 | 3.34E-19 |
| b3512 | *gadE* | 7342.267 | 6345.164 | 8792.756 | 91.93965 | 267.4278 | 47.2224 | 1.39E-18 |
| b1000 | *cbpA* | 2068.876 | 2589.52 | 3260.89 | 410.5508 | 353.5098 | 295.6533 | 7.09E-18 |
| b1380 | *ldhA* | 1177.767 | 1323.848 | 1776.02 | 72.87382 | 150.3564 | 63.64759 | 1.01E-16 |
| b3241 | *aaeA* | 896.6046 | 1044.599 | 1251.949 | 117.7844 | 43.61484 | 62.62101 | 2.67E-15 |
| b4291 | *fecA* | 1644.844 | 1720.744 | 2000.206 | 326.2375 | 177.9026 | 230.9791 | 2.84E-15 |
| b2474 | *tmcA* | 1159.45 | 1115.704 | 1217.011 | 283.0216 | 195.119 | 244.3246 | 7.82E-15 |
| b3240 | *aaeB* | 1213.484 | 1325.141 | 1499.427 | 181.3372 | 72.30881 | 129.3483 | 1.62E-14 |
| b0974 | *hyaC* | 1428.706 | 1422.103 | 1976.914 | 146.595 | 48.20588 | 52.35527 | 1.71E-14 |
| b0606 | *ahpF* | 4217.43 | 4461.524 | 5804.092 | 596.1248 | 918.2071 | 862.3221 | 1.71E-14 |
| b2592 | *clpB* | 27275.46 | 30752.33 | 33328.04 | 3594.12 | 5935.061 | 3259.372 | 2.37E-14 |
| b0586 | *entF* | 1180.514 | 1224.301 | 1425.183 | 254.6347 | 322.5203 | 315.1582 | 3.86E-14 |
| b3242 | *aaeX* | 278.4145 | 292.1775 | 298.4296 | 40.25008 | 18.36414 | 37.98324 | 4.75E-14 |
| b0590 | *fepD* | 1608.21 | 1705.231 | 1749.817 | 337.6769 | 462.5469 | 417.8156 | 8.25E-14 |
| b1967 | *hchA* | 804.105 | 985.1294 | 1184.984 | 58.46853 | 141.1743 | 82.12592 | 1.32E-13 |
| b0699 | *ybfA* | 1986.451 | 2977.366 | 2573.774 | 94.48176 | 292.6785 | 171.4379 | 3.18E-13 |
| b2476 | *purC* | 619.1059 | 853.2617 | 992.8244 | 144.4766 | 165.2773 | 131.4015 | 3.97E-13 |
| b3511 | *hdeD* | 999.1784 | 1193.274 | 1263.595 | 152.5266 | 59.68346 | 59.54129 | 7.16E-13 |
| b2392 | *mntH* | 3602.903 | 4495.138 | 4183.838 | 395.7218 | 812.6133 | 571.8017 | 1.38E-12 |
| b3024 | *ygiW* | 1075.193 | 1418.224 | 1481.958 | 235.9925 | 325.9635 | 275.1218 | 1.74E-12 |
| b4704 | *arrS* | 74.18281 | 81.44771 | 82.97799 | 8.4737 | 8.034313 | 6.159444 | 3.81E-12 |
| b0585 | *fes* | 401.1367 | 438.2662 | 529.8946 | 97.87124 | 114.7759 | 108.8168 | 4.01E-12 |
| b4117 | *adiA* | 1010.168 | 1022.621 | 1503.794 | 193.2004 | 91.82071 | 90.33851 | 1.04E-11 |
| b2289 | *lrhA* | 1697.962 | 1722.037 | 1953.622 | 349.1164 | 517.6393 | 433.2142 | 1.32E-11 |
| b0152 | *fhuD* | 491.8046 | 620.554 | 756.9922 | 76.68699 | 117.0714 | 124.2155 | 3.47E-11 |
| b2557 | *purL* | 2095.435 | 2208.138 | 2838.721 | 403.3481 | 152.6519 | 268.9624 | 3.97E-11 |
| b4567 | *yjjZ* | 3667.928 | 4477.038 | 3934.904 | 52.11326 | 308.7472 | 229.9526 | 5.29E-11 |
| b3651 | *trmH* | 203.3159 | 212.0226 | 250.3897 | 53.808 | 41.31932 | 42.08953 | 5.31E-11 |
| b2942 | *metK* | 2815.284 | 2893.333 | 3520.014 | 269.8873 | 542.89 | 200.1819 | 6.20E-11 |
| b2075 | *mdtB* | 835.2435 | 851.9689 | 861.8065 | 222.4346 | 123.958 | 174.5176 | 9.86E-11 |
| b3687 | *ibpA* | 4205.524 | 4298.629 | 4838.927 | 809.662 | 1115.622 | 555.3765 | 2.66E-10 |
| b4375 | *prfC* | 2802.462 | 2421.453 | 2968.283 | 429.6166 | 748.3388 | 597.4661 | 3.43E-10 |
| b1252 | *tonB* | 947.8915 | 1004.522 | 1342.205 | 283.4453 | 297.2696 | 221.74 | 5.59E-10 |
| b0657 | *lnt* | 918.5847 | 897.2176 | 944.7845 | 270.7347 | 327.1113 | 321.3177 | 6.79E-10 |
| b3458 | *livK* | 413.0426 | 430.5093 | 499.3237 | 115.666 | 110.1849 | 71.86018 | 8.49E-10 |
| b2000 | *flu* | 937.8173 | 1179.053 | 1270.873 | 340.6427 | 241.0294 | 274.0953 | 9.29E-10 |
| b0812 | *dps* | 8151.867 | 10372.3 | 12716.01 | 1060.907 | 2266.824 | 1706.166 | 1.30E-09 |
| b0485 | *glsA* | 424.9485 | 515.8355 | 607.0495 | 88.12648 | 30.98949 | 33.87694 | 1.66E-09 |
| b3652 | *recG* | 636.5068 | 574.0124 | 746.802 | 177.9477 | 103.2983 | 145.7735 | 2.11E-09 |
| b0486 | *ybaT* | 774.7983 | 990.3007 | 1093.271 | 179.6424 | 63.12674 | 83.15249 | 2.19E-09 |
| b3932 | *hslV* | 1688.804 | 2067.22 | 2163.251 | 410.5508 | 608.3122 | 546.1374 | 2.33E-09 |
| b0848 | *ybjM* | 373.6616 | 346.476 | 413.4342 | 81.77121 | 103.2983 | 56.46157 | 2.33E-09 |
| b0847 | *ybjL* | 5032.525 | 3648.34 | 3955.284 | 657.1354 | 1166.123 | 892.0928 | 2.99E-09 |
| b0999 | *cbpM* | 837.0752 | 893.3391 | 1004.47 | 221.1636 | 97.55951 | 122.1623 | 3.14E-09 |
| b3991 | *thiG* | 282.9937 | 253.3929 | 286.7836 | 68.21329 | 33.28501 | 46.19583 | 3.56E-09 |
| b0734 | *cydB* | 10388.34 | 12506.75 | 14273.67 | 3741.139 | 2455.056 | 2226.639 | 6.17E-09 |
| b3517 | *gadA* | 84.25702 | 107.3041 | 119.3719 | 12.71055 | 2.295518 | 8.212592 | 6.20E-09 |
| b4430 | *rydB* | 1289.499 | 1606.976 | 1812.414 | 266.4979 | 80.34313 | 91.36508 | 6.43E-09 |
| b0981 | *etk* | 494.5521 | 561.0842 | 537.1733 | 193.624 | 199.7101 | 186.8365 | 1.86E-08 |
| b0695 | *kdpD* | 729.0064 | 775.6925 | 927.3155 | 277.09 | 204.3011 | 192.9959 | 1.95E-08 |
| b0879 | *macB* | 739.0806 | 742.0791 | 749.7135 | 309.2901 | 254.8025 | 280.2547 | 7.33E-08 |
| b2990 | *hybG* | 186.8308 | 210.7298 | 211.0844 | 67.36592 | 51.64915 | 50.30212 | 9.82E-08 |
| b0210 | *yafE* | 479.8987 | 562.377 | 637.6204 | 181.3372 | 177.9026 | 129.3483 | 1.00E-07 |
| b1321 | *ycjX* | 2177.861 | 2414.989 | 2506.809 | 901.178 | 719.6449 | 589.2535 | 1.03E-07 |
| b2579 | *grcA* | 40895.79 | 55577.07 | 58832.85 | 3382.277 | 11127.52 | 4594.945 | 1.13E-07 |
| b2074 | *mdtA* | 413.9584 | 394.3103 | 420.713 | 146.595 | 125.1057 | 160.1455 | 1.38E-07 |
| b0220 | *ivy* | 285.7412 | 386.5534 | 374.1289 | 112.7002 | 83.7864 | 73.91333 | 1.50E-07 |
| b0607 | *uspG* | 2968.228 | 3172.582 | 3323.487 | 594.4301 | 1112.178 | 727.8409 | 2.10E-07 |
| b4063 | *soxR* | 683.2145 | 636.0678 | 822.5012 | 179.2188 | 70.01329 | 68.78046 | 2.77E-07 |
| b1004 | *wrbA* | 651.1602 | 921.7812 | 1170.426 | 237.2636 | 126.2535 | 110.87 | 2.82E-07 |
| b2994 | *hybC* | 921.3322 | 932.1238 | 1182.072 | 368.606 | 244.4727 | 310.0253 | 3.62E-07 |
| b0020 | *nhaR* | 631.0118 | 623.1396 | 844.3375 | 146.595 | 238.7339 | 185.8099 | 3.67E-07 |
| b4290 | *fecB* | 412.1267 | 396.896 | 486.2219 | 125.4108 | 57.38795 | 59.54129 | 3.92E-07 |
| b4515 | *cydX* | 504.6263 | 544.2775 | 586.669 | 219.8925 | 159.5385 | 173.491 | 4.79E-07 |
| b1111 | *comR* | 437.7702 | 526.178 | 719.1426 | 122.8687 | 179.0504 | 107.7903 | 7.93E-07 |
| b0733 | *cydA* | 11397.59 | 13935.31 | 16503.89 | 4833.399 | 3005.981 | 2657.8 | 8.02E-07 |
| b1257 | *yciE* | 119.9747 | 143.5031 | 158.6772 | 42.3685 | 27.54621 | 25.66435 | 9.13E-07 |
| b0925 | *ldtD* | 772.0507 | 841.6263 | 1014.661 | 293.6137 | 360.3963 | 301.8127 | 1.15E-06 |
| b1226 | *narJ* | 245.4444 | 249.5144 | 321.7217 | 59.73959 | 18.36414 | 17.45176 | 1.27E-06 |
| b0735 | *ybgE* | 1060.539 | 1203.616 | 1477.591 | 273.2768 | 462.5469 | 307.9722 | 1.47E-06 |
| b3130 | *yhaV* | 561.4082 | 590.8191 | 746.802 | 173.2872 | 246.7682 | 202.2351 | 1.58E-06 |
| b3808 | *yzcX* | 282.9937 | 323.2052 | 368.3058 | 112.2765 | 60.83122 | 76.99305 | 1.68E-06 |
| b2015 | *yeeY* | 1367.345 | 1379.44 | 1627.533 | 655.4407 | 519.9348 | 535.8716 | 2.21E-06 |
| b3410 | *feoC* | 130.9647 | 192.6303 | 235.8322 | 51.68957 | 36.72829 | 44.14268 | 2.21E-06 |
| b3994 | *thiC* | 759.229 | 678.7309 | 898.2004 | 217.7741 | 299.5651 | 292.5736 | 3.06E-06 |
| b1837 | *yebW* | 350.7656 | 443.4375 | 476.0317 | 164.3898 | 156.0952 | 145.7735 | 3.50E-06 |
| b2998 | *yghW* | 306.8055 | 349.0616 | 438.182 | 75.41593 | 16.06863 | 20.53148 | 3.54E-06 |
| b0014 | *dnaK* | 62336.46 | 64297.15 | 67816.31 | 10548.91 | 23631.21 | 13184.29 | 3.58E-06 |
| b0779 | *uvrB* | 2448.033 | 2299.928 | 2988.664 | 1049.044 | 795.3969 | 648.7948 | 3.64E-06 |
| b4241 | *treR* | 236.286 | 311.5698 | 311.5314 | 108.0397 | 81.49088 | 93.41823 | 3.70E-06 |
| b4511 | *ybdZ* | 71.4353 | 89.20463 | 119.3719 | 4.660535 | 11.47759 | 18.47833 | 3.76E-06 |
| b1562 | *hokD* | 1455.265 | 2082.734 | 2074.45 | 255.0584 | 553.2198 | 495.8352 | 3.76E-06 |
| b3548 | *yhjY* | 248.1919 | 280.5421 | 323.1775 | 102.5318 | 112.4804 | 91.36508 | 4.48E-06 |
| b4492 | *ydbA* | 1400.315 | 1628.954 | 1778.932 | 719.4171 | 663.4047 | 578.9877 | 6.55E-06 |
| b2781 | *mazG* | 674.0562 | 667.0955 | 700.2178 | 327.9322 | 291.5308 | 310.0253 | 7.40E-06 |
| b4613 | *dinQ* | 478.9828 | 981.251 | 902.5677 | 39.82639 | 97.55951 | 169.3847 | 7.40E-06 |
| b4326 | *iraD* | 187.7466 | 218.4867 | 228.5534 | 60.58696 | 26.39846 | 19.50491 | 7.90E-06 |
| b3508 | *yhiD* | 363.5874 | 412.4098 | 521.16 | 86.00806 | 12.62535 | 26.69092 | 8.34E-06 |
| b1330 | *ynaI* | 530.2697 | 449.9016 | 655.0894 | 173.2872 | 92.96847 | 64.67416 | 8.87E-06 |
| b1225 | *narH* | 423.1168 | 553.3273 | 703.1293 | 156.7635 | 48.20588 | 86.23221 | 1.01E-05 |
| b1003 | *yccJ* | 348.0181 | 614.0899 | 789.0188 | 139.8161 | 165.2773 | 134.4812 | 1.05E-05 |
| b0592 | *fepB* | 598.9575 | 642.5319 | 818.1339 | 107.1923 | 230.6995 | 191.9693 | 1.07E-05 |
| b3774 | *ilvC* | 768.3874 | 978.6653 | 1033.586 | 286.4111 | 92.96847 | 120.1092 | 1.16E-05 |
| b3003 | *yghA* | 220.7168 | 241.7575 | 269.3145 | 102.5318 | 82.63864 | 71.86018 | 1.63E-05 |
| b4394 | *yjjX* | 325.1222 | 363.2826 | 410.5227 | 160.5766 | 148.0609 | 149.8798 | 1.99E-05 |
| b3993 | *thiE* | 149.2815 | 118.9395 | 179.0578 | 40.67376 | 17.21638 | 40.03639 | 2.13E-05 |
| b0150 | *fhuA* | 1711.7 | 2244.337 | 2678.588 | 163.9661 | 604.869 | 409.603 | 2.24E-05 |
| b3931 | *hslU* | 3250.306 | 3826.749 | 4285.741 | 824.9147 | 1476.018 | 1344.812 | 2.26E-05 |
| b0010 | *satP* | 273.8353 | 325.7908 | 391.5979 | 95.75281 | 138.8788 | 101.6308 | 2.46E-05 |
| b4179 | *rnr* | 3656.022 | 3950.86 | 5064.569 | 1038.876 | 1752.628 | 1449.522 | 2.48E-05 |
| b4472 | *yhdP* | 1046.802 | 1045.892 | 1276.697 | 521.5562 | 344.3277 | 374.6995 | 2.72E-05 |
| b3923 | *uspD* | 449.6761 | 521.0068 | 591.0362 | 183.4556 | 180.1982 | 96.49795 | 2.95E-05 |
| b1112 | *bhsA* | 7513.528 | 7650.913 | 8498.694 | 971.9334 | 2503.262 | 2319.031 | 3.10E-05 |
| b4221 | *tamB* | 1085.267 | 1067.87 | 1119.475 | 568.1616 | 451.0693 | 470.1709 | 3.13E-05 |
| b2475 | *ypfJ* | 685.0462 | 797.6704 | 870.5411 | 190.2346 | 309.8949 | 305.919 | 3.19E-05 |
| b0595 | *entB* | 428.6118 | 414.9955 | 589.5805 | 131.3424 | 202.0056 | 144.7469 | 3.22E-05 |
| b4721 | *ytiD* | 64.1086 | 72.39796 | 71.33196 | 4.23685 | 17.21638 | 11.29231 | 3.29E-05 |
| b0605 | *ahpC* | 28934.04 | 29518.98 | 37372.12 | 6007.853 | 12098.53 | 10615.8 | 3.32E-05 |
| b0243 | *proA* | 565.9874 | 670.974 | 759.9037 | 262.261 | 312.1904 | 263.8295 | 4.37E-05 |
| b0760 | *modF* | 340.6914 | 389.139 | 468.7529 | 177.9477 | 145.7654 | 135.5078 | 4.61E-05 |
| b3101 | *yqjF* | 217.9693 | 210.7298 | 225.6419 | 97.87124 | 68.86554 | 64.67416 | 4.65E-05 |
| b3095 | *yqjA* | 920.4164 | 992.8863 | 943.3288 | 429.1929 | 486.6498 | 363.4072 | 4.87E-05 |
| b1018 | *efeO* | 390.1466 | 359.4042 | 374.1289 | 126.2581 | 145.7654 | 182.7302 | 5.58E-05 |
| b0209 | *yafD* | 1217.148 | 1652.225 | 1723.613 | 525.3694 | 707.0195 | 552.2968 | 5.63E-05 |
| b4409 | *blr* | 172.1774 | 197.8016 | 216.9074 | 58.46853 | 59.68346 | 23.6112 | 5.68E-05 |
| b4148 | *gdx* | 428.6118 | 521.0068 | 557.5539 | 58.04485 | 164.1295 | 132.428 | 5.68E-05 |
| b4370 | *leuQ* | 22.89593 | 43.95591 | 42.21687 | 6.355275 | 3.443277 | 4.106296 | 5.81E-05 |
| b2213 | *ada* | 433.191 | 483.515 | 531.3503 | 230.061 | 195.119 | 220.7134 | 6.28E-05 |
| b1625 | *cnu* | 234.4543 | 208.1441 | 248.934 | 24.99742 | 78.04761 | 23.6112 | 6.95E-05 |
| b2993 | *hybD* | 383.7358 | 365.8683 | 505.1467 | 162.695 | 123.958 | 179.6504 | 7.24E-05 |
| b0588 | *fepC* | 291.2362 | 292.1775 | 327.5447 | 125.8344 | 68.86554 | 110.87 | 7.28E-05 |
| b1626 | *ydgK* | 860.887 | 871.3612 | 997.1917 | 181.3372 | 393.6813 | 192.9959 | 8.01E-05 |
| b1963 | *yedR* | 93.41539 | 102.1328 | 104.8143 | 39.82639 | 33.28501 | 31.82379 | 8.79E-05 |
| b0401 | *brnQ* | 925.9114 | 1035.549 | 1104.918 | 441.0561 | 502.7184 | 341.8491 | 9.46E-05 |
| b0058 | *rluA* | 379.1566 | 462.8298 | 372.6731 | 109.3107 | 153.7997 | 180.677 | 9.91E-05 |
| b3212 | *gltB* | 3058.896 | 3979.302 | 5242.171 | 1743.887 | 1230.398 | 1077.903 | 0.000106 |
| b0596 | *entA* | 351.6815 | 332.2549 | 430.9033 | 101.6844 | 168.7206 | 137.5609 | 0.00013 |
| b1661 | *cfa* | 6186.48 | 7388.471 | 9338.664 | 2697.179 | 2789.054 | 1349.945 | 0.000136 |
| b2992 | *hybE* | 192.3258 | 209.437 | 250.3897 | 86.85543 | 99.85503 | 82.12592 | 0.000136 |
| b1224 | *narG* | 889.2779 | 1005.815 | 1567.847 | 419.8718 | 149.2087 | 189.9162 | 0.000145 |
| b1785 | *cdgI* | 360.8399 | 381.3821 | 442.5493 | 146.1713 | 44.7626 | 82.12592 | 0.000145 |
| b0523 | *purE* | 142.8706 | 168.0667 | 199.4383 | 68.63697 | 43.61484 | 62.62101 | 0.000153 |
| b2469 | *narQ* | 524.7747 | 642.5319 | 736.6117 | 306.7479 | 210.0399 | 209.4211 | 0.000184 |
| b3289 | *rsmB* | 663.0661 | 819.6484 | 918.581 | 396.5692 | 317.9292 | 287.4407 | 0.000212 |
| b3167 | *rbfA* | 601.705 | 851.9689 | 975.3554 | 260.5663 | 356.953 | 313.1051 | 0.000212 |
| b0987 | *gfcA* | 144.7023 | 186.1662 | 192.1596 | 51.26589 | 72.30881 | 31.82379 | 0.000218 |
| b0790 | *ybhP* | 142.8706 | 170.6523 | 158.6772 | 68.21329 | 67.71778 | 67.75388 | 0.000226 |
| b1528 | *ydeA* | 431.3593 | 412.4098 | 406.1554 | 184.303 | 75.75209 | 108.8168 | 0.000226 |
| b1683 | *sufB* | 956.134 | 898.5104 | 1129.665 | 242.7715 | 414.341 | 431.1611 | 0.000239 |
| b0038 | *caiB* | 267.4245 | 262.4426 | 302.7969 | 118.6318 | 50.50139 | 84.17907 | 0.000255 |
| b1227 | *narI* | 147.4498 | 162.8954 | 202.3498 | 66.09486 | 26.39846 | 47.2224 | 0.000255 |
| b1563 | *relE* | 1319.721 | 1616.026 | 1824.06 | 278.7847 | 681.7688 | 456.8254 | 0.000258 |
| b0696 | *kdpC* | 143.7864 | 152.5528 | 168.8675 | 70.7554 | 41.31932 | 46.19583 | 0.000259 |
| b1597 | *asr* | 157.524 | 109.8898 | 58.23017 | 24.57373 | 6.886554 | 22.58463 | 0.000264 |
| b1322 | *ycjF* | 883.7829 | 908.853 | 873.4526 | 467.3246 | 285.792 | 290.5204 | 0.000269 |
| b3096 | *mzrA* | 250.9394 | 285.7134 | 282.4163 | 135.1555 | 91.82071 | 83.15249 | 0.000271 |
| b1129 | *phoQ* | 1455.265 | 1621.197 | 1742.538 | 821.1015 | 661.1091 | 494.8087 | 0.000296 |
| b3955 | *eptC* | 359.924 | 396.896 | 422.1687 | 194.4714 | 200.8578 | 191.9693 | 0.000299 |
| b0624 | *crcB* | 195.9892 | 241.7575 | 205.2614 | 104.6502 | 78.04761 | 73.91333 | 0.000304 |
| b0918 | *kdsB* | 702.4471 | 849.3832 | 1118.019 | 192.7767 | 397.1246 | 255.6169 | 0.000314 |
| b3515 | *gadW* | 531.1856 | 598.576 | 695.8506 | 239.8057 | 95.26399 | 70.8336 | 0.000316 |
| b3990 | *thiH* | 347.1023 | 319.3267 | 393.0537 | 132.6134 | 36.72829 | 79.0462 | 0.000328 |
| b0962 | *helD* | 726.2589 | 731.7365 | 676.9257 | 392.3323 | 303.0084 | 351.0883 | 0.000328 |
| b4140 | *fxsA* | 2086.277 | 2134.447 | 1732.348 | 966.4255 | 788.5104 | 535.8716 | 0.000328 |
| b2988 | *gss* | 1774.892 | 2043.95 | 2447.123 | 992.2703 | 483.2065 | 522.5262 | 0.000329 |
| b2527 | *hscB* | 1077.94 | 1079.505 | 1420.816 | 214.8083 | 498.1274 | 404.4701 | 0.000336 |
| b3473 | *yhhS* | 731.7539 | 876.5325 | 821.0454 | 416.906 | 415.4887 | 329.5302 | 0.000345 |
| b3267 | *yhdV* | 79.67784 | 80.15489 | 62.59743 | 27.11584 | 27.54621 | 22.58463 | 0.000373 |
| b0059 | *rapA* | 1173.187 | 1319.97 | 1398.98 | 402.5008 | 544.0377 | 670.3528 | 0.000388 |
| b3547 | *yhjX* | 415.7901 | 648.996 | 666.7355 | 199.5556 | 44.7626 | 94.44481 | 0.000389 |
| b2293 | *hxpA* | 673.1403 | 766.6427 | 876.3641 | 234.2978 | 383.3515 | 328.5037 | 0.000454 |
| b3455 | *livG* | 155.6923 | 129.2821 | 141.2082 | 61.43433 | 25.2507 | 34.90352 | 0.000494 |
| b3250 | *mreC* | 567.8191 | 590.8191 | 701.6736 | 231.7557 | 294.974 | 327.4771 | 0.000668 |
| b4024 | *lysC* | 387.3991 | 351.6472 | 499.3237 | 200.403 | 101.0028 | 114.9763 | 0.000706 |
| b2526 | *hscA* | 4309.93 | 4372.32 | 5032.543 | 896.9412 | 2175.003 | 1524.462 | 0.000709 |
| b1258 | *yciF* | 65.02444 | 106.0113 | 103.3586 | 33.8948 | 13.77311 | 22.58463 | 0.00071 |
| b2923 | *argO* | 231.7068 | 241.7575 | 254.757 | 107.1923 | 42.46708 | 82.12592 | 0.000823 |
| b2439 | *eutL* | 95.24707 | 102.1328 | 117.9161 | 46.18167 | 20.65966 | 28.74407 | 0.000848 |
| b0107 | *hofB* | 438.686 | 524.8852 | 557.5539 | 238.5347 | 88.37744 | 122.1623 | 0.000882 |
| b2134 | *pbpG* | 195.0733 | 266.3211 | 257.6685 | 85.16069 | 104.4461 | 118.056 | 0.00089 |
| b1481 | *bdm* | 41.21267 | 74.9836 | 59.68593 | 10.16844 | 19.5119 | 17.45176 | 0.000974 |
| b0868 | *ybjS* | 247.276 | 283.1277 | 311.5314 | 147.4424 | 122.8102 | 100.6042 | 0.001019 |
| b1178 | *pliG* | 204.2317 | 259.857 | 273.6818 | 91.09228 | 122.8102 | 64.67416 | 0.001071 |
| b3171 | *metY* | 368.1665 | 387.8462 | 497.868 | 105.4976 | 200.8578 | 74.9399 | 0.001125 |
| b0594 | *entE* | 684.1304 | 712.3442 | 957.8863 | 128.8002 | 322.5203 | 290.5204 | 0.001172 |
| b1797 | *yeaR* | 89.75204 | 99.5472 | 126.6506 | 44.06324 | 17.21638 | 16.42518 | 0.001243 |
| b1923 | *fliC* | 853.5603 | 1065.284 | 1233.024 | 388.0955 | 575.0272 | 375.7261 | 0.001319 |
| b0417 | *thiL* | 402.0525 | 329.6693 | 409.067 | 210.1478 | 161.834 | 142.6938 | 0.001319 |
| b2671 | *ygaC* | 236.286 | 226.2436 | 211.0844 | 115.666 | 59.68346 | 84.17907 | 0.001347 |
| b0660 | *ybeZ* | 2174.197 | 2540.393 | 2572.318 | 649.9328 | 1224.659 | 1110.753 | 0.001566 |
| b3409 | *feoB* | 3329.068 | 4396.883 | 5016.529 | 1962.085 | 579.6183 | 826.392 | 0.001638 |
| b2683 | *ygaH* | 108.9846 | 175.8236 | 170.3233 | 42.3685 | 65.42226 | 61.59444 | 0.001643 |
| b2449 | *yffR* | 149.2815 | 193.9231 | 186.3365 | 90.66859 | 56.24019 | 51.3287 | 0.001652 |
| b3685 | *yidE* | 565.9874 | 474.4652 | 391.5979 | 256.7531 | 154.9475 | 172.4644 | 0.00169 |
| b3100 | *yqjK* | 374.5774 | 509.3714 | 566.2884 | 55.50274 | 206.5966 | 118.056 | 0.001697 |
| b1716 | *rplT* | 17194.84 | 20814.41 | 22296.33 | 3373.38 | 9229.13 | 7135.716 | 0.001724 |
| b3922 | *yiiS* | 390.1466 | 394.3103 | 492.045 | 218.1978 | 110.1849 | 91.36508 | 0.001749 |
| b2928 | *yggC* | 352.5973 | 329.6693 | 323.1775 | 186.8451 | 102.1505 | 109.8434 | 0.001811 |
| b0458 | *ylaC* | 500.0471 | 536.5206 | 596.8593 | 169.0503 | 305.3039 | 223.7931 | 0.001878 |
| b2214 | *ftp* | 312.3005 | 359.4042 | 423.6245 | 197.4372 | 173.3116 | 170.4113 | 0.001893 |
| b0192 | *nlpE* | 703.363 | 1010.986 | 970.9881 | 461.8167 | 445.3305 | 429.1079 | 0.001945 |
| b1717 | *rpmI* | 14111.22 | 17325.09 | 19102.41 | 2900.971 | 7885.104 | 5972.607 | 0.002017 |
| b1711 | *btuC* | 232.6226 | 232.7077 | 304.2526 | 135.5792 | 78.04761 | 71.86018 | 0.002039 |
| b3385 | *gph* | 206.9792 | 270.1995 | 340.6465 | 121.1739 | 129.6968 | 103.684 | 0.002055 |
| b4064 | *ghxP* | 453.3394 | 479.6365 | 490.5892 | 197.4372 | 36.72829 | 80.07277 | 0.002055 |
| b0186 | *ldcC* | 392.8942 | 414.9955 | 496.4122 | 239.382 | 130.8445 | 168.3581 | 0.002065 |
| b2565 | *recO* | 221.6326 | 248.2216 | 248.934 | 132.1897 | 125.1057 | 114.9763 | 0.002093 |
| b0242 | *proB* | 958.8815 | 1171.296 | 1324.736 | 442.7508 | 661.1091 | 418.8422 | 0.002095 |
| b2155 | *cirA* | 1174.103 | 1334.191 | 1423.728 | 276.2426 | 610.6078 | 572.8283 | 0.002116 |
| b2076 | *mdtC* | 464.3295 | 483.515 | 423.6245 | 229.2136 | 151.5042 | 245.3512 | 0.002141 |
| b0019 | *nhaA* | 1298.657 | 1459.595 | 1776.02 | 342.7612 | 808.0223 | 430.1345 | 0.002155 |
| b3992 | *thiF* | 218.8851 | 148.6744 | 203.8056 | 69.90803 | 12.62535 | 43.11611 | 0.00221 |
| b4295 | *yjhU* | 1011.084 | 1035.549 | 1298.533 | 584.6853 | 638.154 | 508.1541 | 0.002236 |
| b4452 | *gadY* | 101.6579 | 108.5969 | 129.5621 | 24.57373 | 45.91036 | 50.30212 | 0.002238 |
| b2737 | *ygbK* | 224.3801 | 201.68 | 183.425 | 85.16069 | 20.65966 | 54.40842 | 0.002364 |
| b2063 | *yegH* | 551.334 | 521.0068 | 556.0981 | 317.3401 | 176.7549 | 208.3945 | 0.002375 |
| b4167 | *nnr* | 689.6254 | 691.6591 | 775.917 | 441.0561 | 360.3963 | 340.8226 | 0.002383 |
| b3714 | *adeP* | 563.2399 | 615.3827 | 684.2045 | 342.3375 | 359.2485 | 290.5204 | 0.002415 |
| b2438 | *eutK* | 111.7321 | 94.37592 | 126.6506 | 53.38431 | 49.35363 | 44.14268 | 0.00251 |
| b3812 | *yigB* | 408.4634 | 402.0673 | 442.5493 | 234.7215 | 179.0504 | 234.0589 | 0.00251 |
| b0183 | *rnhB* | 294.8996 | 367.1611 | 413.4342 | 138.9687 | 180.1982 | 189.9162 | 0.002883 |
| b0188 | *tilS* | 378.2408 | 413.7026 | 292.6066 | 185.1503 | 87.22968 | 141.6672 | 0.003041 |
| b1678 | *ldtE* | 999.1784 | 1157.075 | 1461.577 | 321.1532 | 651.9271 | 464.0114 | 0.003054 |
| b2440 | *eutC* | 225.2959 | 239.1718 | 262.0358 | 126.6818 | 48.20588 | 68.78046 | 0.003065 |
| b0324 | *yahJ* | 386.4833 | 409.8242 | 497.868 | 244.0426 | 128.549 | 131.4015 | 0.003144 |
| b0951 | *pqiB* | 518.3638 | 526.178 | 604.138 | 244.8899 | 330.5546 | 290.5204 | 0.003295 |
| b4320 | *fimH* | 166.6824 | 208.1441 | 237.2879 | 105.9213 | 95.26399 | 74.9399 | 0.003295 |
| b2473 | *ypfH* | 496.3838 | 542.9847 | 542.9964 | 289.3769 | 129.6968 | 208.3945 | 0.003456 |
| b1198 | *dhaM* | 274.7512 | 314.1554 | 339.1907 | 163.1187 | 158.3907 | 176.5707 | 0.003472 |
| b3681 | *glvG* | 98.91042 | 124.1108 | 138.2967 | 59.73959 | 26.39846 | 39.00981 | 0.003691 |
| b3550 | *yiaC* | 171.2616 | 199.0944 | 232.9207 | 100.4133 | 104.4461 | 72.88675 | 0.003827 |
| b3099 | *yqjE* | 809.6001 | 1062.699 | 1139.856 | 219.0451 | 537.1512 | 349.0352 | 0.00384 |
| b3053 | *glnE* | 616.3584 | 638.6535 | 777.3728 | 382.5876 | 381.056 | 371.6198 | 0.003844 |
| b3844 | *fre* | 372.7457 | 424.0452 | 487.6777 | 242.3478 | 212.3354 | 232.0057 | 0.003915 |
| b3937 | *yiiX* | 326.038 | 289.5918 | 449.8281 | 167.3556 | 185.9369 | 152.9595 | 0.003965 |
| b3497 | *rsmJ* | 206.0634 | 193.9231 | 167.4117 | 97.87124 | 55.09243 | 89.31194 | 0.004034 |
| b2097 | *fbaB* | 546.7548 | 696.8304 | 790.4746 | 216.503 | 382.2037 | 289.4939 | 0.004034 |
| b0836 | *bssR* | 2464.518 | 4577.878 | 4662.781 | 1962.933 | 747.1911 | 1031.707 | 0.004543 |
| b3743 | *asnC* | 151.1131 | 171.9452 | 163.0445 | 88.97385 | 79.19537 | 57.48814 | 0.004646 |
| b3249 | *mreD* | 155.6923 | 160.3098 | 149.9427 | 87.27911 | 80.34313 | 74.9399 | 0.00485 |
| b1259 | *yciG* | 50.37105 | 100.84 | 107.7258 | 19.06583 | 39.0238 | 19.50491 | 0.004964 |
| b1796 | *yoaG* | 33.88598 | 28.44206 | 45.12838 | 13.55792 | 5.738795 | 8.212592 | 0.004997 |
| b2519 | *pbpC* | 502.7946 | 533.935 | 569.1999 | 286.8347 | 92.96847 | 161.1721 | 0.004997 |
| b3675 | *yidG* | 109.9005 | 164.1882 | 128.1064 | 27.53953 | 70.01329 | 40.03639 | 0.005103 |
| b3343 | *tusB* | 902.0996 | 976.0797 | 847.249 | 479.6114 | 505.0139 | 289.4939 | 0.005395 |
| b1739 | *osmE* | 1039.475 | 1829.341 | 1554.746 | 133.0371 | 635.8584 | 385.9918 | 0.005436 |
| b4377 | *yjjU* | 142.8706 | 186.1662 | 224.1862 | 78.38173 | 78.04761 | 98.5511 | 0.005546 |
| b4079 | *fdhF* | 435.0227 | 526.178 | 577.9345 | 264.3794 | 122.8102 | 235.0854 | 0.005664 |
| b1292 | *sapC* | 142.8706 | 124.1108 | 129.5621 | 75.41593 | 48.20588 | 55.43499 | 0.00572 |
| b0522 | *purK* | 229.8751 | 230.1221 | 218.3631 | 132.1897 | 67.71778 | 92.39166 | 0.005737 |
| b0124 | *gcd* | 717.1005 | 664.5099 | 694.3948 | 440.2087 | 413.1932 | 395.231 | 0.005737 |
| b3469 | *zntA* | 615.4426 | 563.6699 | 518.2485 | 266.4979 | 353.5098 | 316.1848 | 0.006414 |
| b4690 | *eyeA* | 68.68779 | 74.9836 | 88.80101 | 36.8606 | 33.28501 | 34.90352 | 0.006527 |
| b4539 | *yoeB* | 437.7702 | 517.1283 | 617.2398 | 193.624 | 236.4383 | 314.1316 | 0.006654 |
| b0173 | *dxr* | 388.315 | 405.9457 | 496.4122 | 252.5163 | 242.1771 | 212.5008 | 0.006677 |
| b1709 | *btuD* | 182.2516 | 200.3872 | 234.3764 | 105.0739 | 115.9237 | 108.8168 | 0.006966 |
| b1687 | *ydiJ* | 823.3376 | 880.4109 | 1013.205 | 486.3904 | 581.9138 | 457.852 | 0.007079 |
| b2621 | *ssrA* | 3241091 | 5010739 | 5228349 | 558453.3 | 1193318 | 2241667 | 0.007154 |
| b3987 | *rpoB* | 17865.24 | 20779.51 | 28222.71 | 3619.965 | 11285.91 | 7907.699 | 0.007453 |
| b0153 | *fhuB* | 1180.514 | 1185.517 | 1052.51 | 557.5695 | 129.6968 | 351.0883 | 0.00752 |
| b0583 | *entD* | 151.1131 | 218.4867 | 186.3365 | 45.3343 | 97.55951 | 80.07277 | 0.007802 |
| b3693 | *dgoK* | 75.09865 | 99.5472 | 66.9647 | 36.43691 | 34.43277 | 36.95666 | 0.007802 |
| b0258 | *ykfC* | 273.8353 | 294.7631 | 302.7969 | 181.3372 | 115.9237 | 141.6672 | 0.007802 |
| b3248 | *yhdE* | 208.8109 | 204.2657 | 296.9739 | 97.87124 | 137.7311 | 99.57768 | 0.007827 |
| b2688 | *gshA* | 1864.645 | 3056.228 | 2797.96 | 541.8931 | 1344.026 | 1030.68 | 0.007861 |
| b2753 | *iap* | 441.4335 | 499.0288 | 439.6378 | 266.0742 | 89.5252 | 117.0294 | 0.007897 |
| b3430 | *glgC* | 1989.198 | 2109.883 | 2543.203 | 572.3984 | 1264.83 | 1005.016 | 0.008251 |
| b2841 | *araE* | 412.1267 | 483.515 | 401.7882 | 273.2768 | 198.5623 | 167.3316 | 0.008253 |
| b0247 | *ykfG* | 21.98009 | 33.61334 | 32.02659 | 10.59213 | 2.295518 | 5.13287 | 0.008253 |
| b3967 | *murI* | 533.9331 | 555.9129 | 559.0096 | 337.2533 | 280.0532 | 190.9428 | 0.008341 |
| b1635 | *gstA* | 873.7087 | 881.7038 | 1083.081 | 235.9925 | 549.7765 | 399.3373 | 0.008426 |
| b2747 | *ispD* | 209.7267 | 190.0447 | 228.5534 | 124.5634 | 113.6281 | 106.7637 | 0.008499 |
| b1981 | *shiA* | 568.7349 | 658.0458 | 589.5805 | 375.8086 | 363.8396 | 289.4939 | 0.008733 |
| b1653 | *lhr* | 1131.975 | 1052.356 | 1049.599 | 682.5565 | 371.8739 | 520.473 | 0.00874 |
| b0414 | *ribD* | 942.3965 | 1025.207 | 1455.754 | 457.1561 | 678.3255 | 535.8716 | 0.009303 |
| b2539 | *hcaF* | 71.4353 | 56.88411 | 119.3719 | 33.8948 | 29.84173 | 31.82379 | 0.009897 |
| b0902 | *pflA* | 1076.109 | 946.3448 | 1085.993 | 575.7879 | 697.8374 | 557.4297 | 0.010025 |
| b1956 | *dgcQ* | 359.0082 | 377.5037 | 451.2838 | 227.5188 | 243.3249 | 195.0491 | 0.010132 |
| b0090 | *murG* | 443.2652 | 527.4709 | 564.8327 | 273.2768 | 328.2591 | 262.8029 | 0.010161 |
| b3960 | *argH* | 315.048 | 321.9124 | 278.0491 | 176.253 | 191.6757 | 161.1721 | 0.010208 |
| b0482 | *ybaP* | 422.2009 | 420.1667 | 452.7396 | 264.3794 | 250.2114 | 176.5707 | 0.010223 |
| b3772 | *ilvA* | 348.0181 | 341.3047 | 365.3943 | 231.7557 | 175.6071 | 164.2518 | 0.010223 |
| b0378 | *yaiW* | 297.6471 | 358.1113 | 362.4828 | 199.9793 | 137.7311 | 194.0225 | 0.010358 |
| b1007 | *rutF* | 73.26697 | 56.88411 | 32.02659 | 19.06583 | 2.295518 | 12.31889 | 0.01069 |
| b0148 | *hrpB* | 664.8978 | 619.2611 | 751.1692 | 433.8534 | 405.1589 | 382.9121 | 0.010887 |
| b0473 | *htpG* | 2875.729 | 3272.129 | 3690.337 | 619.0038 | 1822.641 | 1320.174 | 0.011201 |
| b1978 | *yeeJ* | 903.0155 | 950.2233 | 972.4439 | 592.7353 | 243.3249 | 356.2212 | 0.011405 |
| b1564 | *relB* | 736.3331 | 762.7642 | 918.581 | 208.453 | 482.0588 | 355.1946 | 0.011611 |
| b1629 | *rsxC* | 284.8254 | 222.3652 | 339.1907 | 160.1529 | 114.7759 | 148.8532 | 0.012039 |
| b3352 | *yheS* | 675.8878 | 655.4601 | 783.1958 | 412.2455 | 449.9215 | 432.1876 | 0.012274 |
| b4220 | *tamA* | 607.2001 | 651.5817 | 730.7887 | 408.0087 | 407.4544 | 410.6296 | 0.012447 |
| b3407 | *yhgF* | 506.458 | 521.0068 | 575.0229 | 332.169 | 238.7339 | 325.424 | 0.012509 |
| b2715 | *ascF* | 202.4 | 221.0723 | 292.6066 | 142.3582 | 115.9237 | 102.6574 | 0.012745 |
| b2016 | *yeeZ* | 1997.441 | 2146.082 | 2738.274 | 960.0702 | 1462.245 | 1212.384 | 0.013131 |
| b0069 | *sgrR* | 452.4236 | 466.7083 | 559.0096 | 325.8138 | 241.0294 | 221.74 | 0.013196 |
| b2520 | *yfhM* | 1708.952 | 1980.601 | 2335.03 | 1147.763 | 1233.841 | 1195.959 | 0.013196 |
| b2218 | *rcsC* | 671.3087 | 758.8858 | 740.9789 | 460.1219 | 474.0244 | 418.8422 | 0.013663 |
| b2814 | *metZ* | 72.35114 | 90.49745 | 82.97799 | 41.09745 | 11.47759 | 28.74407 | 0.014188 |
| b2459 | *eutT* | 77.84616 | 68.5195 | 46.58414 | 33.04743 | 21.80742 | 15.39861 | 0.014609 |
| b0377 | *sbmA* | 415.7901 | 396.896 | 436.7263 | 235.1452 | 208.8921 | 282.3078 | 0.014716 |
| b1019 | *efeB* | 382.8199 | 356.8185 | 391.5979 | 218.1978 | 86.08192 | 174.5176 | 0.015214 |
| b1280 | *lapB* | 1299.573 | 1671.617 | 1758.551 | 677.4723 | 1000.846 | 887.9865 | 0.015583 |
| b0806 | *mcbA* | 119.9747 | 138.3318 | 205.2614 | 55.07905 | 90.67296 | 59.54129 | 0.016105 |
| b3523 | *yhjE* | 369.0824 | 426.6308 | 326.089 | 214.3846 | 61.97898 | 132.428 | 0.016577 |
| b0597 | *entH* | 162.1032 | 140.9175 | 145.5754 | 55.92642 | 88.37744 | 84.17907 | 0.017143 |
| b1834 | *yebT* | 510.1213 | 517.1283 | 545.9079 | 329.6269 | 322.5203 | 351.0883 | 0.017382 |
| b2943 | *galP* | 242.6969 | 235.2934 | 278.0491 | 158.4582 | 92.96847 | 61.59444 | 0.017678 |
| b4119 | *melA* | 167.5982 | 159.017 | 189.2481 | 108.887 | 80.34313 | 91.36508 | 0.01776 |
| b1999 | *yeeP* | 96.1629 | 67.22668 | 109.1816 | 49.57115 | 40.17156 | 33.87694 | 0.018284 |
| b2441 | *eutB* | 234.4543 | 285.7134 | 302.7969 | 161.0003 | 168.7206 | 151.9329 | 0.01914 |
| b3729 | *glmS* | 727.1747 | 903.6817 | 965.1651 | 364.7928 | 527.9691 | 528.6856 | 0.019358 |
| b2470 | *acrD* | 554.0815 | 597.2832 | 627.4301 | 354.2007 | 111.3326 | 236.112 | 0.019428 |
| b4699 | *fnrS* | 53.11856 | 50.42001 | 61.14168 | 24.15005 | 30.98949 | 16.42518 | 0.020824 |
| b2991 | *hybF* | 123.638 | 95.66874 | 120.8276 | 55.07905 | 26.39846 | 62.62101 | 0.021304 |
| b0732 | *mngB* | 504.6263 | 562.377 | 690.0275 | 364.7928 | 109.0371 | 187.863 | 0.021424 |
| b4371 | *rsmC* | 453.3394 | 595.9904 | 745.3462 | 172.4398 | 361.5441 | 280.2547 | 0.021769 |
| b0167 | *glnD* | 1689.72 | 1596.634 | 2081.729 | 888.4675 | 1238.432 | 968.0593 | 0.022468 |
| b1681 | *sufD* | 1075.193 | 894.632 | 1286.887 | 153.7977 | 476.32 | 557.4297 | 0.022573 |
| b3706 | *mnmE* | 309.553 | 338.719 | 390.1421 | 237.2636 | 143.4699 | 156.0392 | 0.022763 |
| b3166 | *truB* | 659.4028 | 638.6535 | 826.8684 | 351.6586 | 474.0244 | 411.6562 | 0.023164 |
| b3428 | *glgP* | 1986.451 | 2371.033 | 2930.433 | 616.8854 | 1465.688 | 1177.48 | 0.023395 |
| b2388 | *glk* | 2069.792 | 1767.286 | 2013.308 | 1379.095 | 899.843 | 740.1598 | 0.023453 |
| b1447 | *ydcZ* | 164.8507 | 201.68 | 192.1596 | 120.3265 | 101.0028 | 97.52453 | 0.023908 |
| b0284 | *paoC* | 351.6815 | 277.9565 | 279.5048 | 180.9135 | 50.50139 | 109.8434 | 0.023908 |
| b0184 | *dnaE* | 1594.473 | 1741.43 | 2332.118 | 728.3145 | 1212.033 | 1020.415 | 0.023933 |
| b0015 | *dnaJ* | 2439.79 | 2599.863 | 2789.225 | 862.199 | 1691.797 | 1445.416 | 0.023933 |
| b4289 | *fecC* | 284.8254 | 236.5862 | 176.1463 | 91.51596 | 4.591036 | 49.27555 | 0.02397 |
| b3282 | *tsaC* | 250.9394 | 250.8072 | 259.1243 | 160.5766 | 133.14 | 166.305 | 0.024361 |
| b3641 | *slmA* | 185.9149 | 195.2159 | 190.7038 | 82.19489 | 112.4804 | 123.1889 | 0.024361 |
| b4580 | *yaiT* | 262.8453 | 294.7631 | 321.7217 | 187.6925 | 72.30881 | 123.1889 | 0.024622 |
| b3398 | *igaA* | 740.9123 | 989.0079 | 1001.559 | 223.282 | 552.072 | 440.4002 | 0.024677 |
| b2464 | *talA* | 664.8978 | 921.7812 | 1037.953 | 266.4979 | 569.2884 | 379.8324 | 0.024731 |
| b2451 | *eutA* | 259.1819 | 231.4149 | 147.0312 | 109.7344 | 20.65966 | 66.72731 | 0.025133 |
| b2784 | *relA* | 660.3186 | 705.8801 | 850.1605 | 429.6166 | 452.217 | 490.7024 | 0.025475 |
| b0106 | *hofC* | 280.2462 | 292.1775 | 299.8854 | 189.8109 | 70.01329 | 117.0294 | 0.025639 |
| b0829 | *gsiA* | 357.1765 | 444.7303 | 563.3769 | 277.5137 | 167.5728 | 246.3778 | 0.025725 |
| b2782 | *mazF* | 250.0236 | 261.1498 | 310.0757 | 78.38173 | 179.0504 | 125.242 | 0.025776 |
| b4028 | *yjbG* | 96.1629 | 78.86207 | 46.58414 | 36.01323 | 4.591036 | 13.34546 | 0.025862 |
| b1215 | *kdsA* | 1511.131 | 1641.882 | 1805.135 | 582.1432 | 1100.701 | 910.5711 | 0.025967 |
| b3098 | *yqjD* | 883.7829 | 1150.61 | 1265.05 | 255.4821 | 700.1329 | 459.9051 | 0.02607 |
| b2831 | *mutH* | 336.1122 | 431.8021 | 435.2705 | 188.5398 | 263.9846 | 235.0854 | 0.026355 |
| b3449 | *ugpQ* | 238.1177 | 250.8072 | 181.9693 | 143.6292 | 101.0028 | 121.1357 | 0.026619 |
| b0830 | *gsiB* | 293.0679 | 308.9842 | 397.4209 | 218.6215 | 146.9131 | 181.7036 | 0.026946 |
| b3882 | *yihU* | 134.6281 | 107.3041 | 88.80101 | 61.43433 | 10.32983 | 22.58463 | 0.026986 |
| b4361 | *dnaC* | 301.3104 | 311.5698 | 311.5314 | 188.1161 | 196.2668 | 207.3679 | 0.02725 |
| b3530 | *bcsC* | 552.2498 | 526.178 | 726.4214 | 401.6534 | 156.0952 | 234.0589 | 0.028706 |
| b2732 | *ygbA* | 179.5041 | 148.6744 | 197.9826 | 102.1081 | 109.0371 | 94.44481 | 0.029076 |
| b2913 | *serA* | 1042.223 | 1438.91 | 1726.525 | 321.1532 | 863.1147 | 612.8647 | 0.029076 |
| b3103 | *yhaH* | 119.9747 | 146.0887 | 141.2082 | 83.88963 | 75.75209 | 50.30212 | 0.029915 |
| b2488 | *hyfH* | 57.69774 | 62.0554 | 65.50894 | 35.16586 | 8.034313 | 17.45176 | 0.031032 |
| b0400 | *phoR* | 247.276 | 217.1939 | 272.2261 | 163.5424 | 144.6176 | 129.3483 | 0.031109 |
| b3791 | *wecE* | 224.3801 | 281.8349 | 238.7437 | 155.4924 | 145.7654 | 155.0127 | 0.031589 |
| b0487 | *cueR* | 679.5512 | 762.7642 | 1021.94 | 176.6766 | 531.4124 | 320.2911 | 0.031962 |
| b0714 | *nei* | 258.2661 | 236.5862 | 298.4296 | 167.7793 | 45.91036 | 92.39166 | 0.032018 |
| b0068 | *thiB* | 274.7512 | 297.3488 | 334.8235 | 169.8977 | 203.1533 | 187.863 | 0.032233 |
| b4180 | *rlmB* | 362.6715 | 373.6252 | 524.0715 | 151.6792 | 274.3144 | 218.6603 | 0.032258 |
| b2077 | *mdtD* | 211.5584 | 183.5805 | 186.3365 | 136.0029 | 79.19537 | 81.09934 | 0.032371 |
| b2184 | *radD* | 318.7113 | 365.8683 | 388.6864 | 230.4846 | 145.7654 | 221.74 | 0.033378 |
| b1871 | *cmoB* | 165.7665 | 146.0887 | 171.779 | 107.616 | 53.94467 | 78.01962 | 0.033574 |
| b2496 | *hda* | 252.7711 | 231.4149 | 221.2747 | 161.8477 | 137.7311 | 119.0826 | 0.033587 |
| b3252 | *csrD* | 1138.386 | 1201.03 | 1390.245 | 610.9538 | 903.2863 | 700.1235 | 0.03368 |
| b3281 | *aroE* | 162.1032 | 195.2159 | 199.4383 | 119.9029 | 98.70727 | 62.62101 | 0.033807 |
| b2482 | *hyfB* | 278.4145 | 227.5365 | 173.2348 | 136.4266 | 52.79691 | 104.7105 | 0.033909 |
| b0964 | *yccT* | 182.2516 | 138.3318 | 163.0445 | 102.5318 | 90.67296 | 88.28536 | 0.034057 |
| b4065 | *yjcE* | 398.3892 | 489.9791 | 387.2306 | 294.0374 | 205.4488 | 245.3512 | 0.03432 |
| b1786 | *dgcJ* | 404.8 | 404.6529 | 563.3769 | 292.3427 | 275.4621 | 219.6868 | 0.034487 |
| b3479 | *nikD* | 359.0082 | 409.8242 | 251.8455 | 191.0819 | 61.97898 | 161.1721 | 0.035824 |
| b3290 | *trkA* | 413.9584 | 381.3821 | 353.7483 | 257.6005 | 114.7759 | 194.0225 | 0.035878 |
| b0752 | *zitB* | 219.8009 | 262.4426 | 269.3145 | 158.8819 | 159.5385 | 149.8798 | 0.036299 |
| b1812 | *pabB* | 141.9548 | 162.8954 | 167.4117 | 99.98966 | 70.01329 | 96.49795 | 0.036462 |
| b3457 | *livH* | 121.8063 | 139.6246 | 141.2082 | 85.58437 | 20.65966 | 37.98324 | 0.036534 |
| b4486 | *yjiV* | 550.4181 | 562.377 | 640.5319 | 427.9219 | 207.7444 | 246.3778 | 0.037207 |
| b0067 | *thiP* | 316.8797 | 250.8072 | 250.3897 | 150.4082 | 41.31932 | 129.3483 | 0.037428 |
| b3988 | *rpoC* | 17849.67 | 20930.77 | 26488.91 | 3416.596 | 13185.45 | 9042.064 | 0.038507 |
| b2389 | *yfeO* | 384.6516 | 436.9734 | 476.0317 | 312.6795 | 176.7549 | 214.554 | 0.038761 |
| b4367 | *fhuF* | 2264.865 | 2831.277 | 3400.642 | 81.77121 | 1311.888 | 675.4857 | 0.038852 |
| b0789 | *clsB* | 185.9149 | 180.9949 | 176.1463 | 123.2923 | 39.0238 | 62.62101 | 0.039345 |
| b2809 | *ygdI* | 652.9919 | 1058.82 | 1024.851 | 105.4976 | 522.2303 | 337.7428 | 0.040197 |
| b2997 | *hybO* | 1794.125 | 2028.436 | 2372.879 | 1521.877 | 670.2912 | 751.4521 | 0.040315 |
| b0832 | *gsiD* | 166.6824 | 254.6857 | 195.0711 | 116.5134 | 20.65966 | 69.80703 | 0.040315 |
| b1657 | *ydhP* | 743.6598 | 815.7699 | 908.3907 | 614.3433 | 387.9425 | 383.9387 | 0.040315 |
| b3247 | *rng* | 500.9629 | 607.6258 | 611.4168 | 286.8347 | 418.932 | 322.3442 | 0.040767 |
| b3129 | *prlF* | 292.1521 | 270.1995 | 345.0138 | 126.6818 | 220.3697 | 145.7735 | 0.040837 |
| b1560 | *ydfU* | 183.1674 | 148.6744 | 144.1197 | 111.4292 | 47.05812 | 53.38185 | 0.042274 |
| b0095 | *ftsZ* | 1314.226 | 1687.131 | 2154.516 | 430.0403 | 1093.814 | 843.8438 | 0.04252 |
| b4346 | *mcrB* | 139.2073 | 125.4036 | 149.9427 | 95.75281 | 63.12674 | 48.24898 | 0.042773 |
| b0096 | *lpxC* | 26034.5 | 37387.08 | 39356.32 | 5131.673 | 21700.68 | 12756.21 | 0.043167 |
| b1684 | *sufA* | 330.6172 | 329.6693 | 454.1953 | 58.04485 | 189.3802 | 199.1554 | 0.043168 |
| b2995 | *hybB* | 679.5512 | 744.6648 | 812.3109 | 541.4694 | 190.528 | 267.9358 | 0.043286 |
| b1972 | *msrQ* | 170.3457 | 215.9011 | 235.8322 | 146.595 | 67.71778 | 60.56786 | 0.043307 |
| b2582 | *trxC* | 628.2643 | 519.7139 | 547.3636 | 83.04226 | 364.9873 | 206.3414 | 0.043521 |
| b0475 | *hemH* | 226.2118 | 208.1441 | 224.1862 | 131.766 | 156.0952 | 104.7105 | 0.044098 |
| b0998 | *torD* | 91.58372 | 107.3041 | 161.5887 | 63.55275 | 70.01329 | 45.16925 | 0.044251 |
| b2577 | *yfiE* | 723.5114 | 776.9853 | 774.4613 | 577.9063 | 410.8977 | 369.5666 | 0.046859 |
| b0546 | *ybcM* | 182.2516 | 186.1662 | 196.5268 | 135.1555 | 103.2983 | 97.52453 | 0.046897 |
| b0439 | *lon* | 4695.497 | 5446.654 | 5901.628 | 1504.082 | 3825.481 | 2502.787 | 0.046897 |
| b3030 | *parE* | 924.9956 | 970.9084 | 1142.767 | 637.2222 | 723.0881 | 650.8479 | 0.047503 |
| b1884 | *cheR* | 105.3213 | 112.4754 | 119.3719 | 79.65278 | 39.0238 | 42.08953 | 0.048116 |
| b4481 | *rffT* | 309.553 | 297.3488 | 305.7084 | 205.9109 | 164.1295 | 211.4742 | 0.048627 |

Table S2. Differential gene expression genes (down-regulated) of *E. coli* W3110 under gallic acid stress versus fermentation without stress.

| Gene  id | Gene  name | Treatment-1 | Treatment-2 | Treatment-3 | Control-1 | Control-2 | Control-3 | padj |
| --- | --- | --- | --- | --- | --- | --- | --- | --- |
| b2096 | *gatY* | 459.7503 | 305.1057 | 369.7616 | 7355.172 | 6500.907 | 7321.526 | 2.74E-47 |
| b2181 | *yejG* | 152.9448 | 180.9949 | 195.0711 | 3273.814 | 5422.013 | 4600.078 | 5.55E-43 |
| b4139 | *aspA* | 223.4643 | 319.3267 | 314.4429 | 5701.529 | 4368.371 | 4557.988 | 1.65E-39 |
| b2094 | *gatA* | 52.20272 | 36.19898 | 37.84961 | 2206.128 | 1221.216 | 1344.812 | 1.27E-35 |
| b2095 | *gatZ* | 282.0779 | 223.658 | 282.4163 | 9159.646 | 4790.746 | 5796.037 | 2.64E-34 |
| b2093 | *gatB* | 31.13846 | 25.85642 | 21.83631 | 1011.76 | 577.3227 | 649.8213 | 1.98E-29 |
| b2092 | *gatC* | 180.4199 | 199.0944 | 144.1197 | 4285.998 | 2411.442 | 2832.318 | 3.36E-29 |
| b1892 | *flhD* | 126.3855 | 109.8898 | 106.2701 | 1435.021 | 1633.261 | 1040.946 | 3.15E-27 |
| b3195 | *mlaF* | 203.3159 | 200.3872 | 163.0445 | 1663.811 | 3210.282 | 2694.757 | 1.44E-22 |
| b3261 | *fis* | 45.79186 | 62.0554 | 81.52224 | 755.8541 | 1384.197 | 1091.248 | 5.07E-22 |
| b4443 | *gcvB* | 102.5738 | 180.9949 | 180.5135 | 2034.959 | 4192.763 | 5303.281 | 2.30E-20 |
| b2288 | *nuoA* | 201.4842 | 250.8072 | 234.3764 | 1960.391 | 3909.267 | 2323.137 | 3.34E-19 |
| b2597 | *raiA* | 394.7258 | 318.0339 | 406.1554 | 3288.643 | 7551.106 | 4797.18 | 3.61E-19 |
| b3260 | *dusB* | 127.3014 | 204.2657 | 254.757 | 2038.772 | 3741.694 | 2946.267 | 4.53E-19 |
| b1205 | *ychH* | 204.2317 | 292.1775 | 264.9473 | 2055.296 | 4602.513 | 3663.843 | 2.41E-18 |
| b0211 | *mltD* | 131.8806 | 140.9175 | 173.2348 | 2163.336 | 12105.41 | 6949.906 | 3.56E-18 |
| b2878 | *ygfK* | 163.019 | 155.1385 | 117.9161 | 1281.647 | 1313.036 | 2306.712 | 4.92E-18 |
| b2877 | *mocA* | 40.29684 | 40.07744 | 36.39386 | 259.2952 | 266.2801 | 306.9456 | 7.09E-18 |
| b1858 | *znuC* | 264.6769 | 250.8072 | 349.381 | 1807.864 | 2956.627 | 2234.852 | 1.42E-17 |
| b1857 | *znuA* | 292.1521 | 307.6913 | 308.6199 | 1851.503 | 2661.653 | 1659.97 | 2.51E-17 |
| b4015 | *aceA* | 3455.454 | 4077.557 | 5834.663 | 34058.77 | 67873.87 | 68957.03 | 8.88E-17 |
| b2870 | *ygeW* | 46.7077 | 53.00565 | 32.02659 | 629.1722 | 890.6609 | 2246.144 | 1.57E-15 |
| b0023 | *rpsT* | 101.6579 | 117.6467 | 131.0179 | 1288.426 | 7725.565 | 4497.421 | 1.68E-15 |
| b2285 | *nuoE* | 89.75204 | 100.84 | 87.34526 | 433.4298 | 606.0167 | 501.9947 | 7.84E-15 |
| b1552 | *cspI* | 83.34118 | 82.74053 | 87.34526 | 970.2387 | 13350.73 | 6577.259 | 1.45E-14 |
| b3050 | *yqiJ* | 33.88598 | 29.73488 | 27.65933 | 235.5689 | 537.1512 | 377.7792 | 2.37E-14 |
| b3238 | *yhcN* | 176.7566 | 236.5862 | 235.8322 | 1690.079 | 6870.485 | 5764.213 | 2.93E-14 |
| b1905 | *ftnA* | 30.22263 | 19.39231 | 48.03989 | 289.3769 | 366.1351 | 345.9554 | 3.85E-14 |
| b1088 | *yceD* | 239.0335 | 372.3324 | 345.0138 | 2737.853 | 17132.6 | 12757.23 | 1.06E-13 |
| b0748 | *lysZ* | 10.99005 | 19.39231 | 10.19028 | 133.8845 | 222.6652 | 237.1386 | 1.32E-13 |
| b4108 | *yjdM* | 54.95023 | 76.27642 | 74.24347 | 377.0797 | 712.7583 | 744.2661 | 6.12E-13 |
| b4055 | *aphA* | 121.8063 | 99.5472 | 98.99129 | 736.7882 | 3111.574 | 2370.359 | 6.81E-13 |
| b4014 | *aceB* | 4729.383 | 5164.819 | 7833.414 | 32792.37 | 67173.74 | 56561.15 | 7.21E-13 |
| b0797 | *rhlE* | 261.9294 | 246.9288 | 196.5268 | 927.0228 | 899.843 | 915.704 | 1.34E-12 |
| b3703 | *rpmH* | 126.3855 | 151.26 | 141.2082 | 829.9989 | 2593.935 | 1722.591 | 1.69E-12 |
| b2287 | *nuoB* | 192.3258 | 259.857 | 295.5181 | 1342.658 | 2689.199 | 1745.176 | 3.27E-12 |
| b1826 | *mgrB* | 28.39095 | 32.32052 | 26.20358 | 282.1742 | 2666.244 | 972.1656 | 7.06E-12 |
| b2286 | *nuoC* | 290.3204 | 315.4483 | 384.3191 | 1508.319 | 3125.348 | 2249.224 | 7.52E-12 |
| b4756 | *yqiD* | 50.37105 | 60.76258 | 65.50894 | 302.0874 | 399.4201 | 267.9358 | 1.81E-11 |
| b0214 | *rnhA* | 62.27693 | 43.95591 | 58.23017 | 292.3427 | 655.3704 | 494.8087 | 1.96E-11 |
| b4554 | *yibT* | 138.2914 | 138.3318 | 168.8675 | 947.3597 | 6715.538 | 4036.489 | 2.32E-11 |
| b4736 | *yliM* | 85.17286 | 93.08309 | 131.0179 | 468.5956 | 794.2492 | 864.3753 | 5.69E-11 |
| b0721 | *sdhC* | 159.3557 | 135.7462 | 168.8675 | 899.907 | 15913.68 | 8119.174 | 6.91E-11 |
| b3194 | *mlaE* | 160.2715 | 138.3318 | 142.6639 | 566.0432 | 1060.529 | 961.8998 | 7.39E-11 |
| b4201 | *priB* | 66.85611 | 106.0113 | 97.53554 | 522.4036 | 2566.389 | 2470.964 | 7.50E-11 |
| b4200 | *rpsF* | 196.905 | 283.1277 | 333.3677 | 1548.145 | 7836.898 | 6273.394 | 7.93E-11 |
| b3708 | *tnaA* | 97.99458 | 67.22668 | 61.14168 | 405.8902 | 703.5762 | 985.511 | 8.00E-11 |
| b1089 | *rpmF* | 127.3014 | 253.3929 | 267.8588 | 1402.397 | 11689.92 | 8245.442 | 8.74E-11 |
| b1600 | *mdtJ* | 74.18281 | 68.5195 | 50.9514 | 505.8799 | 1443.881 | 543.0576 | 9.56E-11 |
| b0722 | *sdhD* | 30.22263 | 42.66308 | 37.84961 | 218.1978 | 4655.31 | 2280.021 | 1.14E-10 |
| b4226 | *ppa* | 204.2317 | 271.4924 | 355.204 | 1427.395 | 4354.597 | 3575.557 | 1.15E-10 |
| b2872 | *ygeY* | 138.2914 | 133.1605 | 125.1949 | 878.299 | 891.8087 | 2306.712 | 1.58E-10 |
| b0436 | *tig* | 326.038 | 346.476 | 371.2173 | 1718.466 | 9861.545 | 8404.561 | 1.98E-10 |
| b0723 | *sdhA* | 277.4987 | 239.1718 | 304.2526 | 1361.724 | 22890.9 | 13479.94 | 2.52E-10 |
| b2237 | *inaA* | 212.4742 | 191.3375 | 184.8808 | 947.3597 | 3919.597 | 2590.046 | 3.43E-10 |
| b2342 | *fadI* | 163.9349 | 166.7739 | 125.1949 | 676.2013 | 14006.1 | 9841.765 | 3.93E-10 |
| b0724 | *sdhB* | 97.07874 | 111.1826 | 132.4736 | 531.7247 | 11161.96 | 6145.072 | 4.98E-10 |
| b4016 | *aceK* | 1610.958 | 1853.905 | 2310.282 | 7541.169 | 17375.92 | 14243.71 | 5.99E-10 |
| b0432 | *cyoA* | 476.2353 | 469.2939 | 461.4741 | 2218.415 | 17774.19 | 12049.93 | 6.52E-10 |
| b1126 | *potA* | 230.791 | 217.1939 | 232.9207 | 716.4513 | 996.2548 | 763.771 | 6.93E-10 |
| b0775 | *bioB* | 92.49956 | 89.20463 | 80.06649 | 342.7612 | 671.439 | 491.7289 | 7.05E-10 |
| b0929 | *ompF* | 125.4697 | 140.9175 | 139.7524 | 670.2697 | 8098.587 | 4250.016 | 8.60E-10 |
| b2344 | *fadL* | 102.5738 | 91.79027 | 77.15498 | 290.6479 | 14923.16 | 8001.118 | 1.32E-09 |
| b3672 | *ivbL* | 6.41086 | 3.878462 | 4.367263 | 30.50532 | 940.0146 | 583.094 | 1.80E-09 |
| b0122 | *yacC* | 100.7421 | 82.74053 | 82.97799 | 334.2875 | 651.9271 | 511.2338 | 1.87E-09 |
| b3193 | *mlaD* | 128.2172 | 121.5252 | 135.3851 | 390.2139 | 541.7422 | 537.9248 | 1.95E-09 |
| b2609 | *rpsP* | 104.4054 | 160.3098 | 173.2348 | 677.896 | 3064.516 | 2661.906 | 2.01E-09 |
| b3186 | *rplU* | 362.6715 | 453.7801 | 486.2219 | 1809.135 | 7536.185 | 5970.554 | 2.58E-09 |
| b4202 | *rpsR* | 83.34118 | 95.66874 | 112.0931 | 422.8376 | 2556.059 | 2205.081 | 3.04E-09 |
| b1611 | *fumC* | 163.019 | 182.2877 | 216.9074 | 560.5353 | 20020.36 | 13853.62 | 3.84E-09 |
| b2175 | *mepS* | 85.17286 | 98.25438 | 98.99129 | 408.4323 | 2876.284 | 1924.826 | 4.55E-09 |
| b0777 | *bioC* | 33.88598 | 32.32052 | 29.11509 | 132.6134 | 234.1428 | 194.0225 | 4.69E-09 |
| b4724 | *ynfQ* | 7.326697 | 6.464104 | 5.823017 | 47.45272 | 837.864 | 297.7065 | 4.97E-09 |
| b1558 | *cspF* | 35.71765 | 23.27077 | 27.65933 | 166.5082 | 765.5552 | 374.6995 | 5.51E-09 |
| b2284 | *nuoF* | 157.524 | 143.5031 | 163.0445 | 541.4694 | 1153.498 | 822.2858 | 6.17E-09 |
| b2533 | *suhB* | 106.2371 | 111.1826 | 164.5002 | 513.0825 | 881.4789 | 603.6255 | 7.03E-09 |
| b0910 | *cmk* | 253.6869 | 261.1498 | 221.2747 | 952.0202 | 3360.638 | 2320.057 | 7.06E-09 |
| b0431 | *cyoB* | 563.2399 | 571.4268 | 604.138 | 2268.833 | 16983.39 | 11758.38 | 7.61E-09 |
| b1891 | *flhC* | 239.9493 | 219.7795 | 209.6286 | 1709.569 | 1193.669 | 767.8773 | 8.72E-09 |
| b1269 | *rluB* | 137.3756 | 127.9893 | 122.2834 | 490.2036 | 1691.797 | 1223.676 | 9.49E-09 |
| b2881 | *xdhD* | 297.6471 | 254.6857 | 260.58 | 1143.102 | 972.1518 | 1873.498 | 9.56E-09 |
| b0434 | *yajG* | 47.62353 | 34.90616 | 18.92481 | 192.353 | 948.0489 | 578.9877 | 1.14E-08 |
| b0811 | *glnH* | 86.0887 | 86.61899 | 69.87621 | 249.9742 | 5337.079 | 4264.388 | 1.34E-08 |
| b0877 | *ybjX* | 203.3159 | 213.3154 | 315.8987 | 1060.907 | 1999.396 | 1103.567 | 1.92E-08 |
| b1976 | *mtfA* | 112.648 | 90.49745 | 100.447 | 380.0455 | 1697.535 | 1363.29 | 2.37E-08 |
| b4599 | *mgtS* | 51.28688 | 69.81232 | 39.30537 | 239.382 | 4609.4 | 1416.672 | 2.43E-08 |
| b2871 | *ygeX* | 65.94028 | 72.39796 | 49.49565 | 353.777 | 494.6841 | 1381.769 | 2.55E-08 |
| b1274 | *topA* | 625.5168 | 729.1509 | 800.6649 | 2395.515 | 7077.082 | 5896.641 | 2.93E-08 |
| b4314 | *fimA* | 223.4643 | 327.0837 | 404.6997 | 1258.344 | 4824.031 | 7200.39 | 3.82E-08 |
| b2341 | *fadJ* | 261.9294 | 253.3929 | 195.0711 | 668.1513 | 13657.18 | 9932.103 | 3.98E-08 |
| b0774 | *bioA* | 136.4597 | 156.4313 | 117.9161 | 471.1377 | 661.1091 | 442.4534 | 5.15E-08 |
| b3846 | *fadB* | 187.7466 | 138.3318 | 123.7391 | 205.0635 | 21387.34 | 14908.93 | 5.98E-08 |
| b1841 | *yobA* | 206.9792 | 235.2934 | 199.4383 | 705.8592 | 1284.342 | 800.7277 | 6.07E-08 |
| b4735 | *ybgU* | 23.81177 | 25.85642 | 26.20358 | 84.31332 | 1502.416 | 791.4885 | 8.64E-08 |
| b4018 | *iclR* | 221.6326 | 212.0226 | 270.7703 | 1363.842 | 911.3206 | 739.1333 | 1.07E-07 |
| b3236 | *mdh* | 727.1747 | 808.013 | 936.05 | 2015.893 | 47661.84 | 30682.24 | 1.15E-07 |
| b2209 | *eco* | 96.1629 | 109.8898 | 98.99129 | 332.5927 | 1375.015 | 1511.117 | 1.30E-07 |
| b1782 | *mipA* | 101.6579 | 129.2821 | 128.1064 | 394.0271 | 1620.636 | 1451.576 | 1.33E-07 |
| b1911 | *glyW* | 18.31674 | 32.32052 | 21.83631 | 95.75281 | 755.2254 | 512.2604 | 1.34E-07 |
| b0931 | *pncB* | 319.6272 | 321.9124 | 397.4209 | 1220.213 | 4207.684 | 2359.067 | 1.37E-07 |
| b2876 | *yqeC* | 93.41539 | 63.34822 | 36.39386 | 302.5111 | 414.341 | 495.8352 | 1.38E-07 |
| b3231 | *rplM* | 502.7946 | 733.0294 | 902.5677 | 2487.455 | 11354.78 | 8775.154 | 1.38E-07 |
| b4000 | *hupA* | 89.75204 | 85.32617 | 87.34526 | 262.261 | 3101.245 | 2046.989 | 1.51E-07 |
| b3107 | *yhaL* | 41.21267 | 42.66308 | 68.42045 | 157.1871 | 2368.974 | 1472.107 | 1.53E-07 |
| b4217 | *ytfK* | 53.11856 | 108.5969 | 84.43375 | 274.9716 | 2810.862 | 2021.324 | 1.54E-07 |
| b4021 | *pepE* | 74.18281 | 68.5195 | 77.15498 | 323.2717 | 229.5518 | 237.1386 | 1.69E-07 |
| b4207 | *fklB* | 77.84616 | 94.37592 | 116.4603 | 319.0348 | 1480.609 | 1413.592 | 1.75E-07 |
| b0728 | *sucC* | 276.5828 | 311.5698 | 358.1156 | 740.1777 | 14953 | 10767.73 | 2.27E-07 |
| b3737 | *atpE* | 91.58372 | 144.7959 | 113.5488 | 379.1981 | 2468.829 | 1772.893 | 2.66E-07 |
| b2091 | *gatD* | 153.8606 | 161.6026 | 177.602 | 1875.23 | 738.009 | 706.2829 | 2.74E-07 |
| b1599 | *mdtI* | 61.36109 | 38.78462 | 48.03989 | 217.7741 | 399.4201 | 210.4477 | 2.77E-07 |
| b4163 | *glyV* | 46.7077 | 53.00565 | 64.05319 | 196.5898 | 906.7296 | 522.5262 | 3.43E-07 |
| b0464 | *acrR* | 35.71765 | 29.73488 | 37.84961 | 120.3265 | 1322.218 | 588.2269 | 3.43E-07 |
| b3738 | *atpB* | 204.2317 | 221.0723 | 295.5181 | 751.6172 | 4021.747 | 2914.444 | 3.54E-07 |
| b3157 | *yhbT* | 161.1873 | 237.879 | 241.6552 | 707.9776 | 698.9852 | 638.529 | 3.55E-07 |
| b3704 | *rnpA* | 52.20272 | 77.56925 | 87.34526 | 263.9558 | 467.1379 | 311.0519 | 3.57E-07 |
| b2880 | *ygfM* | 51.28688 | 32.32052 | 56.77442 | 274.9716 | 171.0161 | 366.4869 | 3.62E-07 |
| b0221 | *fadE* | 727.1747 | 617.9683 | 703.1293 | 516.0483 | 94287.25 | 65185.39 | 3.68E-07 |
| b2608 | *rimM* | 174.0091 | 280.5421 | 279.5048 | 761.362 | 3783.013 | 3394.88 | 4.04E-07 |
| b0961 | *yccF* | 38.46516 | 45.24873 | 45.12838 | 137.2739 | 266.2801 | 219.6868 | 4.34E-07 |
| b2679 | *proX* | 286.657 | 371.0396 | 532.8061 | 1433.75 | 3848.436 | 2305.685 | 4.71E-07 |
| b0430 | *cyoC* | 86.0887 | 93.08309 | 104.8143 | 267.7689 | 2544.582 | 1653.811 | 5.27E-07 |
| b1840 | *yebZ* | 252.7711 | 237.879 | 286.7836 | 644.0012 | 897.5475 | 732.9738 | 7.26E-07 |
| b3637 | *rpmB* | 723.5114 | 1013.571 | 1055.422 | 2752.258 | 13885.59 | 9612.839 | 7.54E-07 |
| b0905 | *ycaO* | 152.9448 | 156.4313 | 135.3851 | 391.4849 | 709.315 | 762.7445 | 7.61E-07 |
| b1090 | *plsX* | 204.2317 | 258.5642 | 330.4562 | 819.8305 | 1745.741 | 1213.41 | 7.79E-07 |
| b2504 | *yfgG* | 14.65339 | 25.85642 | 26.20358 | 87.7028 | 810.3178 | 325.424 | 7.86E-07 |
| b1304 | *pspA* | 315.9638 | 502.9073 | 547.3636 | 1987.93 | 23254.74 | 4627.795 | 7.93E-07 |
| b0726 | *sucA* | 524.7747 | 477.0509 | 633.2531 | 1132.51 | 21691.5 | 14223.18 | 1.02E-06 |
| b0720 | *gltA* | 516.5322 | 497.736 | 641.9876 | 1181.657 | 23717.29 | 12994.37 | 1.08E-06 |
| b2346 | *mlaA* | 171.2616 | 179.7021 | 158.6772 | 477.9167 | 1942.008 | 1437.204 | 1.11E-06 |
| b2350 | *yfdG* | 22.89593 | 14.22103 | 10.19028 | 64.82381 | 369.5784 | 232.0057 | 1.36E-06 |
| b0727 | *sucB* | 282.9937 | 320.6195 | 359.5713 | 624.9354 | 12908.84 | 8527.75 | 1.38E-06 |
| b4557 | *yidD* | 27.47512 | 47.83437 | 32.02659 | 147.8661 | 157.243 | 130.3749 | 1.41E-06 |
| b0729 | *sucD* | 268.3403 | 285.7134 | 372.6731 | 584.6853 | 12672.41 | 8888.077 | 1.41E-06 |
| b4059 | *ssb* | 329.7014 | 427.9237 | 387.2306 | 1006.676 | 2317.325 | 1829.355 | 1.41E-06 |
| b2171 | *yeiP* | 48.53937 | 65.93386 | 45.12838 | 172.8635 | 478.6155 | 323.3708 | 1.43E-06 |
| b0947 | *ycbX* | 172.1774 | 147.3816 | 183.425 | 429.1929 | 802.2835 | 712.4423 | 1.43E-06 |
| b3736 | *atpF* | 251.8552 | 299.9344 | 398.8767 | 878.299 | 5231.485 | 3816.802 | 1.46E-06 |
| b3403 | *pck* | 373.6616 | 376.2108 | 397.4209 | 766.4462 | 14420.44 | 8838.802 | 1.59E-06 |
| b2281 | *nuoI* | 54.03439 | 45.24873 | 52.40715 | 151.2555 | 353.5098 | 268.9624 | 1.64E-06 |
| b1415 | *aldA* | 274.7512 | 266.3211 | 165.956 | 369.4533 | 11451.19 | 8891.157 | 1.83E-06 |
| b1944 | *fliL* | 32.97014 | 21.97795 | 29.11509 | 100.837 | 164.1295 | 247.4043 | 1.87E-06 |
| b0525 | *ppiB* | 174.0091 | 200.3872 | 219.8189 | 530.8773 | 1976.441 | 1574.764 | 1.98E-06 |
| b3644 | *yicC* | 178.5883 | 174.5308 | 199.4383 | 451.2245 | 839.0118 | 740.1598 | 2.13E-06 |
| b1656 | *sodB* | 514.7005 | 590.8191 | 720.5984 | 1524.419 | 2797.089 | 2890.832 | 2.17E-06 |
| b1608 | *rstA* | 130.0489 | 156.4313 | 100.447 | 406.7376 | 638.154 | 415.7625 | 2.20E-06 |
| b1075 | *flgD* | 38.46516 | 36.19898 | 34.9381 | 127.5292 | 236.4383 | 469.1443 | 2.22E-06 |
| b0684 | *fldA* | 87.92037 | 111.1826 | 116.4603 | 298.6979 | 2220.914 | 1122.045 | 2.37E-06 |
| b1915 | *yecF* | 23.81177 | 29.73488 | 18.92481 | 85.58437 | 487.7975 | 245.3512 | 2.43E-06 |
| b0668 | *glnW* | 9.158372 | 11.63539 | 11.64603 | 42.3685 | 146.9131 | 146.8001 | 2.51E-06 |
| b3977 | *tyrU* | 106.2371 | 125.4036 | 122.2834 | 332.5927 | 2538.843 | 1184.666 | 2.82E-06 |
| b3207 | *yrbL* | 1039.475 | 1502.258 | 1752.728 | 4031.363 | 21080.89 | 12803.43 | 2.88E-06 |
| b3310 | *rplN* | 754.6498 | 1304.456 | 1489.237 | 3495.825 | 11392.66 | 9118.03 | 2.89E-06 |
| b2667 | *ygaV* | 33.88598 | 32.32052 | 8.734526 | 95.32913 | 2224.357 | 404.4701 | 2.90E-06 |
| b3742 | *mioC* | 256.4344 | 308.9842 | 369.7616 | 877.028 | 1090.371 | 806.8871 | 2.96E-06 |
| b3927 | *glpF* | 142.8706 | 111.1826 | 112.0931 | 242.7715 | 4175.547 | 2563.355 | 3.01E-06 |
| b0610 | *rnk* | 33.88598 | 25.85642 | 27.65933 | 58.46853 | 864.2625 | 833.5781 | 3.61E-06 |
| b0776 | *bioF* | 106.2371 | 126.6964 | 81.52224 | 294.8848 | 561.2541 | 427.0548 | 3.70E-06 |
| b3055 | *ygiM* | 73.26697 | 80.15489 | 61.14168 | 191.5056 | 415.4887 | 363.4072 | 3.96E-06 |
| b2966 | *yqgA* | 149.2815 | 161.6026 | 126.6506 | 347.8454 | 432.7051 | 403.4436 | 4.30E-06 |
| b1660 | *ydhC* | 144.7023 | 106.0113 | 110.6373 | 313.9506 | 874.5923 | 858.2158 | 4.33E-06 |
| b1922 | *fliA* | 37.54932 | 42.66308 | 33.48235 | 114.395 | 250.2114 | 373.6729 | 4.48E-06 |
| b4062 | *soxS* | 87.92037 | 118.9395 | 141.2082 | 259.7189 | 4022.895 | 1814.983 | 4.48E-06 |
| b1305 | *pspB* | 42.12851 | 56.88411 | 58.23017 | 190.2346 | 3012.867 | 457.852 | 4.55E-06 |
| b0651 | *rihA* | 170.3457 | 134.4534 | 125.1949 | 368.1823 | 766.703 | 876.6942 | 4.55E-06 |
| b1551 | *ynfN* | 5.495023 | 1.292821 | 0 | 24.99742 | 142.3221 | 95.47138 | 4.81E-06 |
| b3926 | *glpK* | 209.7267 | 236.5862 | 147.0312 | 355.8954 | 6285.128 | 4453.278 | 4.96E-06 |
| b4052 | *dnaB* | 165.7665 | 197.8016 | 203.8056 | 450.3772 | 820.6476 | 709.3626 | 4.96E-06 |
| b1781 | *yeaE* | 120.8905 | 135.7462 | 154.31 | 345.3033 | 764.4074 | 604.6521 | 5.18E-06 |
| b0411 | *tsx* | 124.5539 | 77.56925 | 133.9294 | 300.3927 | 1088.075 | 1318.121 | 5.23E-06 |
| b0406 | *tgt* | 138.2914 | 165.4811 | 179.0578 | 389.7902 | 2696.086 | 1730.804 | 5.23E-06 |
| b2232 | *ubiG* | 167.5982 | 201.68 | 206.7171 | 455.8851 | 736.8612 | 599.5192 | 5.51E-06 |
| b3357 | *crp* | 740.9123 | 1016.157 | 1118.019 | 2556.515 | 9572.31 | 5996.219 | 5.84E-06 |
| b1275 | *cysB* | 282.9937 | 283.1277 | 324.6332 | 771.9541 | 5697.475 | 2585.94 | 6.07E-06 |
| b0838 | *gstB* | 139.2073 | 115.061 | 120.8276 | 297.8506 | 1406.005 | 1242.155 | 6.60E-06 |
| b3418 | *malT* | 391.0625 | 395.6032 | 336.2792 | 935.4965 | 4141.114 | 2617.764 | 6.63E-06 |
| b1251 | *yciI* | 71.4353 | 65.93386 | 50.9514 | 167.3556 | 622.0853 | 490.7024 | 6.63E-06 |
| b0131 | *panD* | 60.44525 | 50.42001 | 53.86291 | 141.5108 | 907.8773 | 548.1905 | 6.67E-06 |
| b0637 | *rsfS* | 68.68779 | 112.4754 | 110.6373 | 279.2084 | 538.2989 | 406.5233 | 6.80E-06 |
| b0743 | *lysT* | 129.133 | 212.0226 | 170.3233 | 444.8693 | 1279.751 | 1056.345 | 7.61E-06 |
| b3748 | *rbsD* | 31.13846 | 29.73488 | 13.10179 | 71.17908 | 391.3858 | 487.6226 | 7.90E-06 |
| b1839 | *yebY* | 78.762 | 90.49745 | 61.14168 | 207.6057 | 570.4362 | 406.5233 | 9.42E-06 |
| b0407 | *yajC* | 130.0489 | 169.3595 | 147.0312 | 313.9506 | 2592.787 | 1895.056 | 9.88E-06 |
| b1777 | *yeaC* | 46.7077 | 41.37026 | 49.49565 | 127.9529 | 412.0455 | 264.8561 | 9.97E-06 |
| b3175 | *secG* | 183.1674 | 148.6744 | 157.2215 | 387.2481 | 2030.386 | 1357.131 | 9.97E-06 |
| b2283 | *nuoG* | 525.6905 | 504.2001 | 621.6071 | 1289.697 | 2799.384 | 2069.573 | 1.00E-05 |
| b2259 | *pmrD* | 39.381 | 29.73488 | 30.57084 | 92.36333 | 437.2962 | 285.3876 | 1.01E-05 |
| b2243 | *glpC* | 67.77195 | 63.34822 | 69.87621 | 174.9819 | 255.9502 | 190.9428 | 1.03E-05 |
| b0778 | *bioD* | 54.95023 | 38.78462 | 55.31866 | 143.2055 | 231.8473 | 174.5176 | 1.05E-05 |
| b0781 | *moaA* | 130.9647 | 143.5031 | 122.2834 | 319.4585 | 1534.554 | 991.6705 | 1.09E-05 |
| b0410 | *yajD* | 39.381 | 25.85642 | 29.11509 | 91.51596 | 303.0084 | 229.9526 | 1.10E-05 |
| b1986 | *asnU* | 26.55928 | 28.44206 | 24.74782 | 90.24491 | 771.294 | 204.2882 | 1.19E-05 |
| b3309 | *rplX* | 667.6453 | 1098.898 | 1192.263 | 2471.778 | 7693.428 | 6912.949 | 1.25E-05 |
| b4522 | *ymiA* | 35.71765 | 37.4918 | 30.57084 | 124.5634 | 258.2458 | 123.1889 | 1.30E-05 |
| b3185 | *rpmA* | 130.0489 | 210.7298 | 237.2879 | 441.0561 | 3710.705 | 2159.912 | 1.30E-05 |
| b2398 | *yfeC* | 71.4353 | 60.76258 | 62.59743 | 156.7635 | 505.0139 | 546.1374 | 1.31E-05 |
| b1557 | *cspB* | 147.4498 | 108.5969 | 71.33196 | 258.0242 | 4306.392 | 1255.5 | 1.32E-05 |
| b2517 | *rlmN* | 189.5783 | 202.9729 | 216.9074 | 482.1535 | 1443.881 | 1020.415 | 1.32E-05 |
| b0970 | *yccA* | 230.791 | 222.3652 | 234.3764 | 547.401 | 3440.981 | 1751.335 | 1.34E-05 |
| b2242 | *glpB* | 108.9846 | 155.1385 | 82.97799 | 355.048 | 412.0455 | 364.4338 | 1.38E-05 |
| b3962 | *sthA* | 660.3186 | 624.4324 | 762.8152 | 1892.177 | 6652.411 | 3033.526 | 1.40E-05 |
| b1859 | *znuB* | 225.2959 | 281.8349 | 225.6419 | 541.4694 | 698.9852 | 794.5683 | 1.42E-05 |
| b0810 | *glnP* | 27.47512 | 27.14924 | 52.40715 | 80.92384 | 775.885 | 558.4562 | 1.46E-05 |
| b1307 | *pspD* | 37.54932 | 41.37026 | 33.48235 | 110.1581 | 1533.406 | 307.9722 | 1.53E-05 |
| b4189 | *bsmA* | 75.09865 | 67.22668 | 88.80101 | 185.9977 | 1272.865 | 655.9808 | 1.63E-05 |
| b1203 | *ychF* | 84.25702 | 93.08309 | 117.9161 | 223.7057 | 1550.622 | 917.7571 | 1.71E-05 |
| b0174 | *ispU* | 261.9294 | 332.2549 | 361.0271 | 726.6198 | 3506.404 | 2351.881 | 1.77E-05 |
| b2668 | *ygaP* | 88.83621 | 93.08309 | 84.43375 | 225.8241 | 1453.063 | 637.5024 | 1.77E-05 |
| b0435 | *bolA* | 143.7864 | 175.8236 | 177.602 | 326.2375 | 3463.936 | 1913.534 | 1.80E-05 |
| b2765 | *queD* | 21.98009 | 29.73488 | 33.48235 | 82.19489 | 282.3487 | 185.8099 | 1.83E-05 |
| b2875 | *yqeB* | 252.7711 | 182.2877 | 218.3631 | 641.0354 | 566.9929 | 1023.494 | 1.85E-05 |
| b3845 | *fadA* | 226.2118 | 257.2713 | 197.9826 | 140.6634 | 13303.67 | 8904.503 | 1.89E-05 |
| b2215 | *ompC* | 2584.493 | 3640.583 | 4375.997 | 7377.627 | 51130.36 | 37875.45 | 1.91E-05 |
| b3707 | *tnaC* | 5.495023 | 0 | 2.911509 | 27.96321 | 66.57002 | 51.3287 | 1.93E-05 |
| b4043 | *lexA* | 169.4299 | 187.459 | 218.3631 | 443.1745 | 3396.219 | 1560.392 | 1.94E-05 |
| b1076 | *flgE* | 55.86607 | 59.46975 | 42.21687 | 158.8819 | 280.0532 | 638.529 | 1.96E-05 |
| b1073 | *flgB* | 20.14842 | 16.80667 | 36.39386 | 78.80541 | 177.9026 | 252.5372 | 2.03E-05 |
| b4068 | *yjcH* | 25.64344 | 15.51385 | 7.278771 | 29.65795 | 764.4074 | 403.4436 | 2.06E-05 |
| b0125 | *hpt* | 124.5539 | 138.3318 | 147.0312 | 308.8664 | 556.6631 | 444.5065 | 2.09E-05 |
| b3190 | *ibaG* | 23.81177 | 36.19898 | 43.67263 | 109.7344 | 146.9131 | 126.2686 | 2.16E-05 |
| b3631 | *waaG* | 182.2516 | 165.4811 | 179.0578 | 378.3507 | 515.3438 | 616.971 | 2.24E-05 |
| b2817 | *amiC* | 170.3457 | 139.6246 | 139.7524 | 352.0822 | 926.2415 | 659.0605 | 2.35E-05 |
| b3041 | *ribB* | 514.7005 | 469.2939 | 589.5805 | 1164.71 | 6614.535 | 3758.287 | 2.35E-05 |
| b4618 | *tisB* | 37.54932 | 50.42001 | 48.03989 | 71.60277 | 1757.219 | 696.0172 | 2.37E-05 |
| b1136 | *icd* | 1120.069 | 1097.605 | 1390.245 | 1900.227 | 27750.52 | 17625.25 | 2.45E-05 |
| b2310 | *argT* | 95.24707 | 94.37592 | 71.33196 | 109.3107 | 3152.894 | 1728.751 | 2.48E-05 |
| b3735 | *atpH* | 151.1131 | 152.5528 | 209.6286 | 350.8112 | 2357.497 | 1676.395 | 2.58E-05 |
| b1308 | *pspE* | 105.3213 | 165.4811 | 144.1197 | 345.3033 | 3925.336 | 1077.903 | 2.61E-05 |
| b3081 | *fadH* | 215.2217 | 187.459 | 112.0931 | 234.7215 | 4658.754 | 3771.633 | 2.64E-05 |
| b3471 | *yhhQ* | 149.2815 | 164.1882 | 209.6286 | 411.3981 | 1007.732 | 730.9207 | 2.76E-05 |
| b1930 | *yedF* | 18.31674 | 19.39231 | 14.55754 | 56.77379 | 80.34313 | 89.31194 | 2.90E-05 |
| b3546 | *eptB* | 257.3502 | 246.9288 | 227.0977 | 535.1142 | 1212.033 | 906.4648 | 2.99E-05 |
| b0237 | *pepD* | 724.4272 | 811.8914 | 998.6474 | 1834.556 | 6072.793 | 4830.031 | 3.00E-05 |
| b2027 | *wzzB* | 481.7304 | 555.9129 | 583.7575 | 1273.173 | 5045.548 | 2711.182 | 3.10E-05 |
| b1306 | *pspC* | 59.52942 | 94.37592 | 104.8143 | 229.2136 | 3209.134 | 635.4493 | 3.10E-05 |
| b3629 | *waaS* | 92.49956 | 77.56925 | 96.07978 | 202.9451 | 241.0294 | 249.4575 | 3.21E-05 |
| b3059 | *plsY* | 101.6579 | 80.15489 | 72.78771 | 205.4872 | 438.4439 | 337.7428 | 3.24E-05 |
| b1074 | *flgC* | 24.7276 | 24.56359 | 17.46905 | 71.60277 | 134.2878 | 317.2114 | 3.29E-05 |
| b3623 | *waaU* | 83.34118 | 91.79027 | 82.97799 | 192.353 | 234.1428 | 227.8994 | 3.30E-05 |
| b1418 | *cybB* | 83.34118 | 85.32617 | 72.78771 | 210.9951 | 518.787 | 291.547 | 3.31E-05 |
| b4702 | *mgtL* | 32.0543 | 40.07744 | 56.77442 | 102.1081 | 592.2436 | 373.6729 | 3.59E-05 |
| b1854 | *pykA* | 559.5765 | 707.173 | 807.9436 | 1622.29 | 1865.108 | 1565.525 | 4.15E-05 |
| b2595 | *bamD* | 220.7168 | 234.0006 | 240.1995 | 489.7799 | 2235.834 | 1437.204 | 4.18E-05 |
| b4742 | *ymjE* | 4.579186 | 5.171283 | 4.367263 | 14.40529 | 210.0399 | 78.01962 | 4.21E-05 |
| b0820 | *ybiT* | 392.8942 | 409.8242 | 508.0582 | 943.1228 | 2690.347 | 1997.713 | 4.35E-05 |
| b2830 | *rppH* | 171.2616 | 208.1441 | 263.4915 | 463.9351 | 2088.921 | 1444.39 | 4.41E-05 |
| b3670 | *ilvN* | 51.28688 | 29.73488 | 33.48235 | 72.02645 | 671.439 | 534.845 | 4.47E-05 |
| b0730 | *mngR* | 78.762 | 74.9836 | 81.52224 | 197.0135 | 856.2282 | 399.3373 | 4.51E-05 |
| b1093 | *fabG* | 380.0724 | 514.5427 | 567.7442 | 902.0254 | 6701.764 | 4814.632 | 4.51E-05 |
| b3624 | *waaZ* | 44.87602 | 37.4918 | 43.67263 | 127.1055 | 106.7416 | 150.9064 | 4.53E-05 |
| b0386 | *proC* | 161.1873 | 164.1882 | 176.1463 | 360.1323 | 835.5685 | 632.3696 | 4.53E-05 |
| b0678 | *nagB* | 124.5539 | 104.7185 | 138.2967 | 433.0061 | 493.5363 | 265.8827 | 4.85E-05 |
| b2790 | *yqcA* | 131.8806 | 170.6523 | 145.5754 | 332.169 | 824.0909 | 594.3863 | 5.31E-05 |
| b4147 | *efp* | 163.019 | 196.5088 | 187.7923 | 379.6218 | 1216.624 | 984.4844 | 5.39E-05 |
| b3192 | *mlaC* | 237.2018 | 280.5421 | 339.1907 | 607.1406 | 1649.33 | 1411.539 | 5.40E-05 |
| b2450 | *yffS* | 117.2272 | 129.2821 | 119.3719 | 253.7873 | 457.9558 | 468.1177 | 5.63E-05 |
| b2282 | *nuoH* | 87.92037 | 108.5969 | 107.7258 | 228.7899 | 765.5552 | 497.8884 | 5.70E-05 |
| b0429 | *cyoD* | 104.4054 | 103.4257 | 115.0046 | 182.6082 | 1740.003 | 1142.577 | 5.81E-05 |
| b3356 | *yhfA* | 178.5883 | 168.0667 | 167.4117 | 352.0822 | 554.3676 | 455.7988 | 5.88E-05 |
| b1612 | *fumA* | 493.6362 | 438.2662 | 547.3636 | 782.5462 | 8087.109 | 5472.666 | 6.13E-05 |
| b0841 | *ybjG* | 119.9747 | 98.25438 | 100.447 | 227.5188 | 426.9663 | 464.0114 | 6.16E-05 |
| b3349 | *slyD* | 398.3892 | 513.2498 | 548.8194 | 938.886 | 4431.497 | 3573.504 | 6.49E-05 |
| b3588 | *aldB* | 112.648 | 134.4534 | 112.0931 | 224.5531 | 1298.115 | 1024.521 | 6.49E-05 |
| b3739 | *atpI* | 177.6724 | 209.437 | 187.7923 | 374.1139 | 2030.386 | 1348.918 | 6.51E-05 |
| b1163 | *bluF* | 143.7864 | 153.8457 | 145.5754 | 271.5821 | 1498.973 | 1262.686 | 6.67E-05 |
| b1778 | *msrB* | 132.7964 | 162.8954 | 158.6772 | 363.0981 | 1640.148 | 725.7878 | 6.81E-05 |
| b0632 | *dacA* | 173.0932 | 164.1882 | 184.8808 | 370.3007 | 902.1385 | 644.6885 | 6.95E-05 |
| b4050 | *pspG* | 75.09865 | 80.15489 | 84.43375 | 224.9767 | 3948.291 | 364.4338 | 6.95E-05 |
| b3279 | *yrdA* | 198.7367 | 221.0723 | 253.3012 | 464.3588 | 839.0118 | 696.0172 | 6.99E-05 |
| b0240 | *crl* | 119.9747 | 99.5472 | 126.6506 | 156.3398 | 3137.973 | 1436.177 | 7.23E-05 |
| b3165 | *rpsO* | 691.4571 | 972.2012 | 1139.856 | 1594.327 | 13941.83 | 9244.299 | 7.56E-05 |
| b3321 | *rpsJ* | 839.8227 | 1423.396 | 1703.233 | 2539.144 | 14574.24 | 12805.48 | 7.61E-05 |
| b2599 | *pheA* | 167.5982 | 186.1662 | 164.5002 | 344.4559 | 587.6526 | 585.1472 | 7.85E-05 |
| b0422 | *xseB* | 184.0833 | 192.6303 | 206.7171 | 395.7218 | 921.6504 | 742.213 | 7.91E-05 |
| b4049 | *dusA* | 75.09865 | 104.7185 | 94.62403 | 227.0952 | 607.1645 | 336.7163 | 7.93E-05 |
| b3671 | *ilvB* | 176.7566 | 201.68 | 136.8409 | 214.8083 | 3738.251 | 2610.578 | 8.01E-05 |
| b1479 | *maeA* | 883.7829 | 955.3945 | 1190.807 | 2125.628 | 3614.293 | 2699.89 | 8.52E-05 |
| b2277 | *nuoM* | 290.3204 | 297.3488 | 232.9207 | 577.4827 | 1177.601 | 862.3221 | 8.92E-05 |
| b3645 | *dinD* | 61.36109 | 68.5195 | 48.03989 | 155.0687 | 484.3543 | 240.2183 | 8.94E-05 |
| b1295 | *ymjA* | 55.86607 | 73.69078 | 68.42045 | 170.7451 | 294.974 | 180.677 | 9.46E-05 |
| b0791 | *ybhQ* | 79.67784 | 69.81232 | 109.1816 | 164.3898 | 1097.258 | 702.1766 | 9.66E-05 |
| b1929 | *yedE* | 115.3955 | 93.08309 | 94.62403 | 233.4504 | 239.8816 | 237.1386 | 9.92E-05 |
| b2879 | *ssnA* | 96.1629 | 76.27642 | 75.69922 | 268.1926 | 205.4488 | 483.5163 | 0.000102 |
| b0627 | *tatE* | 120.8905 | 98.25438 | 128.1064 | 196.5898 | 1455.358 | 1066.61 | 0.000103 |
| b2234 | *nrdA* | 187.7466 | 199.0944 | 240.1995 | 411.3981 | 1115.622 | 1078.929 | 0.000104 |
| b3075 | *ebgR* | 102.5738 | 102.1328 | 106.2701 | 464.3588 | 301.8606 | 230.9791 | 0.000108 |
| b3636 | *rpmG* | 134.6281 | 209.437 | 246.0225 | 357.5901 | 2382.748 | 1740.043 | 0.000109 |
| b3632 | *waaQ* | 256.4344 | 232.7077 | 315.8987 | 537.6563 | 966.413 | 934.1823 | 0.000111 |
| b2904 | *gcvH* | 93.41539 | 62.0554 | 109.1816 | 118.6318 | 1484.052 | 1367.397 | 0.000114 |
| b4203 | *rplI* | 169.4299 | 305.1057 | 312.9872 | 450.8008 | 3351.456 | 2636.242 | 0.000117 |
| b2977 | *glcG* | 53.11856 | 38.78462 | 53.86291 | 80.92384 | 571.5839 | 575.908 | 0.000127 |
| b3184 | *yhbE* | 171.2616 | 164.1882 | 189.2481 | 319.4585 | 1300.411 | 1163.108 | 0.000128 |
| b2426 | *ucpA* | 464.3295 | 562.377 | 655.0894 | 947.3597 | 5579.256 | 4619.583 | 0.000131 |
| b3836 | *tatA* | 195.9892 | 236.5862 | 272.2261 | 466.9009 | 1898.393 | 1235.995 | 0.000131 |
| b0763 | *modA* | 498.2154 | 524.8852 | 557.5539 | 998.2019 | 1153.498 | 1012.202 | 0.000132 |
| b2518 | *ndk* | 65.94028 | 76.27642 | 77.15498 | 126.6818 | 1005.437 | 597.4661 | 0.000132 |
| b4188 | *yjfN* | 71.4353 | 60.76258 | 40.76112 | 132.1897 | 896.3997 | 349.0352 | 0.000135 |
| b3528 | *dctA* | 264.6769 | 202.9729 | 250.3897 | 454.1903 | 1342.878 | 1354.051 | 0.000136 |
| b0428 | *cyoE* | 270.172 | 232.7077 | 212.5401 | 378.7744 | 3042.709 | 2100.37 | 0.000137 |
| b1092 | *fabD* | 177.6724 | 210.7298 | 243.111 | 390.2139 | 1742.298 | 1324.28 | 0.000141 |
| b0685 | *ybfE* | 35.71765 | 28.44206 | 36.39386 | 94.90544 | 137.7311 | 91.36508 | 0.000146 |
| b4596 | *yciZ* | 28.39095 | 29.73488 | 30.57084 | 70.33171 | 237.5861 | 144.7469 | 0.000152 |
| b2042 | *galF* | 347.1023 | 360.697 | 387.2306 | 683.8276 | 2670.835 | 1820.116 | 0.000163 |
| b2009 | *sbmC* | 78.762 | 103.4257 | 116.4603 | 183.8793 | 1070.859 | 684.7248 | 0.000165 |
| b1809 | *yoaB* | 88.83621 | 74.9836 | 94.62403 | 155.4924 | 721.9404 | 625.1836 | 0.000166 |
| b2584 | *patZ* | 446.9285 | 418.8739 | 493.5007 | 860.5042 | 2519.331 | 1900.188 | 0.000166 |
| b2792 | *yqcC* | 13.73756 | 7.756925 | 7.278771 | 30.50532 | 97.55951 | 78.01962 | 0.00017 |
| b0443 | *fadM* | 95.24707 | 67.22668 | 81.52224 | 110.5818 | 1634.409 | 842.8172 | 0.000176 |
| b3859 | *srkA* | 163.9349 | 165.4811 | 183.425 | 339.3717 | 558.9586 | 434.2408 | 0.000177 |
| b2240 | *glpT* | 176.7566 | 117.6467 | 94.62403 | 258.8715 | 1088.075 | 848.9767 | 0.000181 |
| b3749 | *rbsA* | 131.8806 | 143.5031 | 129.5621 | 261.4136 | 399.4201 | 362.3806 | 0.000182 |
| b1297 | *puuA* | 99.82625 | 87.91181 | 71.33196 | 137.6976 | 2542.286 | 624.157 | 0.000182 |
| b4069 | *acs* | 220.7168 | 255.9785 | 157.2215 | 169.8977 | 5820.286 | 3666.922 | 0.000185 |
| b4109 | *crfC* | 185.9149 | 161.6026 | 142.6639 | 303.7821 | 1508.155 | 936.2355 | 0.000189 |
| b3627 | *waaO* | 100.7421 | 102.1328 | 97.53554 | 198.2846 | 261.689 | 284.361 | 0.00019 |
| b1056 | *yceI* | 69.60363 | 73.69078 | 66.9647 | 154.645 | 274.3144 | 192.9959 | 0.00019 |
| b1002 | *agp* | 214.3059 | 166.7739 | 192.1596 | 352.0822 | 1078.893 | 1087.142 | 0.000191 |
| b2402 | *valX* | 43.04435 | 47.83437 | 26.20358 | 61.01064 | 583.0615 | 455.7988 | 0.000191 |
| b1091 | *fabH* | 209.7267 | 230.1221 | 286.7836 | 469.8667 | 1113.326 | 953.6872 | 0.000196 |
| b0421 | *ispA* | 272.9195 | 239.1718 | 299.8854 | 516.472 | 1055.938 | 900.3054 | 0.000197 |
| b1020 | *phoH* | 130.0489 | 133.1605 | 139.7524 | 138.545 | 4073.396 | 1445.416 | 0.000201 |
| b0846 | *rcdA* | 87.00453 | 60.76258 | 75.69922 | 152.1029 | 476.32 | 356.2212 | 0.000214 |
| b3316 | *rpsS* | 184.0833 | 275.3708 | 419.2572 | 464.7825 | 3515.586 | 3144.396 | 0.00022 |
| b0109 | *nadC* | 216.1376 | 162.8954 | 167.4117 | 370.3007 | 503.8662 | 446.5597 | 0.000222 |
| b2463 | *maeB* | 696.0363 | 711.0514 | 815.2224 | 994.8124 | 11005.86 | 6734.325 | 0.000226 |
| b1339 | *abgR* | 77.84616 | 102.1328 | 43.67263 | 186.4214 | 484.3543 | 336.7163 | 0.00023 |
| b3230 | *rpsI* | 239.9493 | 404.6529 | 496.4122 | 664.7618 | 4431.497 | 2889.806 | 0.000237 |
| b3733 | *atpG* | 327.8697 | 315.4483 | 426.536 | 537.6563 | 4059.623 | 2848.743 | 0.000254 |
| b1973 | *zinT* | 52.20272 | 51.71283 | 66.9647 | 277.5137 | 262.8368 | 113.9497 | 0.000254 |
| b3609 | *secB* | 183.1674 | 202.9729 | 250.3897 | 396.1455 | 1236.136 | 979.3516 | 0.000256 |
| b4484 | *cpxP* | 298.5629 | 227.5365 | 221.2747 | 464.7825 | 9339.314 | 1129.231 | 0.000258 |
| b3734 | *atpA* | 554.0815 | 696.8304 | 784.6516 | 1015.997 | 7700.315 | 5360.769 | 0.00026 |
| b4749 | *ynfR* | 0.915837 | 0 | 0 | 8.4737 | 48.20588 | 32.85037 | 0.00026 |
| b1218 | *chaC* | 265.5928 | 306.3985 | 246.0225 | 416.906 | 3677.42 | 1963.836 | 0.000262 |
| b2607 | *trmD* | 330.6172 | 544.2775 | 695.8506 | 866.0122 | 5264.77 | 4599.051 | 0.000263 |
| b4162 | *orn* | 94.33123 | 85.32617 | 94.62403 | 177.524 | 383.3515 | 327.4771 | 0.000268 |
| b0238 | *gpt* | 69.60363 | 72.39796 | 55.31866 | 133.4608 | 261.689 | 271.0155 | 0.000275 |
| b3630 | *waaP* | 110.8163 | 126.6964 | 106.2701 | 222.0109 | 301.8606 | 314.1316 | 0.000282 |
| b1805 | *fadD* | 339.7756 | 462.8298 | 387.2306 | 366.0638 | 8669.023 | 5032.266 | 0.000287 |
| b1856 | *mepM* | 398.3892 | 489.9791 | 612.8726 | 1185.894 | 1776.731 | 1116.912 | 0.000293 |
| b1806 | *yeaY* | 73.26697 | 98.25438 | 100.447 | 172.0161 | 867.7058 | 478.3835 | 0.000294 |
| b1103 | *hinT* | 153.8606 | 226.2436 | 234.3764 | 405.4666 | 902.1385 | 768.9039 | 0.000295 |
| b3784 | *rfe* | 276.5828 | 312.8626 | 275.1376 | 568.5853 | 1038.722 | 711.4158 | 0.0003 |
| b3936 | *rpmE* | 398.3892 | 813.1843 | 748.2577 | 1180.81 | 6211.671 | 4395.79 | 0.0003 |
| b3383 | *yhfZ* | 70.51946 | 55.59129 | 45.12838 | 122.445 | 227.2563 | 238.1652 | 0.000314 |
| b2899 | *yqfA* | 598.9575 | 676.1453 | 676.9257 | 1042.689 | 4956.023 | 3960.522 | 0.000319 |
| b3673 | *emrD* | 402.0525 | 354.2329 | 270.7703 | 680.0144 | 873.4445 | 918.7837 | 0.000329 |
| b1384 | *feaR* | 99.82625 | 80.15489 | 72.78771 | 81.34752 | 1985.623 | 969.0858 | 0.000333 |
| b1748 | *astC* | 181.3358 | 153.8457 | 167.4117 | 182.1846 | 2552.616 | 1905.321 | 0.000347 |
| b4450 | *arcZ* | 11.90588 | 19.39231 | 16.0133 | 39.40271 | 235.2906 | 88.28536 | 0.000363 |
| b4216 | *ytfJ* | 90.66788 | 60.76258 | 91.71252 | 137.6976 | 695.5419 | 555.3765 | 0.000365 |
| b3318 | *rplW* | 283.9095 | 480.9293 | 614.3283 | 711.7908 | 4776.973 | 4066.26 | 0.000367 |
| b1488 | *ddpX* | 36.63349 | 27.14924 | 24.74782 | 43.63956 | 514.196 | 256.6435 | 0.000393 |
| b1938 | *fliF* | 103.4896 | 103.4257 | 87.34526 | 206.3346 | 257.098 | 393.1778 | 0.000397 |
| b2309 | *hisJ* | 84.25702 | 67.22668 | 106.2701 | 101.2607 | 1647.034 | 802.7808 | 0.000403 |
| b0912 | *ihfB* | 218.8851 | 239.1718 | 280.9606 | 369.4533 | 2612.299 | 1642.518 | 0.000403 |
| b1104 | *ycfL* | 79.67784 | 120.2323 | 132.4736 | 217.3504 | 549.7765 | 487.6226 | 0.000406 |
| b4725 | *rseD* | 70.51946 | 46.54155 | 39.30537 | 119.4792 | 464.8424 | 226.8728 | 0.000411 |
| b4067 | *actP* | 140.1231 | 142.2103 | 90.25677 | 134.3081 | 2453.909 | 1226.756 | 0.000437 |
| b0469 | *apt* | 82.42535 | 63.34822 | 94.62403 | 166.5082 | 391.3858 | 269.989 | 0.00044 |
| b0750 | *nadA* | 64.1086 | 55.59129 | 58.23017 | 143.6292 | 135.4356 | 132.428 | 0.000462 |
| b0844 | *ybjI* | 120.8905 | 98.25438 | 78.61073 | 184.303 | 479.7632 | 516.3667 | 0.000467 |
| b4416 | *rybA* | 22.89593 | 33.61334 | 43.67263 | 54.23168 | 519.9348 | 250.484 | 0.000481 |
| b0772 | *ybhC* | 108.9846 | 81.44771 | 52.40715 | 184.7267 | 386.7948 | 304.8925 | 0.000482 |
| b3797 | *hisR* | 56.78191 | 62.0554 | 48.03989 | 77.11067 | 728.8269 | 448.6128 | 0.000487 |
| b3049 | *glgS* | 177.6724 | 276.6636 | 202.3498 | 378.3507 | 1585.055 | 1137.444 | 0.00049 |
| b3390 | *aroK* | 239.9493 | 245.6359 | 270.7703 | 452.4956 | 1301.559 | 887.9865 | 0.000527 |
| b3319 | *rplD* | 413.0426 | 773.1068 | 1008.838 | 1116.41 | 7756.555 | 6062.946 | 0.000553 |
| b1798 | *leuE* | 103.4896 | 112.4754 | 74.24347 | 208.453 | 483.2065 | 291.547 | 0.000553 |
| b2537 | *hcaR* | 135.5439 | 104.7185 | 84.43375 | 139.8161 | 1393.379 | 912.6243 | 0.000569 |
| b3255 | *accB* | 139.2073 | 175.8236 | 184.8808 | 322.4243 | 790.8059 | 514.3136 | 0.000578 |
| b3161 | *mtr* | 168.514 | 182.2877 | 190.7038 | 301.6637 | 3336.535 | 829.4718 | 0.000624 |
| b3995 | *rsd* | 102.5738 | 133.1605 | 155.7657 | 131.766 | 2422.919 | 1213.41 | 0.000628 |
| b2113 | *mrp* | 487.2254 | 497.736 | 599.7708 | 924.4807 | 1952.338 | 1561.419 | 0.000676 |
| b0740 | *tolB* | 563.2399 | 645.1176 | 803.5764 | 1064.297 | 4372.962 | 3359.977 | 0.000733 |
| b1747 | *astA* | 113.5638 | 111.1826 | 112.0931 | 92.36333 | 1804.277 | 1373.556 | 0.000733 |
| b3705 | *yidC* | 311.3846 | 288.299 | 368.3058 | 615.6143 | 1111.031 | 758.6382 | 0.000733 |
| b3980 | *tufB* | 25.64344 | 20.68513 | 37.84961 | 49.99483 | 400.5679 | 169.3847 | 0.000736 |
| b4118 | *melR* | 57.69774 | 43.95591 | 37.84961 | 144.4766 | 105.5938 | 126.2686 | 0.000736 |
| b2553 | *glnB* | 84.25702 | 85.32617 | 125.1949 | 140.2397 | 859.6714 | 735.027 | 0.000736 |
| b3961 | *oxyR* | 267.4245 | 341.3047 | 430.9033 | 578.33 | 1956.929 | 1708.219 | 0.000753 |
| b2869 | *ygeV* | 217.0534 | 188.7518 | 174.6905 | 331.3217 | 853.9326 | 722.7081 | 0.000759 |
| b3628 | *waaB* | 136.4597 | 120.2323 | 120.8276 | 228.3662 | 319.077 | 365.4603 | 0.000767 |
| b3363 | *ppiA* | 91.58372 | 111.1826 | 84.43375 | 176.253 | 468.2856 | 340.8226 | 0.000767 |
| b0393 | *rdgC* | 139.2073 | 109.8898 | 123.7391 | 221.5873 | 436.1484 | 415.7625 | 0.000768 |
| b2979 | *glcD* | 124.5539 | 131.8677 | 78.61073 | 202.0977 | 763.2597 | 509.1807 | 0.00079 |
| b2916 | *argP* | 77.84616 | 91.79027 | 69.87621 | 148.7134 | 319.077 | 265.8827 | 0.000801 |
| b1133 | *mnmA* | 444.181 | 500.3216 | 672.5585 | 1279.105 | 1256.796 | 1048.132 | 0.000833 |
| b0954 | *fabA* | 261.0136 | 356.8185 | 372.6731 | 844.8279 | 705.8717 | 646.7416 | 0.000841 |
| b3643 | *rph* | 119.0588 | 116.3539 | 120.8276 | 207.6057 | 546.3333 | 409.603 | 0.000848 |
| b3001 | *gpr* | 153.8606 | 124.1108 | 151.3984 | 254.211 | 789.6581 | 514.3136 | 0.000861 |
| b3554 | *yiaF* | 250.9394 | 232.7077 | 234.3764 | 415.635 | 1076.598 | 754.5319 | 0.00087 |
| b0850 | *ybjC* | 61.36109 | 56.88411 | 56.77442 | 106.7686 | 274.3144 | 216.6071 | 0.000885 |
| b3320 | *rplC* | 681.3829 | 1202.323 | 1583.861 | 1679.487 | 10668.42 | 8663.258 | 0.000897 |
| b2576 | *srmB* | 317.7955 | 308.9842 | 381.4076 | 605.4459 | 946.9011 | 742.213 | 0.000898 |
| b4243 | *ridA* | 489.9729 | 680.0237 | 765.7268 | 993.965 | 4447.566 | 3366.136 | 0.000922 |
| b4381 | *deoC* | 369.0824 | 364.5755 | 381.4076 | 965.5781 | 811.4656 | 628.2633 | 0.000938 |
| b3284 | *smg* | 153.8606 | 186.1662 | 250.3897 | 305.4769 | 1598.828 | 1047.105 | 0.000949 |
| b1908 | *yecA* | 188.6625 | 196.5088 | 235.8322 | 330.4743 | 1094.962 | 912.6243 | 0.000982 |
| b3317 | *rplB* | 494.5521 | 961.8586 | 1279.608 | 1255.379 | 9030.567 | 7770.138 | 0.000986 |
| b1744 | *astE* | 78.762 | 107.3041 | 68.42045 | 107.1923 | 906.7296 | 647.7682 | 0.001013 |
| b3191 | *mlaB* | 73.26697 | 113.7682 | 71.33196 | 149.9845 | 479.7632 | 417.8156 | 0.001019 |
| b3611 | *yibN* | 107.153 | 153.8457 | 160.133 | 203.3688 | 1113.326 | 840.7641 | 0.001041 |
| b3871 | *bipA* | 659.4028 | 753.7145 | 1019.028 | 1399.432 | 3264.226 | 2863.115 | 0.001055 |
| b2330 | *prmB* | 87.00453 | 126.6964 | 133.9294 | 209.3004 | 524.5258 | 425.0016 | 0.001065 |
| b0809 | *glnQ* | 72.35114 | 67.22668 | 52.40715 | 88.97385 | 563.5496 | 435.2674 | 0.00107 |
| b0474 | *adk* | 531.1856 | 610.2114 | 692.939 | 943.9702 | 3008.276 | 2789.201 | 0.001073 |
| b0741 | *pal* | 336.1122 | 418.8739 | 474.5759 | 569.009 | 2987.617 | 2443.246 | 0.001084 |
| b2659 | *csiD* | 126.3855 | 134.4534 | 110.6373 | 104.6502 | 2496.376 | 1018.361 | 0.001088 |
| b1613 | *manA* | 237.2018 | 261.1498 | 266.403 | 443.5982 | 992.8115 | 697.0437 | 0.001125 |
| b4382 | *deoA* | 448.7602 | 504.2001 | 572.1114 | 1160.05 | 1034.131 | 883.8802 | 0.001126 |
| b3164 | *pnp* | 2432.464 | 2796.371 | 3438.492 | 4699.09 | 13113.15 | 10691.77 | 0.00115 |
| b1767 | *ansA* | 81.50951 | 56.88411 | 66.9647 | 153.374 | 211.1876 | 149.8798 | 0.001176 |
| b2150 | *mglB* | 163.9349 | 113.7682 | 88.80101 | 113.1239 | 2061.375 | 1083.036 | 0.001193 |
| b3553 | *ghrB* | 168.514 | 146.0887 | 154.31 | 256.7531 | 664.5524 | 553.3234 | 0.001258 |
| b3865 | *yihA* | 239.9493 | 271.4924 | 286.7836 | 415.635 | 1308.445 | 1070.717 | 0.001271 |
| b3544 | *dppA* | 487.2254 | 537.8134 | 652.1779 | 381.3165 | 12011.3 | 4997.362 | 0.001287 |
| b2695 | *serV* | 14.65339 | 12.92821 | 7.278771 | 24.15005 | 106.7416 | 86.23221 | 0.001303 |
| b2606 | *rplS* | 200.5683 | 321.9124 | 340.6465 | 330.898 | 3141.416 | 2153.752 | 0.001318 |
| b3526 | *kdgK* | 119.9747 | 98.25438 | 115.0046 | 211.8425 | 671.439 | 332.61 | 0.001358 |
| b2840 | *ygeA* | 65.02444 | 78.86207 | 72.78771 | 114.395 | 401.7156 | 340.8226 | 0.001361 |
| b1078 | *flgG* | 44.87602 | 51.71283 | 49.49565 | 95.75281 | 159.5385 | 321.3177 | 0.001402 |
| b3755 | *yieP* | 302.2263 | 360.697 | 375.5846 | 1212.163 | 637.0062 | 780.1962 | 0.001429 |
| b1216 | *chaA* | 108.9846 | 77.56925 | 87.34526 | 155.9161 | 534.8557 | 343.9023 | 0.001566 |
| b1533 | *eamA* | 211.5584 | 166.7739 | 161.5887 | 301.6637 | 903.2863 | 608.7584 | 0.001607 |
| b2187 | *yejL* | 102.5738 | 117.6467 | 155.7657 | 263.9558 | 373.0217 | 263.8295 | 0.001607 |
| b2573 | *rpoE* | 693.2888 | 933.4166 | 879.2756 | 1265.971 | 5783.557 | 3391.8 | 0.001621 |
| b2340 | *sixA* | 329.7014 | 422.7524 | 439.6378 | 254.6347 | 8366.015 | 3464.687 | 0.001621 |
| b3591 | *selA* | 210.6426 | 192.6303 | 235.8322 | 646.5433 | 361.5441 | 518.4199 | 0.001643 |
| b3610 | *grxC* | 68.68779 | 103.4257 | 116.4603 | 166.5082 | 401.7156 | 416.789 | 0.001676 |
| b2620 | *smpB* | 208.8109 | 209.437 | 197.9826 | 355.8954 | 606.0167 | 449.6394 | 0.001695 |
| b0354 | *yaiL* | 117.2272 | 89.20463 | 119.3719 | 196.1662 | 331.7023 | 279.2281 | 0.00171 |
| b2630 | *rnlA* | 391.0625 | 499.0288 | 476.0317 | 838.8963 | 2227.8 | 1177.48 | 0.001712 |
| b0170 | *tsf* | 581.5566 | 757.593 | 882.1871 | 1138.018 | 4086.022 | 2989.383 | 0.001764 |
| b3603 | *lldP* | 176.7566 | 151.26 | 135.3851 | 246.5847 | 720.7926 | 566.6688 | 0.001797 |
| b0440 | *hupB* | 207.895 | 371.0396 | 334.8235 | 332.169 | 3236.68 | 2212.267 | 0.001811 |
| b4242 | *mgtA* | 327.8697 | 337.4262 | 307.1642 | 506.3036 | 1187.93 | 1031.707 | 0.001813 |
| b1928 | *yedD* | 127.3014 | 98.25438 | 187.7923 | 230.9083 | 871.149 | 589.2535 | 0.001813 |
| b1865 | *nudB* | 113.5638 | 129.2821 | 129.5621 | 212.2662 | 433.8529 | 332.61 | 0.001826 |
| b2133 | *dld* | 798.61 | 1022.621 | 1084.537 | 1558.313 | 3024.345 | 2866.195 | 0.001951 |
| b0048 | *folA* | 108.9846 | 102.1328 | 106.2701 | 175.4056 | 472.8767 | 333.6365 | 0.00198 |
| b3741 | *mnmG* | 346.1865 | 343.8903 | 393.0537 | 623.6643 | 1088.075 | 767.8773 | 0.00198 |
| b1204 | *pth* | 64.1086 | 71.10514 | 68.42045 | 116.5134 | 227.2563 | 210.4477 | 0.002006 |
| b2351 | *yfdH* | 79.67784 | 53.00565 | 82.97799 | 137.6976 | 214.6309 | 208.3945 | 0.002017 |
| b2176 | *pdeN* | 108.0688 | 99.5472 | 101.9028 | 200.8267 | 498.1274 | 252.5372 | 0.002039 |
| b2962 | *yggX* | 21.98009 | 24.56359 | 16.0133 | 34.74217 | 171.0161 | 119.0826 | 0.002039 |
| b2241 | *glpA* | 250.0236 | 268.9067 | 151.3984 | 492.7457 | 606.0167 | 492.7555 | 0.002041 |
| b4152 | *frdC* | 61.36109 | 76.27642 | 91.71252 | 438.0903 | 145.7654 | 224.8197 | 0.002041 |
| b0967 | *rlmI* | 206.0634 | 223.658 | 195.0711 | 339.7954 | 576.175 | 513.287 | 0.002054 |
| b2905 | *gcvT* | 321.4589 | 429.2165 | 427.9918 | 385.5534 | 3553.462 | 3181.353 | 0.002055 |
| b3831 | *udp* | 292.1521 | 368.4539 | 374.1289 | 656.7118 | 609.46 | 754.5319 | 0.002058 |
| b1921 | *fliZ* | 38.46516 | 33.61334 | 20.38056 | 76.2633 | 84.93416 | 102.6574 | 0.002065 |
| b4678 | *yoeI* | 6.41086 | 5.171283 | 5.823017 | 7.62633 | 134.2878 | 55.43499 | 0.002065 |
| b1243 | *oppA* | 8973.373 | 11169.97 | 13557.44 | 15015.82 | 94894.41 | 51098.75 | 0.002218 |
| b1247 | *oppF* | 1500.141 | 1625.076 | 1861.91 | 2155.709 | 13002.96 | 7653.109 | 0.002221 |
| b1810 | *yoaC* | 103.4896 | 129.2821 | 149.9427 | 175.4056 | 805.7268 | 651.8745 | 0.002222 |
| b0212 | *gloB* | 119.0588 | 121.5252 | 77.15498 | 213.1136 | 331.7023 | 244.3246 | 0.002329 |
| b1842 | *holE* | 77.84616 | 55.59129 | 66.9647 | 126.2581 | 231.8473 | 171.4379 | 0.002341 |
| b2980 | *glcC* | 177.6724 | 199.0944 | 142.6639 | 157.1871 | 2140.57 | 1223.676 | 0.002375 |
| b3243 | *aaeR* | 273.8353 | 340.0119 | 305.7084 | 517.3194 | 601.4257 | 605.6786 | 0.002383 |
| b4441 | *glmY* | 10.07421 | 12.92821 | 13.10179 | 18.64214 | 156.0952 | 74.9399 | 0.00244 |
| b2069 | *yegD* | 120.8905 | 137.039 | 87.34526 | 275.3953 | 220.3697 | 271.0155 | 0.002444 |
| b4161 | *rsgA* | 551.334 | 729.1509 | 781.7401 | 1334.184 | 1419.778 | 1252.42 | 0.002462 |
| b0281 | *intF* | 272.9195 | 262.4426 | 330.4562 | 452.0719 | 1120.213 | 882.8536 | 0.002473 |
| b0746 | *valZ* | 8.242535 | 6.464104 | 4.367263 | 14.82898 | 58.53571 | 51.3287 | 0.002491 |
| b3750 | *rbsC* | 142.8706 | 104.7185 | 90.25677 | 214.8083 | 284.6442 | 285.3876 | 0.00253 |
| b0536 | *argU* | 10.07421 | 9.049745 | 13.10179 | 26.69216 | 75.75209 | 43.11611 | 0.002623 |
| b0845 | *ybjJ* | 192.3258 | 169.3595 | 123.7391 | 266.9216 | 593.3914 | 528.6856 | 0.0027 |
| b3066 | *dnaG* | 524.7747 | 624.4324 | 656.5452 | 1010.912 | 2688.051 | 1544.994 | 0.002798 |
| b1832 | *msrC* | 81.50951 | 77.56925 | 93.16827 | 124.9871 | 378.7604 | 350.0617 | 0.002866 |
| b1867 | *yecD* | 74.18281 | 94.37592 | 72.78771 | 130.9187 | 286.9397 | 277.175 | 0.00288 |
| b3782 | *rhoL* | 383.7358 | 491.2719 | 550.2751 | 720.6882 | 5894.89 | 1533.702 | 0.003027 |
| b1749 | *xthA* | 85.17286 | 53.00565 | 48.03989 | 113.9713 | 246.7682 | 217.6337 | 0.003153 |
| b4234 | *yjgA* | 289.4046 | 281.8349 | 308.6199 | 448.6824 | 1154.645 | 839.7375 | 0.003154 |
| b3732 | *atpD* | 639.2544 | 740.7863 | 924.404 | 829.1516 | 6374.653 | 4402.976 | 0.003154 |
| b2844 | *yqeF* | 123.638 | 108.5969 | 81.52224 | 167.7793 | 461.3991 | 359.3009 | 0.003221 |
| b3426 | *glpD* | 410.2951 | 835.1622 | 246.0225 | 379.6218 | 13775.4 | 2572.594 | 0.003283 |
| b0990 | *cspG* | 340.6914 | 236.5862 | 213.9959 | 274.9716 | 3854.174 | 1242.155 | 0.003346 |
| b2231 | *gyrA* | 547.6706 | 700.7089 | 818.1339 | 1070.652 | 3343.422 | 2135.274 | 0.003407 |
| b2268 | *rbn* | 71.4353 | 60.76258 | 62.59743 | 119.4792 | 203.1533 | 146.8001 | 0.003513 |
| b2279 | *nuoK* | 30.22263 | 28.44206 | 24.74782 | 67.36592 | 64.2745 | 70.8336 | 0.003595 |
| b1324 | *tpx* | 324.2064 | 386.5534 | 465.8414 | 496.1351 | 2579.014 | 1753.388 | 0.003633 |
| b2251 | *nudI* | 46.7077 | 33.61334 | 39.30537 | 58.46853 | 239.8816 | 185.8099 | 0.003748 |
| b2292 | *yfbS* | 406.6317 | 371.0396 | 304.2526 | 958.3755 | 558.9586 | 1072.77 | 0.003748 |
| b0666 | *metU* | 9.158372 | 11.63539 | 5.823017 | 16.52372 | 81.49088 | 59.54129 | 0.00379 |
| b2296 | *ackA* | 525.6905 | 646.4104 | 576.4787 | 1750.666 | 1404.857 | 898.2522 | 0.003836 |
| b1487 | *ddpA* | 97.07874 | 78.86207 | 84.43375 | 118.2081 | 608.3122 | 350.0617 | 0.003917 |
| b2937 | *speB* | 105.3213 | 111.1826 | 104.8143 | 140.6634 | 532.5601 | 493.7821 | 0.003932 |
| b4137 | *cutA* | 54.95023 | 63.34822 | 65.50894 | 104.6502 | 236.4383 | 166.305 | 0.003932 |
| b0897 | *ycaC* | 82.42535 | 77.56925 | 52.40715 | 93.63439 | 659.9614 | 306.9456 | 0.003956 |
| b0835 | *rimO* | 108.9846 | 124.1108 | 93.16827 | 247.0084 | 183.6414 | 268.9624 | 0.004009 |
| b4330 | *yjiH* | 90.66788 | 102.1328 | 68.42045 | 215.6557 | 204.3011 | 157.0658 | 0.00408 |
| b2280 | *nuoJ* | 85.17286 | 115.061 | 96.07978 | 172.8635 | 383.3515 | 239.1917 | 0.004271 |
| b2159 | *nfo* | 119.9747 | 157.7241 | 202.3498 | 238.111 | 842.4551 | 581.0409 | 0.004294 |
| b1724 | *ydiZ* | 137.3756 | 179.7021 | 132.4736 | 222.8583 | 612.9033 | 503.0212 | 0.00431 |
| b3040 | *zupT* | 160.2715 | 173.238 | 152.8542 | 242.7715 | 591.0959 | 473.2506 | 0.00431 |
| b3649 | *rpoZ* | 456.0869 | 499.0288 | 592.492 | 655.017 | 3303.25 | 2049.042 | 0.004353 |
| b0145 | *dksA* | 308.6371 | 464.1227 | 487.6777 | 349.5401 | 4447.566 | 2764.564 | 0.00441 |
| b3863 | *polA* | 435.0227 | 395.6032 | 452.7396 | 621.5459 | 1717.047 | 1249.341 | 0.004414 |
| b1057 | *yceJ* | 41.21267 | 36.19898 | 36.39386 | 99.56598 | 94.11623 | 71.86018 | 0.004441 |
| b1825 | *yebO* | 133.7122 | 197.8016 | 157.2215 | 194.0477 | 2308.143 | 580.0143 | 0.004496 |
| b0665 | *glnV* | 7.326697 | 9.049745 | 7.278771 | 20.33688 | 59.68346 | 31.82379 | 0.004608 |
| b3605 | *lldD* | 169.4299 | 131.8677 | 171.779 | 241.0768 | 527.9691 | 460.9317 | 0.004636 |
| b1190 | *dadX* | 439.6018 | 458.9514 | 471.6644 | 297.0032 | 5016.854 | 3623.806 | 0.004669 |
| b3981 | *secE* | 259.1819 | 324.498 | 368.3058 | 462.664 | 1385.345 | 1011.175 | 0.004696 |
| b0418 | *pgpA* | 386.4833 | 460.2442 | 368.3058 | 585.109 | 1471.427 | 1218.543 | 0.00472 |
| b2696 | *csrA* | 769.3032 | 1153.196 | 1275.241 | 1125.307 | 9053.522 | 5445.975 | 0.004772 |
| b1034 | *ycdX* | 238.1177 | 245.6359 | 288.2393 | 385.1297 | 922.7982 | 703.2032 | 0.004792 |
| b1662 | *ribC* | 258.2661 | 252.1 | 292.6066 | 350.8112 | 1298.115 | 1015.282 | 0.004809 |
| b4315 | *fimI* | 151.1131 | 159.017 | 196.5268 | 275.3953 | 410.8977 | 654.9542 | 0.005208 |
| b1343 | *dbpA* | 172.1774 | 165.4811 | 132.4736 | 282.1742 | 297.2696 | 295.6533 | 0.005246 |
| b0494 | *tesA* | 78.762 | 81.44771 | 115.0046 | 138.1213 | 480.911 | 304.8925 | 0.005274 |
| b1534 | *ydeE* | 93.41539 | 74.9836 | 78.61073 | 147.0187 | 219.222 | 168.3581 | 0.005356 |
| b1814 | *sdaA* | 326.9539 | 323.2052 | 407.6112 | 399.1113 | 2047.602 | 1708.219 | 0.005366 |
| b3702 | *dnaA* | 821.506 | 1118.29 | 1167.515 | 1774.816 | 3284.886 | 2116.796 | 0.005538 |
| b4153 | *frdB* | 139.2073 | 164.1882 | 222.7304 | 1004.981 | 282.3487 | 492.7555 | 0.005546 |
| b3604 | *lldR* | 43.96018 | 41.37026 | 36.39386 | 54.65537 | 265.1323 | 173.491 | 0.005592 |
| b1244 | *oppB* | 1321.553 | 1394.954 | 1655.193 | 1708.722 | 10062.4 | 5753.947 | 0.005632 |
| b0160 | *dgt* | 166.6824 | 121.5252 | 115.0046 | 202.0977 | 570.4362 | 421.9219 | 0.005658 |
| b0463 | *acrA* | 337.0281 | 448.6088 | 465.8414 | 459.2745 | 2894.648 | 1899.162 | 0.005866 |
| b0118 | *acnB* | 3379.439 | 3742.716 | 4808.356 | 4047.039 | 33783.14 | 18276.1 | 0.005881 |
| b4039 | *ubiC* | 187.7466 | 162.8954 | 213.9959 | 178.3714 | 1762.958 | 937.262 | 0.0059 |
| b3633 | *waaA* | 185.9149 | 209.437 | 215.4516 | 330.898 | 407.4544 | 365.4603 | 0.005926 |
| b3625 | *waaY* | 43.04435 | 51.71283 | 59.68593 | 86.85543 | 145.7654 | 153.9861 | 0.005939 |
| b1271 | *yciK* | 80.59367 | 130.5749 | 96.07978 | 152.5266 | 533.7079 | 340.8226 | 0.00594 |
| b0604 | *dsbG* | 88.83621 | 87.91181 | 55.31866 | 161.8477 | 164.1295 | 172.4644 | 0.006028 |
| b1332 | *ynaJ* | 252.7711 | 382.6749 | 331.912 | 214.3846 | 3706.114 | 2198.921 | 0.006028 |
| b1601 | *tqsA* | 185.9149 | 157.7241 | 126.6506 | 311.4085 | 324.8158 | 282.3078 | 0.006061 |
| b0384 | *psiF* | 59.52942 | 64.64104 | 55.31866 | 74.56856 | 385.647 | 254.5903 | 0.006109 |
| b0380 | *yaiZ* | 85.17286 | 50.42001 | 80.06649 | 119.4792 | 278.9054 | 216.6071 | 0.006324 |
| b3315 | *rplV* | 374.5774 | 620.554 | 786.1073 | 577.059 | 4565.785 | 3621.753 | 0.006343 |
| b2795 | *ppnN* | 373.6616 | 466.7083 | 582.3017 | 765.5988 | 1401.414 | 1100.487 | 0.006375 |
| b1824 | *yobF* | 160.2715 | 324.498 | 342.1023 | 123.716 | 3823.185 | 2640.348 | 0.006509 |
| b3963 | *fabR* | 315.048 | 364.5755 | 337.735 | 497.8299 | 1438.142 | 881.827 | 0.006534 |
| b0655 | *gltI* | 483.562 | 500.3216 | 522.6158 | 598.6669 | 3244.715 | 1811.903 | 0.006731 |
| b4232 | *fbp* | 341.6073 | 377.5037 | 407.6112 | 494.8641 | 1537.997 | 1238.048 | 0.006731 |
| b1538 | *dcp* | 345.2706 | 383.9678 | 413.4342 | 577.9063 | 956.0832 | 782.2494 | 0.0068 |
| b3954 | *yijO* | 55.86607 | 62.0554 | 56.77442 | 101.2607 | 160.6863 | 125.242 | 0.006853 |
| b4501 | *torI* | 20.14842 | 19.39231 | 14.55754 | 41.09745 | 193.9713 | 43.11611 | 0.006956 |
| b0316 | *yahB* | 88.83621 | 87.91181 | 68.42045 | 151.6792 | 204.3011 | 153.9861 | 0.007069 |
| b4213 | *cpdB* | 195.0733 | 191.3375 | 176.1463 | 289.3769 | 370.7261 | 413.7093 | 0.007105 |
| b0331 | *prpB* | 57.69774 | 71.10514 | 45.12838 | 92.78702 | 490.0931 | 164.2518 | 0.007153 |
| b0680 | *glnS* | 501.8788 | 536.5206 | 615.7841 | 722.3829 | 2279.449 | 1770.84 | 0.007265 |
| b3177 | *folP* | 122.7222 | 135.7462 | 147.0312 | 184.303 | 494.6841 | 450.666 | 0.007306 |
| b0143 | *pcnB* | 282.0779 | 311.5698 | 393.0537 | 513.9299 | 943.4578 | 729.8941 | 0.007323 |
| b3009 | *yghB* | 816.9268 | 801.5489 | 978.2669 | 1263.852 | 2091.217 | 1935.092 | 0.007338 |
| b1637 | *tyrS* | 395.6417 | 455.0729 | 470.2086 | 642.7302 | 1334.844 | 1033.76 | 0.007355 |
| b3311 | *rpsQ* | 76.01449 | 173.238 | 154.31 | 94.48176 | 1455.358 | 983.4579 | 0.007379 |
| b3382 | *yhfY* | 28.39095 | 37.4918 | 17.46905 | 57.19748 | 83.7864 | 97.52453 | 0.007391 |
| b4255 | *rraB* | 501.8788 | 654.1673 | 634.7089 | 783.8173 | 2343.724 | 1980.261 | 0.007391 |
| b0736 | *ybgC* | 151.1131 | 175.8236 | 158.6772 | 233.0268 | 749.4866 | 446.5597 | 0.00741 |
| b4750 | *ynfS* | 70.51946 | 71.10514 | 106.2701 | 119.9029 | 617.4943 | 255.6169 | 0.007413 |
| b1123 | *potD* | 170.3457 | 204.2657 | 230.0092 | 260.99 | 999.698 | 692.9374 | 0.007439 |
| b2498 | *upp* | 82.42535 | 117.6467 | 91.71252 | 159.3056 | 230.6995 | 259.7232 | 0.007463 |
| b3496 | *dtpB* | 239.9493 | 285.7134 | 285.3278 | 591.0406 | 401.7156 | 672.406 | 0.007463 |
| b1604 | *ydgH* | 77.84616 | 63.34822 | 59.68593 | 95.32913 | 363.8396 | 217.6337 | 0.00752 |
| b1087 | *yceF* | 154.7765 | 179.7021 | 173.2348 | 228.3662 | 789.6581 | 519.4464 | 0.007538 |
| b3189 | *murA* | 267.4245 | 299.9344 | 289.6951 | 423.2613 | 684.0643 | 597.4661 | 0.007625 |
| b2536 | *hcaT* | 214.3059 | 246.9288 | 205.2614 | 352.9296 | 539.4467 | 420.8953 | 0.007631 |
| b1071 | *flgM* | 40.29684 | 67.22668 | 59.68593 | 91.93965 | 213.4832 | 163.2253 | 0.007637 |
| b1876 | *argS* | 271.0878 | 299.9344 | 375.5846 | 439.3614 | 1067.416 | 961.8998 | 0.007646 |
| b3984 | *rplA* | 814.1793 | 1251.45 | 1548.923 | 1462.984 | 6241.513 | 5346.397 | 0.007657 |
| b1916 | *sdiA* | 85.17286 | 108.5969 | 107.7258 | 120.3265 | 661.1091 | 382.9121 | 0.00775 |
| b3084 | *rlmG* | 153.8606 | 184.8734 | 216.9074 | 288.5295 | 468.2856 | 427.0548 | 0.007758 |
| b3358 | *yhfK* | 257.3502 | 222.3652 | 276.5933 | 451.2245 | 480.911 | 404.4701 | 0.007758 |
| b1610 | *tus* | 106.2371 | 107.3041 | 96.07978 | 148.7134 | 563.5496 | 292.5736 | 0.007802 |
| b0002 | *thrA* | 347.1023 | 316.7411 | 372.6731 | 449.5298 | 1650.477 | 1062.504 | 0.008075 |
| b4453 | *ldrD* | 54.95023 | 69.81232 | 49.49565 | 76.68699 | 247.9159 | 256.6435 | 0.008075 |
| b4439 | *micF* | 10.07421 | 3.878462 | 4.367263 | 28.3869 | 35.58053 | 19.50491 | 0.008081 |
| b1740 | *nadE* | 182.2516 | 219.7795 | 289.6951 | 292.7663 | 1046.756 | 892.0928 | 0.008082 |
| b1059 | *solA* | 156.6082 | 102.1328 | 129.5621 | 194.8951 | 507.3094 | 361.354 | 0.00811 |
| b2912 | *fau* | 388.315 | 478.3437 | 455.6511 | 605.4459 | 1226.954 | 2128.088 | 0.00811 |
| b4523 | *yciX* | 42.12851 | 55.59129 | 49.49565 | 109.3107 | 104.4461 | 91.36508 | 0.008119 |
| b3626 | *waaJ* | 69.60363 | 102.1328 | 110.6373 | 177.1003 | 187.0847 | 249.4575 | 0.008133 |
| b1702 | *ppsA* | 593.4625 | 819.6484 | 988.4572 | 1115.563 | 2896.944 | 2608.524 | 0.008165 |
| b0121 | *speE* | 141.9548 | 139.6246 | 152.8542 | 202.9451 | 548.6288 | 402.417 | 0.008169 |
| b1262 | *trpC* | 208.8109 | 201.68 | 183.425 | 301.24 | 491.2408 | 402.417 | 0.008169 |
| b3964 | *yijD* | 128.2172 | 125.4036 | 145.5754 | 183.0319 | 618.6421 | 391.1247 | 0.00823 |
| b0782 | *moaB* | 109.9005 | 89.20463 | 94.62403 | 157.6108 | 305.3039 | 216.6071 | 0.008249 |
| b2976 | *glcB* | 460.6661 | 302.5201 | 410.5227 | 420.7192 | 2249.608 | 1786.239 | 0.008249 |
| b1260 | *trpA* | 162.1032 | 177.1164 | 171.779 | 235.5689 | 720.7926 | 476.3303 | 0.008253 |
| b4391 | *ettA* | 407.5475 | 361.9898 | 490.5892 | 399.1113 | 3026.64 | 1978.208 | 0.008253 |
| b0444 | *queC* | 92.49956 | 107.3041 | 100.447 | 166.9319 | 193.9713 | 195.0491 | 0.008253 |
| b0526 | *cysS* | 133.7122 | 131.8677 | 139.7524 | 191.0819 | 455.6603 | 366.4869 | 0.00826 |
| b0161 | *degP* | 182.2516 | 196.5088 | 171.779 | 203.3688 | 914.7639 | 805.8606 | 0.008424 |
| b3342 | *rpsL* | 1234.549 | 1455.716 | 1611.52 | 1655.761 | 6859.007 | 5535.287 | 0.008438 |
| b3314 | *rpsC* | 648.4127 | 1219.13 | 1477.591 | 1054.976 | 8236.318 | 6417.114 | 0.008499 |
| b0914 | *msbA* | 411.2109 | 521.0068 | 513.8813 | 762.2093 | 1255.648 | 909.5445 | 0.008507 |
| b2393 | *nupC* | 121.8063 | 109.8898 | 91.71252 | 155.4924 | 395.9768 | 307.9722 | 0.008573 |
| b3592 | *yibF* | 65.94028 | 55.59129 | 50.9514 | 88.97385 | 319.077 | 158.0924 | 0.00874 |
| b3203 | *hpf* | 207.895 | 292.1775 | 369.7616 | 363.9454 | 1519.633 | 1087.142 | 0.00874 |
| b2697 | *alaS* | 930.4906 | 1126.047 | 1224.289 | 1349.86 | 5110.971 | 3621.753 | 0.00874 |
| b0178 | *skp* | 668.5611 | 806.7202 | 871.9968 | 768.9883 | 4442.975 | 3832.201 | 0.00874 |
| b2124 | *yehS* | 191.41 | 213.3154 | 228.5534 | 355.048 | 650.7793 | 388.045 | 0.008931 |
| b2172 | *yeiQ* | 216.1376 | 204.2657 | 209.6286 | 1022.352 | 485.502 | 319.2645 | 0.009052 |
| b0433 | *ampG* | 173.0932 | 148.6744 | 120.8276 | 258.0242 | 314.4859 | 273.0687 | 0.009238 |
| b1422 | *ydcI* | 359.0082 | 302.5201 | 331.912 | 336.4059 | 2326.507 | 1314.015 | 0.009348 |
| b1738 | *chbB* | 68.68779 | 50.42001 | 56.77442 | 82.61858 | 294.974 | 185.8099 | 0.009562 |
| b3925 | *glpX* | 259.1819 | 332.2549 | 324.6332 | 447.4114 | 804.579 | 685.7514 | 0.009666 |
| b3233 | *yhcB* | 596.21 | 778.2781 | 720.5984 | 872.3674 | 3080.585 | 2199.948 | 0.009802 |
| b4208 | *cycA* | 353.5132 | 359.4042 | 410.5227 | 422.8376 | 2604.265 | 1237.022 | 0.00998 |
| b1246 | *oppD* | 1765.734 | 1848.734 | 2343.764 | 2206.128 | 11635.98 | 7126.477 | 0.010018 |
| b1591 | *dmsD* | 154.7765 | 152.5528 | 142.6639 | 224.5531 | 322.5203 | 312.0785 | 0.010025 |
| b2299 | *yfcD* | 193.2416 | 227.5365 | 238.7437 | 274.9716 | 1097.258 | 688.8311 | 0.010025 |
| b4164 | *glyX* | 0.915837 | 0 | 0 | 8.050015 | 21.80742 | 5.13287 | 0.010025 |
| b3885 | *yihX* | 138.2914 | 137.039 | 154.31 | 141.5108 | 879.1833 | 645.715 | 0.010026 |
| b0333 | *prpC* | 65.02444 | 60.76258 | 49.49565 | 90.24491 | 385.647 | 151.9329 | 0.010052 |
| b1770 | *ydjF* | 71.4353 | 99.5472 | 66.9647 | 146.595 | 273.1666 | 158.0924 | 0.010067 |
| b0387 | *yaiI* | 156.6082 | 183.5805 | 149.9427 | 271.5821 | 328.2591 | 279.2281 | 0.010116 |
| b3348 | *slyX* | 69.60363 | 69.81232 | 96.07978 | 120.3265 | 229.5518 | 209.4211 | 0.010208 |
| b2157 | *yeiE* | 213.3901 | 236.5862 | 232.9207 | 338.5243 | 863.1147 | 506.101 | 0.010223 |
| b3908 | *sodA* | 777.5458 | 702.0017 | 879.2756 | 183.0319 | 12250.03 | 7367.721 | 0.010231 |
| b0896 | *dmsC* | 132.7964 | 120.2323 | 138.2967 | 231.7557 | 210.0399 | 255.6169 | 0.010358 |
| b2873 | *hyuA* | 117.2272 | 90.49745 | 109.1816 | 253.3636 | 157.243 | 282.3078 | 0.010358 |
| b1233 | *ychJ* | 109.9005 | 87.91181 | 100.447 | 153.7977 | 446.4782 | 234.0589 | 0.010478 |
| b3601 | *mtlR* | 74.18281 | 62.0554 | 84.43375 | 111.4292 | 309.8949 | 188.8896 | 0.010656 |
| b2834 | *tas* | 217.0534 | 276.6636 | 256.2128 | 349.9638 | 716.2016 | 624.157 | 0.010725 |
| b4154 | *frdA* | 549.5023 | 734.3222 | 853.072 | 4124.574 | 1080.041 | 1653.811 | 0.011134 |
| b3065 | *rpsU* | 397.4733 | 711.0514 | 780.2843 | 705.4355 | 4203.093 | 2424.768 | 0.011151 |
| b0146 | *sfsA* | 85.17286 | 71.10514 | 59.68593 | 94.48176 | 301.8606 | 253.5638 | 0.011163 |
| b1125 | *potB* | 72.35114 | 73.69078 | 66.9647 | 117.3607 | 173.3116 | 138.5875 | 0.011322 |
| b1070 | *flgN* | 33.88598 | 34.90616 | 50.9514 | 59.73959 | 173.3116 | 126.2686 | 0.011322 |
| b0196 | *rcsF* | 148.3656 | 140.9175 | 213.9959 | 254.211 | 584.2093 | 408.5764 | 0.011343 |
| b3051 | *yqiK* | 159.3557 | 95.66874 | 82.97799 | 258.0242 | 249.0637 | 235.0854 | 0.011368 |
| b1512 | *lsrR* | 97.07874 | 91.79027 | 91.71252 | 153.374 | 172.1638 | 234.0589 | 0.011429 |
| b4512 | *ybdD* | 79.67784 | 107.3041 | 126.6506 | 91.51596 | 812.6133 | 501.9947 | 0.011442 |
| b3312 | *rpmC* | 241.781 | 439.5591 | 553.1866 | 363.9454 | 2682.313 | 2485.336 | 0.01145 |
| b0382 | *iraP* | 176.7566 | 234.0006 | 241.6552 | 78.38173 | 2535.399 | 1833.461 | 0.011456 |
| b2267 | *elaA* | 107.153 | 93.08309 | 87.34526 | 170.3214 | 169.8683 | 181.7036 | 0.011459 |
| b1284 | *yciT* | 81.50951 | 51.71283 | 58.23017 | 103.8028 | 258.2458 | 160.1455 | 0.011502 |
| b3919 | *tpiA* | 448.7602 | 595.9904 | 608.5053 | 811.3568 | 1394.527 | 1171.321 | 0.011513 |
| b1511 | *lsrK* | 198.7367 | 196.5088 | 206.7171 | 272.4295 | 506.1617 | 558.4562 | 0.011997 |
| b1299 | *puuR* | 58.61358 | 51.71283 | 72.78771 | 89.39754 | 387.9425 | 160.1455 | 0.012039 |
| b4219 | *msrA* | 69.60363 | 115.061 | 107.7258 | 139.8161 | 347.771 | 295.6533 | 0.012142 |
| b1807 | *tsaB* | 112.648 | 73.69078 | 90.25677 | 169.0503 | 226.1085 | 171.4379 | 0.012205 |
| b3821 | *pldA* | 152.9448 | 137.039 | 165.956 | 221.1636 | 375.3172 | 333.6365 | 0.012308 |
| b3999 | *yjaG* | 101.6579 | 121.5252 | 98.99129 | 163.9661 | 340.8844 | 232.0057 | 0.012499 |
| b1298 | *puuD* | 55.86607 | 42.66308 | 52.40715 | 66.94223 | 500.4229 | 128.3217 | 0.012528 |
| b0127 | *yadG* | 177.6724 | 192.6303 | 247.4782 | 318.6111 | 593.3914 | 419.8688 | 0.012623 |
| b0598 | *cstA* | 722.5955 | 762.7642 | 873.4526 | 335.9822 | 9394.407 | 4927.555 | 0.012664 |
| b3924 | *fpr* | 359.0082 | 359.4042 | 441.0935 | 386.8244 | 2503.262 | 1409.486 | 0.012811 |
| b1817 | *manX* | 196.905 | 212.0226 | 260.58 | 312.2558 | 767.8507 | 539.9779 | 0.012819 |
| b1426 | *ydcH* | 36.63349 | 27.14924 | 30.57084 | 47.45272 | 157.243 | 94.44481 | 0.012819 |
| b2239 | *glpQ* | 185.9149 | 245.6359 | 215.4516 | 233.0268 | 954.9354 | 880.8005 | 0.012819 |
| b1587 | *ynfE* | 192.3258 | 177.1164 | 181.9693 | 567.7379 | 257.098 | 411.6562 | 0.01301 |
| b0672 | *leuW* | 27.47512 | 64.64104 | 43.67263 | 61.43433 | 237.5861 | 183.7567 | 0.013196 |
| b0117 | *yacH* | 135.5439 | 138.3318 | 104.8143 | 162.695 | 529.1169 | 374.6995 | 0.013212 |
| b1261 | *trpB* | 192.3258 | 188.7518 | 142.6639 | 227.9425 | 583.0615 | 526.6324 | 0.013312 |
| b3064 | *tsaD* | 250.0236 | 310.277 | 259.1243 | 373.6902 | 788.5104 | 657.0073 | 0.013374 |
| b0958 | *sulA* | 190.4941 | 221.0723 | 200.8941 | 301.6637 | 829.8297 | 438.3471 | 0.013401 |
| b4044 | *dinF* | 142.8706 | 112.4754 | 145.5754 | 213.1136 | 323.668 | 250.484 | 0.013494 |
| b0889 | *lrp* | 165.7665 | 215.9011 | 285.3278 | 271.5821 | 1223.511 | 704.2297 | 0.013531 |
| b0843 | *ybjH* | 44.87602 | 38.78462 | 26.20358 | 39.82639 | 266.2801 | 153.9861 | 0.013617 |
| b1945 | *fliM* | 60.44525 | 67.22668 | 42.21687 | 105.4976 | 112.4804 | 195.0491 | 0.013821 |
| b3441 | *yhhY* | 55.86607 | 45.24873 | 27.65933 | 73.29751 | 183.6414 | 116.0029 | 0.013885 |
| b1124 | *potC* | 69.60363 | 78.86207 | 81.52224 | 122.445 | 150.3564 | 175.5441 | 0.013885 |
| b4380 | *yjjI* | 119.9747 | 112.4754 | 85.8895 | 860.5042 | 149.2087 | 243.298 | 0.013895 |
| b0232 | *yafN* | 56.78191 | 47.83437 | 59.68593 | 77.53436 | 192.8235 | 155.0127 | 0.014239 |
| b2572 | *rseA* | 1017.495 | 1296.699 | 1394.613 | 1454.087 | 5498.913 | 3768.553 | 0.014321 |
| b0670 | *glnU* | 41.21267 | 49.12719 | 36.39386 | 41.94482 | 268.5756 | 190.9428 | 0.014496 |
| b3527 | *yhjJ* | 157.524 | 168.0667 | 168.8675 | 223.282 | 513.0482 | 393.1778 | 0.014679 |
| b1643 | *ydhI* | 31.13846 | 31.0277 | 17.46905 | 52.96063 | 76.89985 | 67.75388 | 0.014692 |
| b1245 | *oppC* | 1299.573 | 1441.495 | 1682.852 | 1483.321 | 8371.754 | 5210.889 | 0.014716 |
| b2321 | *flk* | 145.6181 | 188.7518 | 163.0445 | 227.5188 | 475.1722 | 411.6562 | 0.014721 |
| b2513 | *yfgM* | 271.0878 | 332.2549 | 334.8235 | 438.0903 | 790.8059 | 671.3794 | 0.014721 |
| b3306 | *rpsH* | 1043.139 | 1577.241 | 2081.729 | 1947.256 | 6778.664 | 5375.141 | 0.01476 |
| b1187 | *fadR* | 377.3249 | 391.7247 | 521.16 | 419.8718 | 2620.334 | 1603.509 | 0.014808 |
| b4023 | *yjbD* | 139.2073 | 182.2877 | 129.5621 | 209.3004 | 609.46 | 373.6729 | 0.014808 |
| b3556 | *cspA* | 2220.905 | 1886.225 | 2013.308 | 2043.009 | 8686.24 | 8445.624 | 0.014862 |
| b0162 | *cdaR* | 130.9647 | 78.86207 | 88.80101 | 188.9635 | 199.7101 | 209.4211 | 0.014921 |
| b3160 | *yhbW* | 151.1131 | 109.8898 | 116.4603 | 185.1503 | 383.3515 | 285.3876 | 0.015394 |
| b3555 | *yiaG* | 235.3702 | 308.9842 | 266.403 | 280.4795 | 1572.43 | 898.2522 | 0.016141 |
| b2903 | *gcvP* | 497.2996 | 606.3329 | 690.0275 | 433.4298 | 3830.072 | 3177.246 | 0.016145 |
| b2948 | *yqgE* | 408.4634 | 514.5427 | 567.7442 | 815.5936 | 1185.635 | 818.1795 | 0.016524 |
| b2216 | *rcsD* | 591.6308 | 570.134 | 745.3462 | 875.3332 | 1702.126 | 1380.742 | 0.016524 |
| b3891 | *fdhE* | 299.4788 | 332.2549 | 378.4961 | 441.4798 | 1316.479 | 813.0466 | 0.016524 |
| b2149 | *mglA* | 101.6579 | 78.86207 | 77.15498 | 143.2055 | 238.7339 | 161.1721 | 0.016627 |
| b2895 | *fldB* | 229.8751 | 314.1554 | 282.4163 | 309.7137 | 1451.915 | 833.5781 | 0.016878 |
| b2690 | *yqaB* | 140.1231 | 137.039 | 163.0445 | 214.8083 | 515.3438 | 302.8393 | 0.017328 |
| b1219 | *ychN* | 166.6824 | 215.9011 | 196.5268 | 195.3188 | 856.2282 | 767.8773 | 0.017478 |
| b0222 | *lpcA* | 178.5883 | 169.3595 | 189.2481 | 198.7083 | 897.5475 | 546.1374 | 0.017478 |
| b0420 | *dxs* | 475.3195 | 510.6642 | 625.9743 | 712.2145 | 1433.551 | 1283.217 | 0.017522 |
| b4558 | *yifL* | 134.6281 | 151.26 | 203.8056 | 204.6399 | 759.8164 | 453.7457 | 0.017678 |
| b1189 | *dadA* | 794.0308 | 961.8586 | 1029.218 | 413.9403 | 8093.996 | 6168.683 | 0.01776 |
| b0383 | *phoA* | 127.3014 | 127.9893 | 168.8675 | 127.1055 | 967.5608 | 530.7387 | 0.017793 |
| b2967 | *pheV* | 11.90588 | 12.92821 | 8.734526 | 24.99742 | 52.79691 | 29.77065 | 0.018081 |
| b0783 | *moaC* | 42.12851 | 45.24873 | 48.03989 | 57.62116 | 183.6414 | 142.6938 | 0.018189 |
| b3470 | *tusA* | 225.2959 | 249.5144 | 295.5181 | 673.2355 | 540.5945 | 357.2477 | 0.018247 |
| b1647 | *ydhF* | 168.514 | 204.2657 | 181.9693 | 232.6031 | 604.869 | 493.7821 | 0.018267 |
| b1276 | *acnA* | 1388.409 | 1599.219 | 1981.282 | 1729.059 | 7831.159 | 5589.695 | 0.018276 |
| b2631 | *rnlB* | 80.59367 | 103.4257 | 113.5488 | 169.474 | 531.4124 | 182.7302 | 0.018284 |
| b3035 | *tolC* | 603.5367 | 685.195 | 822.5012 | 774.0725 | 3017.458 | 2227.666 | 0.018415 |
| b4329 | *yjiG* | 57.69774 | 46.54155 | 32.02659 | 112.7002 | 81.49088 | 105.7371 | 0.01855 |
| b0346 | *mhpR* | 68.68779 | 78.86207 | 61.14168 | 80.50015 | 285.792 | 244.3246 | 0.018634 |
| b2828 | *lgt* | 184.0833 | 140.9175 | 168.8675 | 250.3978 | 332.8501 | 303.8659 | 0.018679 |
| b0056 | *yabP* | 98.91042 | 64.64104 | 119.3719 | 146.1713 | 303.0084 | 225.8463 | 0.018967 |
| b4034 | *malE* | 122.7222 | 125.4036 | 88.80101 | 175.8293 | 339.7366 | 221.74 | 0.019299 |
| b2514 | *hisS* | 674.972 | 864.8971 | 882.1871 | 1099.463 | 2158.935 | 1701.033 | 0.019303 |
| b2585 | *pssA* | 369.0824 | 403.3601 | 487.6777 | 593.5827 | 1220.068 | 840.7641 | 0.019327 |
| b0762 | *acrZ* | 253.6869 | 237.879 | 177.602 | 287.2584 | 906.7296 | 563.5891 | 0.019428 |
| b1094 | *acpP* | 1670.487 | 2383.961 | 2854.734 | 1742.193 | 15301.92 | 10512.12 | 0.019538 |
| b2805 | *fucR* | 100.7421 | 77.56925 | 69.87621 | 105.0739 | 307.5994 | 247.4043 | 0.019889 |
| b2480 | *bcp* | 109.9005 | 138.3318 | 120.8276 | 141.0871 | 556.6631 | 366.4869 | 0.019889 |
| b1583 | *ynfB* | 157.524 | 161.6026 | 195.0711 | 278.7847 | 328.2591 | 273.0687 | 0.019893 |
| b3183 | *obgE* | 354.429 | 390.4319 | 508.0582 | 478.3404 | 1469.131 | 1343.785 | 0.020499 |
| b4364 | *yjjP* | 54.95023 | 56.88411 | 84.43375 | 133.0371 | 123.958 | 122.1623 | 0.020725 |
| b0969 | *tusE* | 97.99458 | 94.37592 | 82.97799 | 105.4976 | 333.9978 | 310.0253 | 0.020725 |
| b2699 | *recA* | 601.705 | 672.2668 | 842.8817 | 783.8173 | 3676.272 | 1965.889 | 0.02077 |
| b3997 | *hemE* | 211.5584 | 253.3929 | 244.5667 | 311.8322 | 686.3598 | 527.659 | 0.02097 |
| b1143 | *ymfI* | 42.12851 | 62.0554 | 64.05319 | 86.43174 | 158.3907 | 132.428 | 0.020982 |
| b1866 | *aspS* | 623.6851 | 786.035 | 901.1119 | 1079.973 | 1715.9 | 1611.721 | 0.021029 |
| b2217 | *rcsB* | 417.6218 | 447.316 | 515.337 | 542.3168 | 1805.425 | 1208.278 | 0.021069 |
| b1423 | *ydcJ* | 210.6426 | 188.7518 | 202.3498 | 236.8399 | 950.3444 | 517.3933 | 0.021096 |
| b2008 | *yeeA* | 149.2815 | 127.9893 | 160.133 | 183.8793 | 539.4467 | 367.5135 | 0.021293 |
| b2865 | *ygeR* | 242.6969 | 277.9565 | 358.1156 | 420.2955 | 1069.711 | 605.6786 | 0.021419 |
| b0780 | *ybhK* | 139.2073 | 122.818 | 128.1064 | 174.5582 | 393.6813 | 290.5204 | 0.021708 |
| b3602 | *yibL* | 92.49956 | 125.4036 | 123.7391 | 164.8135 | 527.9691 | 239.1917 | 0.021754 |
| b1752 | *ydjZ* | 57.69774 | 55.59129 | 69.87621 | 133.8845 | 98.70727 | 114.9763 | 0.021769 |
| b0345 | *lacI* | 133.7122 | 117.6467 | 112.0931 | 152.9503 | 362.6918 | 325.424 | 0.02204 |
| b1388 | *paaA* | 54.03439 | 40.07744 | 23.29207 | 50.8422 | 143.4699 | 192.9959 | 0.022223 |
| b0679 | *nagE* | 428.6118 | 451.1944 | 403.2439 | 516.0483 | 1427.812 | 1062.504 | 0.022296 |
| b2952 | *yggT* | 108.0688 | 96.96156 | 117.9161 | 166.9319 | 275.4621 | 189.9162 | 0.022355 |
| b1620 | *malI* | 177.6724 | 156.4313 | 113.5488 | 259.7189 | 259.3935 | 304.8925 | 0.022405 |
| b1745 | *astB* | 209.7267 | 258.5642 | 234.3764 | 158.0345 | 1562.1 | 1086.115 | 0.022717 |
| b0462 | *acrB* | 828.8327 | 972.2012 | 1055.422 | 805.0015 | 5353.148 | 3566.318 | 0.022763 |
| b1419 | *ydcA* | 93.41539 | 125.4036 | 115.0046 | 187.2688 | 313.3382 | 184.7833 | 0.023164 |
| b2467 | *nudK* | 116.3113 | 139.6246 | 133.9294 | 329.6269 | 210.0399 | 210.4477 | 0.023164 |
| b2733 | *mutS* | 212.4742 | 182.2877 | 203.8056 | 271.5821 | 417.7843 | 420.8953 | 0.023395 |
| b0578 | *nfsB* | 184.9991 | 204.2657 | 240.1995 | 239.8057 | 856.2282 | 583.094 | 0.023415 |
| b1445 | *ortT* | 22.89593 | 37.4918 | 40.76112 | 47.45272 | 161.834 | 95.47138 | 0.023415 |
| b1356 | *racR* | 273.8353 | 252.1 | 343.558 | 332.5927 | 1671.137 | 719.6284 | 0.023523 |
| b3256 | *accC* | 321.4589 | 404.6529 | 442.5493 | 514.7773 | 1303.854 | 852.0564 | 0.023526 |
| b1039 | *csgE* | 30.22263 | 20.68513 | 18.92481 | 47.87641 | 79.19537 | 48.24898 | 0.023569 |
| b3389 | *aroB* | 358.0923 | 449.9016 | 461.4741 | 552.0616 | 1228.102 | 926.9963 | 0.023584 |
| b1946 | *fliN* | 19.23258 | 28.44206 | 16.0133 | 48.72378 | 40.17156 | 75.96647 | 0.023741 |
| b2587 | *kgtP* | 5661.705 | 7657.377 | 7569.922 | 8041.965 | 33161.05 | 17432.25 | 0.023741 |
| b2472 | *dapE* | 229.8751 | 316.7411 | 286.7836 | 358.0138 | 735.7135 | 681.6451 | 0.023903 |
| b2796 | *sdaC* | 131.8806 | 116.3539 | 109.1816 | 199.132 | 191.6757 | 338.7694 | 0.024111 |
| b1706 | *ydiU* | 223.4643 | 265.0283 | 257.6685 | 224.5531 | 1365.833 | 845.897 | 0.02431 |
| b3073 | *patA* | 320.543 | 363.2826 | 467.2971 | 173.2872 | 4201.945 | 1647.651 | 0.024361 |
| b0405 | *queA* | 202.4 | 184.8734 | 228.5534 | 358.0138 | 389.0903 | 296.6799 | 0.024904 |
| b2707 | *srlR* | 76.93032 | 47.83437 | 66.9647 | 111.0055 | 160.6863 | 120.1092 | 0.024974 |
| b1035 | *ycdY* | 111.7321 | 151.26 | 147.0312 | 152.9503 | 562.4019 | 402.417 | 0.025133 |
| b3133 | *agaV* | 23.81177 | 32.32052 | 20.38056 | 41.52113 | 101.0028 | 62.62101 | 0.025475 |
| b4429 | *sokB* | 30.22263 | 25.85642 | 16.0133 | 40.25008 | 80.34313 | 68.78046 | 0.02556 |
| b4637 | *uof* | 253.6869 | 249.5144 | 273.6818 | 233.0268 | 1527.667 | 818.1795 | 0.025566 |
| b1535 | *dgcZ* | 236.286 | 214.6082 | 230.0092 | 315.6453 | 531.4124 | 407.5499 | 0.025725 |
| b2278 | *nuoL* | 269.2561 | 249.5144 | 193.6153 | 282.1742 | 887.2177 | 615.9444 | 0.025945 |
| b3033 | *yqiB* | 117.2272 | 118.9395 | 145.5754 | 188.9635 | 289.2353 | 226.8728 | 0.026008 |
| b1799 | *dmlR* | 155.6923 | 169.3595 | 183.425 | 210.5714 | 526.8214 | 403.4436 | 0.02607 |
| b2308 | *hisQ* | 79.67784 | 59.46975 | 34.9381 | 84.737 | 247.9159 | 158.0924 | 0.026462 |
| b1811 | *yoaH* | 87.92037 | 106.0113 | 115.0046 | 124.1397 | 525.6736 | 248.4309 | 0.026554 |
| b0025 | *ribF* | 448.7602 | 531.3493 | 687.116 | 694.8434 | 1744.594 | 1366.37 | 0.026616 |
| b4397 | *creA* | 88.83621 | 86.61899 | 77.15498 | 126.2581 | 183.6414 | 155.0127 | 0.026946 |
| b2693 | *argY* | 3.663349 | 1.292821 | 4.367263 | 9.32107 | 35.58053 | 11.29231 | 0.026946 |
| b2794 | *queF* | 146.5339 | 156.4313 | 157.2215 | 192.353 | 471.7289 | 352.1149 | 0.026946 |
| b1910 | *cysT* | 31.13846 | 31.0277 | 18.92481 | 27.53953 | 202.0056 | 91.36508 | 0.026986 |
| b0470 | *dnaX* | 629.1801 | 682.6094 | 740.9789 | 917.7017 | 1265.978 | 1253.447 | 0.027012 |
| b4668 | *ibsB* | 73.26697 | 62.0554 | 61.14168 | 102.9555 | 122.8102 | 140.6406 | 0.027104 |
| b0854 | *potF* | 112.648 | 102.1328 | 91.71252 | 127.5292 | 386.7948 | 246.3778 | 0.027144 |
| b4172 | *hfq* | 925.9114 | 1274.721 | 1282.52 | 1153.271 | 4692.039 | 3833.227 | 0.027153 |
| b3313 | *rplP* | 520.1955 | 831.2837 | 1026.307 | 579.6011 | 4602.513 | 3686.427 | 0.027153 |
| b3847 | *pepQ* | 435.0227 | 562.377 | 625.9743 | 633.4091 | 1857.074 | 1407.433 | 0.027478 |
| b2762 | *cysH* | 70.51946 | 58.17693 | 62.59743 | 77.95804 | 173.3116 | 230.9791 | 0.02829 |
| b1014 | *putA* | 446.0127 | 422.7524 | 314.4429 | 394.4507 | 1709.013 | 1227.782 | 0.028946 |
| b1823 | *cspC* | 457.9186 | 819.6484 | 943.3288 | 218.6215 | 6784.403 | 5208.836 | 0.029687 |
| b0686 | *ybfF* | 89.75204 | 107.3041 | 117.9161 | 154.2213 | 294.974 | 194.0225 | 0.029697 |
| b0921 | *cmoM* | 227.1276 | 244.3431 | 288.2393 | 341.9138 | 632.4152 | 484.5429 | 0.030079 |
| b2551 | *glyA* | 597.1258 | 608.9186 | 962.2536 | 602.4801 | 3603.963 | 2846.69 | 0.030121 |
| b1054 | *lpxL* | 281.162 | 288.299 | 278.0491 | 369.4533 | 1080.041 | 552.2968 | 0.030298 |
| b1550 | *gnsB* | 27.47512 | 51.71283 | 68.42045 | 46.18167 | 278.9054 | 209.4211 | 0.030696 |
| b1655 | *mepH* | 99.82625 | 102.1328 | 123.7391 | 137.6976 | 330.5546 | 252.5372 | 0.030741 |
| b0800 | *ybiB* | 259.1819 | 246.9288 | 283.8721 | 316.4927 | 686.3598 | 640.5822 | 0.031032 |
| b2677 | *proV* | 510.1213 | 677.4381 | 877.8198 | 1198.181 | 1855.926 | 1091.248 | 0.031032 |
| b3616 | *tdh* | 255.5186 | 292.1775 | 291.1509 | 295.7321 | 990.516 | 801.7543 | 0.031032 |
| b4144 | *yjeI* | 213.3901 | 307.6913 | 296.9739 | 269.8873 | 977.8906 | 985.511 | 0.031093 |
| b1746 | *astD* | 337.9439 | 297.3488 | 324.6332 | 186.8451 | 2096.956 | 1434.124 | 0.031327 |
| b3150 | *yraP* | 108.9846 | 121.5252 | 132.4736 | 136.8503 | 424.6708 | 326.4505 | 0.031784 |
| b1954 | *dsrA* | 10.99005 | 12.92821 | 8.734526 | 15.25266 | 60.83122 | 40.03639 | 0.032143 |
| b4536 | *yobH* | 43.04435 | 41.37026 | 72.78771 | 52.96063 | 439.5917 | 130.3749 | 0.032371 |
| b2874 | *yqeA* | 131.8806 | 165.4811 | 158.6772 | 227.5188 | 249.0637 | 341.8491 | 0.032806 |
| b1296 | *puuP* | 148.3656 | 99.5472 | 101.9028 | 167.7793 | 697.8374 | 208.3945 | 0.032986 |
| b3535 | *yhjR* | 30.22263 | 42.66308 | 27.65933 | 36.8606 | 191.6757 | 100.6042 | 0.033025 |
| b0742 | *cpoB* | 236.286 | 341.3047 | 379.9519 | 336.4059 | 1344.026 | 912.6243 | 0.033132 |
| b1959 | *yedA* | 106.2371 | 96.96156 | 104.8143 | 166.5082 | 165.2773 | 170.4113 | 0.033378 |
| b1750 | *ydjX* | 56.78191 | 29.73488 | 27.65933 | 182.6082 | 56.24019 | 94.44481 | 0.033378 |
| b4640 | *-* | 24.7276 | 37.4918 | 16.0133 | 62.70538 | 49.35363 | 71.86018 | 0.03368 |
| b1855 | *lpxM* | 226.2118 | 230.1221 | 234.3764 | 377.5033 | 413.1932 | 317.2114 | 0.03368 |
| b2698 | *recX* | 42.12851 | 59.46975 | 34.9381 | 72.45014 | 117.0714 | 100.6042 | 0.03368 |
| b0785 | *moaE* | 43.96018 | 43.95591 | 29.11509 | 60.16327 | 90.67296 | 105.7371 | 0.033686 |
| b3957 | *argE* | 140.1231 | 149.9672 | 174.6905 | 200.8267 | 418.932 | 319.2645 | 0.03432 |
| b1033 | *ghrA* | 204.2317 | 240.4647 | 246.0225 | 297.0032 | 472.8767 | 503.0212 | 0.034923 |
| b4322 | *uxuA* | 660.3186 | 637.3606 | 822.5012 | 991.8466 | 1408.3 | 1160.029 | 0.035633 |
| b3520 | *yhjB* | 113.5638 | 108.5969 | 94.62403 | 167.7793 | 180.1982 | 173.491 | 0.035842 |
| b0766 | *ybhA* | 66.85611 | 60.76258 | 68.42045 | 104.6502 | 110.1849 | 128.3217 | 0.035842 |
| b3149 | *diaA* | 79.67784 | 98.25438 | 119.3719 | 117.7844 | 333.9978 | 257.6701 | 0.035925 |
| b4054 | *tyrB* | 228.0435 | 228.8293 | 193.6153 | 269.4637 | 491.2408 | 499.9415 | 0.03613 |
| b0452 | *tesB* | 156.6082 | 165.4811 | 213.9959 | 123.716 | 1057.086 | 687.8046 | 0.03613 |
| b0895 | *dmsB* | 54.95023 | 55.59129 | 50.9514 | 135.1555 | 73.45657 | 114.9763 | 0.036165 |
| b4656 | *-* | 115.3955 | 109.8898 | 148.4869 | 124.1397 | 521.0826 | 374.6995 | 0.036188 |
| b2822 | *recC* | 416.7059 | 461.537 | 429.4475 | 559.6879 | 959.5265 | 799.7011 | 0.036308 |
| b0490 | *fetA* | 67.77195 | 58.17693 | 80.06649 | 113.9713 | 123.958 | 123.1889 | 0.036333 |
| b3983 | *rplK* | 606.2842 | 844.212 | 1112.196 | 835.0831 | 3279.147 | 2917.523 | 0.036544 |
| b1939 | *fliG* | 68.68779 | 65.93386 | 49.49565 | 91.09228 | 119.3669 | 178.6239 | 0.036891 |
| b3785 | *wzzE* | 247.276 | 199.0944 | 250.3897 | 344.4559 | 490.0931 | 364.4338 | 0.036891 |
| b0988 | *insB-4* | 2.747512 | 3.878462 | 2.911509 | 14.40529 | 17.21638 | 9.239166 | 0.036891 |
| b0180 | *fabZ* | 508.2896 | 668.3883 | 713.3196 | 765.5988 | 1781.322 | 1394.087 | 0.03745 |
| b2516 | *rodZ* | 463.4136 | 628.3109 | 710.4081 | 726.6198 | 2072.853 | 1331.466 | 0.037558 |
| b0675 | *umpH* | 163.019 | 208.1441 | 232.9207 | 258.0242 | 542.89 | 428.0813 | 0.037604 |
| b1084 | *rne* | 1245.539 | 1406.589 | 2026.41 | 1822.693 | 4659.901 | 4051.887 | 0.037709 |
| b1174 | *minE* | 42.12851 | 50.42001 | 65.50894 | 37.28428 | 306.4516 | 224.8197 | 0.038068 |
| b1064 | *grxB* | 323.2905 | 482.2221 | 678.3815 | 599.938 | 1792.799 | 1298.616 | 0.038396 |
| b3892 | *fdoI* | 493.6362 | 517.1283 | 551.7309 | 605.4459 | 2196.811 | 1069.69 | 0.038586 |
| b3291 | *mscL* | 164.8507 | 180.9949 | 168.8675 | 153.7977 | 735.7135 | 538.9513 | 0.039235 |
| b2315 | *folC* | 166.6824 | 212.0226 | 180.5135 | 235.1452 | 420.0798 | 409.603 | 0.039502 |
| b1539 | *ydfG* | 241.781 | 303.8129 | 343.558 | 319.8822 | 967.5608 | 799.7011 | 0.039559 |
| b1943 | *fliK* | 31.13846 | 27.14924 | 24.74782 | 50.8422 | 48.20588 | 88.28536 | 0.039638 |
| b2800 | *fucA* | 65.94028 | 34.90616 | 42.21687 | 77.53436 | 112.4804 | 117.0294 | 0.040061 |
| b3780 | *rhlB* | 618.1901 | 734.3222 | 949.1518 | 982.9492 | 1928.235 | 1604.535 | 0.040315 |
| b2434 | *ypeA* | 100.7421 | 122.818 | 84.43375 | 119.9029 | 340.8844 | 259.7232 | 0.040315 |
| b3308 | *rplE* | 1167.692 | 2287 | 2540.291 | 2096.817 | 8810.198 | 5894.588 | 0.040406 |
| b1335 | *ogt* | 98.91042 | 77.56925 | 103.3586 | 138.545 | 261.689 | 161.1721 | 0.040533 |
| b1623 | *add* | 181.3358 | 202.9729 | 206.7171 | 266.4979 | 425.8186 | 336.7163 | 0.040638 |
| b0773 | *ybhB* | 58.61358 | 59.46975 | 90.25677 | 94.90544 | 189.3802 | 159.119 | 0.040767 |
| b3105 | *yhaJ* | 177.6724 | 143.5031 | 138.2967 | 227.9425 | 339.7366 | 246.3778 | 0.041293 |
| b3347 | *fkpA* | 379.1566 | 564.9627 | 554.6424 | 440.2087 | 1995.953 | 1706.166 | 0.041293 |
| b1658 | *purR* | 314.1322 | 354.2329 | 416.3457 | 368.1823 | 1365.833 | 948.5544 | 0.041527 |
| b0080 | *cra* | 319.6272 | 363.2826 | 409.067 | 380.4691 | 1220.068 | 953.6872 | 0.041975 |
| b3439 | *yhhW* | 272.0036 | 371.0396 | 349.381 | 498.2536 | 550.9243 | 532.7919 | 0.042086 |
| b0853 | *ybjN* | 185.9149 | 214.6082 | 235.8322 | 280.9032 | 532.5601 | 383.9387 | 0.042299 |
| b2700 | *pncC* | 127.3014 | 117.6467 | 119.3719 | 148.2898 | 363.8396 | 263.8295 | 0.042537 |
| b0926 | *ycbK* | 511.953 | 608.9186 | 685.6603 | 652.8986 | 1915.61 | 1483.399 | 0.042679 |
| b2960 | *trmI* | 134.6281 | 171.9452 | 199.4383 | 195.7425 | 606.0167 | 388.045 | 0.042744 |
| b0447 | *decR* | 115.3955 | 135.7462 | 113.5488 | 133.0371 | 378.7604 | 322.3442 | 0.042751 |
| b2569 | *lepA* | 567.8191 | 712.3442 | 745.3462 | 753.7356 | 1850.187 | 1670.236 | 0.04284 |
| b2202 | *napC* | 87.00453 | 113.7682 | 107.7258 | 200.8267 | 188.2325 | 145.7735 | 0.04284 |
| b0175 | *cdsA* | 258.2661 | 321.9124 | 257.6685 | 306.7479 | 952.6399 | 662.1402 | 0.042967 |
| b1376 | *uspF* | 379.1566 | 489.9791 | 502.2352 | 660.1012 | 1223.511 | 726.8144 | 0.043057 |
| b2836 | *aas* | 216.1376 | 217.1939 | 211.0844 | 255.0584 | 513.0482 | 474.2772 | 0.043286 |
| b2951 | *yggS* | 74.18281 | 71.10514 | 94.62403 | 144.0529 | 187.0847 | 114.9763 | 0.043307 |
| b3599 | *mtlA* | 250.9394 | 236.5862 | 227.0977 | 220.7399 | 1089.223 | 631.343 | 0.043734 |
| b2689 | *yqaA* | 107.153 | 118.9395 | 104.8143 | 135.5792 | 290.383 | 239.1917 | 0.043734 |
| b0057 | *yabQ* | 13.73756 | 14.22103 | 11.64603 | 19.9132 | 57.38795 | 36.95666 | 0.043734 |
| b1424 | *opgD* | 206.9792 | 245.6359 | 231.4649 | 259.2952 | 754.0776 | 499.9415 | 0.044502 |
| b2041 | *rfbB* | 283.9095 | 387.8462 | 379.9519 | 430.0403 | 836.7163 | 761.7179 | 0.044589 |
| b0796 | *cecR* | 154.7765 | 204.2657 | 232.9207 | 381.3165 | 383.3515 | 275.1218 | 0.044758 |
| b3866 | *yihI* | 97.99458 | 122.818 | 145.5754 | 138.545 | 453.3648 | 286.4141 | 0.04603 |
| b4359 | *opgB* | 250.9394 | 213.3154 | 200.8941 | 277.9374 | 476.32 | 455.7988 | 0.046654 |
| b0653 | *gltK* | 95.24707 | 67.22668 | 66.9647 | 118.6318 | 223.813 | 130.3749 | 0.046701 |
| b3862 | *yihG* | 51.28688 | 58.17693 | 43.67263 | 111.4292 | 89.5252 | 80.07277 | 0.046701 |
| b3307 | *rpsN* | 1476.33 | 2405.939 | 3017.779 | 2451.018 | 8275.342 | 6560.834 | 0.046789 |
| b3833 | *ubiE* | 369.9982 | 456.3657 | 537.1733 | 625.3591 | 949.1966 | 784.3025 | 0.046793 |
| b2188 | *yejM* | 194.1575 | 192.6303 | 203.8056 | 272.4295 | 464.8424 | 312.0785 | 0.046897 |
| b1329 | *mppA* | 244.5285 | 221.0723 | 282.4163 | 244.4662 | 1021.505 | 631.343 | 0.047176 |
| b1659 | *ydhB* | 141.9548 | 168.0667 | 88.80101 | 236.4162 | 322.5203 | 214.554 | 0.0472 |
| b0516 | *allC* | 114.4796 | 113.7682 | 115.0046 | 168.6266 | 187.0847 | 185.8099 | 0.0472 |
| b2581 | *yfiF* | 465.2453 | 484.8078 | 470.2086 | 486.3904 | 1463.393 | 1190.826 | 0.047509 |
| b2562 | *yfhL* | 39.381 | 34.90616 | 32.02659 | 58.46853 | 73.45657 | 68.78046 | 0.048056 |
| b1015 | *putP* | 317.7955 | 333.5478 | 296.9739 | 379.1981 | 810.3178 | 621.0773 | 0.048056 |
| b3590 | *selB* | 343.4389 | 360.697 | 384.3191 | 687.6408 | 446.4782 | 727.8409 | 0.048105 |
| b3225 | *nanA* | 80.59367 | 55.59129 | 40.76112 | 122.445 | 120.5147 | 103.684 | 0.048787 |
| b2611 | *ypjD* | 109.9005 | 100.84 | 71.33196 | 161.8477 | 239.8816 | 141.6672 | 0.049272 |
| b4393 | *trpR* | 81.50951 | 86.61899 | 100.447 | 118.6318 | 454.5125 | 149.8798 | 0.049745 |

Table S3. Gene Ontology analysis of DEGs.

| Category | GO ID | Description | padj | Up | Up_Gene_id | Down | Down_Gene_id |
| --- | --- | --- | --- | --- | --- | --- | --- |
| BP | GO:0044267 | cellular protein metabolic process | 3.05E-05 | 8 | b3932/b1129/b1716/b2214/b1717/b2218/b2991/b2184 | 46 | b0023/b3703/b4200/b1089/b2609/b4202/b3231/b2171/b0525/b3310/b0781/b3309/b3185/b4147/b3165/b3636/b3316/b3230/b3936/b3318/b3319/b3363/b3320/b3317/b2606/b0170/b3315/b0680/b1637/b3311/b1876/b0526/b3342/b3314/b2697/b3065/b3312/b3306/b0783/b2828/b1866/b3313/b0785/b3983/b3307/b3590 |
| BP | GO:0006518 | peptide metabolic process | 0.000191 | 4 | b1716/b1717/b2688/b2184 | 41 | b0023/b3703/b4200/b1089/b2609/b4202/b3231/b2171/b3310/b3309/b3185/b4147/b3165/b3636/b3316/b3230/b1218/b3936/b3318/b3319/b3320/b3317/b2606/b0170/b3315/b0680/b1637/b3311/b1876/b0526/b3342/b3314/b2697/b3065/b3312/b3306/b1866/b3313/b3983/b3307/b3590 |
| BP | GO:0043043 | peptide biosynthetic process | 0.000191 | 4 | b1716/b1717/b2688/b2184 | 40 | b0023/b3703/b4200/b1089/b2609/b4202/b3231/b2171/b3310/b3309/b3185/b4147/b3165/b3636/b3316/b3230/b3936/b3318/b3319/b3320/b3317/b2606/b0170/b3315/b0680/b1637/b3311/b1876/b0526/b3342/b3314/b2697/b3065/b3312/b3306/b1866/b3313/b3983/b3307/b3590 |
| BP | GO:0006412 | translation | 0.000191 | 3 | b1716/b1717/b2184 | 40 | b0023/b3703/b4200/b1089/b2609/b4202/b3231/b2171/b3310/b3309/b3185/b4147/b3165/b3636/b3316/b3230/b3936/b3318/b3319/b3320/b3317/b2606/b0170/b3315/b0680/b1637/b3311/b1876/b0526/b3342/b3314/b2697/b3065/b3312/b3306/b1866/b3313/b3983/b3307/b3590 |
| BP | GO:0043604 | amide biosynthetic process | 0.000952 | 4 | b1716/b1717/b2688/b2184 | 40 | b0023/b3703/b4200/b1089/b2609/b4202/b3231/b2171/b3310/b3309/b3185/b4147/b3165/b3636/b3316/b3230/b3936/b3318/b3319/b3320/b3317/b2606/b0170/b3315/b0680/b1637/b3311/b1876/b0526/b3342/b3314/b2697/b3065/b3312/b3306/b1866/b3313/b3983/b3307/b3590 |
| BP | GO:0043603 | cellular amide metabolic process | 0.001888 | 4 | b1716/b1717/b2688/b2184 | 41 | b0023/b3703/b4200/b1089/b2609/b4202/b3231/b2171/b3310/b3309/b3185/b4147/b3165/b3636/b3316/b3230/b1218/b3936/b3318/b3319/b3320/b3317/b2606/b0170/b3315/b0680/b1637/b3311/b1876/b0526/b3342/b3314/b2697/b3065/b3312/b3306/b1866/b3313/b3983/b3307/b3590 |
| BP | GO:1901576 | organic substance biosynthetic process | 0.002 | 36 | b4293/b4116/b4372/b3507/b3512/b2289/b2942/b0981/b4063/b0020/b2015/b3994/b4241/b3774/b2213/b3212/b0523/b3289/b3515/b1716/b1717/b3987/b2688/b0414/b2218/b0184/b3530/b0400/b0487/b2184/b3988/b0096/b0475/b2577/b0546/b4481 | 145 | b1892/b0023/b1552/b3703/b4200/b1089/b2609/b4202/b1558/b1891/b4018/b0931/b3231/b3738/b1090/b2350/b2171/b3736/b3403/b1608/b3310/b2667/b0610/b1922/b4062/b1305/b4052/b3357/b1275/b3418/b0131/b0781/b3309/b3185/b1557/b3041/b3735/b2027/b3059/b0730/b4147/b0240/b3165/b2599/b2234/b3075/b3636/b0685/b1297/b3627/b1091/b0421/b3316/b0109/b1339/b3230/b3733/b3734/b3936/b1384/b3318/b0750/b3319/b2537/b3995/b4118/b3961/b2869/b2916/b3320/b3317/b2606/b3755/b2573/b0170/b0048/b1842/b2980/b3243/b3066/b0990/b3649/b3863/b1814/b3702/b3604/b4039/b3315/b3954/b0316/b0680/b1637/b3311/b3382/b1071/b1876/b1916/b1610/b3626/b0526/b3342/b3314/b2697/b1422/b3925/b1770/b2157/b3065/b3312/b1284/b3306/b1187/b3556/b0420/b0783/b0346/b2828/b2805/b3997/b1866/b2217/b0345/b1620/b0405/b2707/b1799/b0025/b2794/b0470/b4172/b3313/b1014/b1823/b0785/b3520/b3983/b3785/b3105/b1658/b0080/b1424/b3307/b1659/b3590/b4393 |
| BP | GO:1901564 | organonitrogen compound metabolic process | 0.002027 | 16 | b2143/b0485/b3994/b3774/b3212/b0523/b1716/b1717/b2440/b2688/b0414/b2441/b2388/b2784/b2184/b0475 | 78 | b2870/b0023/b3703/b4200/b3708/b1089/b2609/b4202/b0931/b3231/b3738/b2171/b3736/b3310/b0131/b0781/b3309/b3185/b0125/b2817/b3041/b3735/b1854/b4147/b3165/b2599/b3636/b1297/b3316/b0109/b3230/b3733/b3734/b1218/b0238/b3936/b3318/b0469/b0750/b3319/b1747/b3320/b3317/b4382/b2606/b0170/b0048/b3831/b3732/b3315/b0680/b3177/b1637/b3311/b1876/b0002/b1262/b1260/b0526/b3342/b3314/b2697/b3065/b3312/b3306/b2903/b0783/b3997/b1866/b1745/b0405/b0025/b2794/b3313/b0785/b3983/b3307/b3590 |
| BP | GO:0009059 | macromolecule biosynthetic process | 0.002027 | 27 | b4293/b4116/b4372/b3507/b3512/b2289/b0981/b4063/b0020/b2015/b4241/b2213/b3289/b3515/b1716/b1717/b3987/b2218/b0184/b3530/b0400/b0487/b2184/b3988/b2577/b0546/b4481 | 115 | b1892/b0023/b1552/b3703/b4200/b1089/b2609/b4202/b1558/b1891/b4018/b3231/b2350/b2171/b1608/b3310/b2667/b0610/b1922/b4062/b1305/b4052/b3357/b1275/b3418/b3309/b3185/b1557/b2027/b0730/b4147/b0240/b3165/b2234/b3075/b3636/b0685/b3627/b3316/b1339/b3230/b3936/b1384/b3318/b3319/b2537/b3995/b4118/b3961/b2869/b2916/b3320/b3317/b2606/b3755/b2573/b0170/b1842/b2980/b3243/b3066/b0990/b3649/b3863/b3702/b3604/b3315/b3954/b0316/b0680/b1637/b3311/b3382/b1071/b1876/b1916/b1610/b3626/b0526/b3342/b3314/b2697/b1422/b1770/b2157/b3065/b3312/b1284/b3306/b1187/b3556/b0346/b2828/b2805/b1866/b2217/b0345/b1620/b2707/b1799/b0470/b4172/b3313/b1014/b1823/b3520/b3983/b3785/b3105/b1658/b0080/b3307/b1659/b3590/b4393 |
| BP | GO:0034645 | cellular macromolecule biosynthetic process | 0.002044 | 27 | b4293/b4116/b4372/b3507/b3512/b2289/b0981/b4063/b0020/b2015/b4241/b2213/b3289/b3515/b1716/b1717/b3987/b2218/b0184/b3530/b0400/b0487/b2184/b3988/b2577/b0546/b4481 | 114 | b1892/b0023/b1552/b3703/b4200/b1089/b2609/b4202/b1558/b1891/b4018/b3231/b2171/b1608/b3310/b2667/b0610/b1922/b4062/b1305/b4052/b3357/b1275/b3418/b3309/b3185/b1557/b2027/b0730/b4147/b0240/b3165/b2234/b3075/b3636/b0685/b3627/b3316/b1339/b3230/b3936/b1384/b3318/b3319/b2537/b3995/b4118/b3961/b2869/b2916/b3320/b3317/b2606/b3755/b2573/b0170/b1842/b2980/b3243/b3066/b0990/b3649/b3863/b3702/b3604/b3315/b3954/b0316/b0680/b1637/b3311/b3382/b1071/b1876/b1916/b1610/b3626/b0526/b3342/b3314/b2697/b1422/b1770/b2157/b3065/b3312/b1284/b3306/b1187/b3556/b0346/b2828/b2805/b1866/b2217/b0345/b1620/b2707/b1799/b0470/b4172/b3313/b1014/b1823/b3520/b3983/b3785/b3105/b1658/b0080/b3307/b1659/b3590/b4393 |
| BP | GO:0044249 | cellular biosynthetic process | 0.002044 | 39 | b4293/b4116/b4372/b3507/b3512/b2289/b2942/b0981/b4063/b0020/b3808/b2015/b3994/b4241/b3774/b2213/b3212/b0523/b3289/b1683/b3515/b1716/b1717/b3987/b2688/b0414/b2218/b1681/b0184/b3530/b0400/b0487/b2184/b3988/b0096/b0475/b2577/b0546/b4481 | 140 | b1892/b0023/b1552/b3703/b4200/b1089/b2609/b4202/b1558/b1891/b4018/b0931/b3231/b3738/b1090/b2171/b3736/b1608/b3310/b2667/b0610/b1922/b4062/b1305/b4052/b3357/b1275/b3418/b0131/b0781/b3309/b3185/b1557/b3041/b3735/b2027/b3059/b0730/b4147/b0240/b3165/b2599/b2234/b3075/b3636/b0685/b1297/b3627/b1091/b0421/b3316/b0109/b1339/b3230/b3733/b3734/b3936/b1384/b3318/b0750/b3319/b2537/b3995/b4118/b3961/b2869/b2916/b3320/b3317/b2606/b3755/b2573/b0170/b0048/b1842/b2980/b3243/b3066/b0990/b3649/b3863/b3702/b3604/b4039/b3315/b3954/b0316/b0680/b1637/b3311/b3382/b1071/b1876/b1916/b1610/b3626/b0526/b3342/b3314/b2697/b1422/b1770/b2157/b3065/b3312/b1284/b3306/b1187/b3556/b0420/b0783/b0346/b2828/b2805/b3997/b1866/b2217/b0345/b1620/b0405/b2707/b1799/b0025/b2794/b0470/b4172/b3313/b1014/b1823/b0785/b3520/b3983/b3785/b3105/b1658/b0080/b3307/b1659/b3590/b4393 |
| BP | GO:0019538 | protein metabolic process | 0.002183 | 11 | b2592/b3932/b1129/b2134/b1716/b2214/b1717/b2218/b2991/b2184/b0439 | 51 | b0023/b3703/b4200/b1089/b2609/b4202/b3231/b4021/b2171/b0525/b3310/b0781/b3309/b3185/b4043/b4147/b0632/b3165/b3636/b3316/b3230/b3936/b3318/b1488/b3319/b3363/b3320/b3317/b2606/b0170/b3315/b1538/b0680/b1637/b3311/b1876/b0526/b3342/b3314/b2697/b3065/b3312/b3306/b0783/b2828/b1866/b3313/b0785/b3983/b3307/b3590 |
| BP | GO:1901566 | organonitrogen compound biosynthetic process | 0.00282 | 10 | b3994/b3774/b3212/b0523/b1716/b1717/b2688/b0414/b2184/b0475 | 60 | b0023/b3703/b4200/b1089/b2609/b4202/b0931/b3231/b3738/b2171/b3736/b3310/b0131/b0781/b3309/b3185/b3041/b3735/b4147/b3165/b2599/b3636/b1297/b3316/b0109/b3230/b3733/b3734/b3936/b3318/b0750/b3319/b3320/b3317/b2606/b0170/b0048/b3315/b0680/b1637/b3311/b1876/b0526/b3342/b3314/b2697/b3065/b3312/b3306/b0783/b3997/b1866/b0405/b0025/b2794/b3313/b0785/b3983/b3307/b3590 |
| BP | GO:0010467 | gene expression | 0.002831 | 30 | b4293/b4116/b3507/b3512/b2289/b3651/b4063/b0020/b2015/b4241/b2213/b3289/b3167/b3515/b1716/b1717/b0188/b3497/b3343/b3987/b2218/b4371/b3166/b0400/b0487/b4180/b2184/b3988/b2577/b0546 | 112 | b1892/b3260/b0023/b1552/b3703/b4200/b1089/b2609/b4202/b1558/b1891/b4018/b3231/b3704/b2608/b2171/b1608/b3310/b2667/b0610/b1922/b4062/b1305/b0406/b3357/b1275/b3418/b3309/b3185/b1557/b0730/b4147/b0240/b3165/b4049/b3075/b3636/b0685/b3316/b1339/b3230/b3936/b1384/b3318/b3319/b2537/b3995/b4118/b3961/b2869/b2916/b3320/b3317/b3164/b2606/b3755/b2573/b0170/b3741/b2980/b3243/b0990/b3649/b2696/b3604/b3315/b3954/b0316/b0680/b0143/b1637/b3311/b3382/b1071/b1876/b1916/b0526/b3342/b3314/b2697/b1422/b1770/b2157/b3065/b3312/b1284/b3306/b1187/b3556/b0346/b2805/b1866/b2217/b0345/b1620/b2707/b1799/b4172/b3313/b1014/b1823/b3520/b3983/b3105/b1658/b0080/b2960/b3307/b1659/b2581/b3590/b4393 |
| BP | GO:0044271 | cellular nitrogen compound biosynthetic process | 0.012307 | 27 | b4293/b4116/b3507/b3512/b2289/b4063/b0020/b2015/b3994/b4241/b2213/b0523/b3289/b3515/b1716/b1717/b3987/b2688/b0414/b2218/b0400/b0487/b2184/b3988/b0475/b2577/b0546 | 116 | b1892/b0023/b1552/b3703/b4200/b1089/b2609/b4202/b1558/b1891/b4018/b0931/b3231/b3738/b2171/b3736/b1608/b3310/b2667/b0610/b1922/b4062/b1305/b3357/b1275/b3418/b3309/b3185/b1557/b3041/b3735/b0730/b4147/b0240/b3165/b3075/b3636/b0685/b3316/b0109/b1339/b3230/b3733/b3734/b3936/b1384/b3318/b0750/b3319/b2537/b3995/b4118/b3961/b2869/b2916/b3320/b3317/b2606/b3755/b2573/b0170/b0048/b2980/b3243/b3066/b0990/b3649/b3604/b3315/b3954/b0316/b0680/b1637/b3311/b3382/b1071/b1876/b1916/b0526/b3342/b3314/b2697/b1422/b1770/b2157/b3065/b3312/b1284/b3306/b1187/b3556/b0346/b2805/b3997/b1866/b2217/b0345/b1620/b0405/b2707/b1799/b0025/b2794/b4172/b3313/b1014/b1823/b3520/b3983/b3105/b1658/b0080/b3307/b1659/b3590/b4393 |
| BP | GO:0006629 | lipid metabolic process | 0.021687 | 5 | b0981/b4377/b0090/b3449/b0096 | 17 | b2533/b2341/b3846/b0221/b1090/b2027/b3059/b3627/b1091/b0421/b0418/b3626/b3821/b2239/b1187/b0420/b3785 |
| BP | GO:0015980 | energy derivation by oxidation of organic compounds | 0.036964 | 3 | b0978/b0974/b0733 | 8 | b4139/b1611/b0431/b1418/b1057/b0118/b0896/b3892 |
| BP | GO:0045333 | cellular respiration | 0.036964 | 3 | b0978/b0974/b0733 | 8 | b4139/b1611/b0431/b1418/b1057/b0118/b0896/b3892 |
| BP | GO:0006457 | protein folding | 0.048634 | 3 | b1000/b0473/b0015 | 5 | b0436/b4207/b0525/b3363/b3347 |
| BP | GO:1901137 | carbohydrate derivative biosynthetic process | 0.048634 | 4 | b0981/b0523/b0096/b4481 | 11 | b3738/b3736/b3735/b2027/b3627/b3733/b3734/b3626/b0405/b2794/b3785 |
| CC | GO:0043229 | intracellular organelle | 2.39E-08 | 2 | b1716/b1717 | 33 | b0023/b3703/b4200/b1089/b2609/b3186/b4202/b1274/b3231/b3310/b3309/b3185/b3165/b3636/b3316/b3230/b3936/b3318/b3319/b3320/b3317/b2606/b2231/b3315/b3311/b3342/b3314/b3065/b3312/b3306/b3313/b3983/b3307 |
| CC | GO:0043232 | intracellular non-membrane-bounded organelle | 2.39E-08 | 2 | b1716/b1717 | 33 | b0023/b3703/b4200/b1089/b2609/b3186/b4202/b1274/b3231/b3310/b3309/b3185/b3165/b3636/b3316/b3230/b3936/b3318/b3319/b3320/b3317/b2606/b2231/b3315/b3311/b3342/b3314/b3065/b3312/b3306/b3313/b3983/b3307 |
| CC | GO:0005840 | ribosome | 2.39E-08 | 2 | b1716/b1717 | 31 | b0023/b3703/b4200/b1089/b2609/b3186/b4202/b3231/b3310/b3309/b3185/b3165/b3636/b3316/b3230/b3936/b3318/b3319/b3320/b3317/b2606/b3315/b3311/b3342/b3314/b3065/b3312/b3306/b3313/b3983/b3307 |
| CC | GO:0030529 | intracellular ribonucleoprotein complex | 3.53E-08 | 2 | b1716/b1717 | 31 | b0023/b3703/b4200/b1089/b2609/b3186/b4202/b3231/b3310/b3309/b3185/b3165/b3636/b3316/b3230/b3936/b3318/b3319/b3320/b3317/b2606/b3315/b3311/b3342/b3314/b3065/b3312/b3306/b3313/b3983/b3307 |
| CC | GO:1990904 | ribonucleoprotein complex | 3.53E-08 | 2 | b1716/b1717 | 31 | b0023/b3703/b4200/b1089/b2609/b3186/b4202/b3231/b3310/b3309/b3185/b3165/b3636/b3316/b3230/b3936/b3318/b3319/b3320/b3317/b2606/b3315/b3311/b3342/b3314/b3065/b3312/b3306/b3313/b3983/b3307 |
| CC | GO:0032991 | macromolecular complex | 7.65E-08 | 5 | b0978/b3932/b0733/b1716/b1717 | 40 | b0023/b3703/b3194/b4200/b1089/b1126/b2609/b3186/b4202/b3231/b3737/b3738/b3736/b3310/b0781/b3309/b3185/b1859/b3165/b0422/b3636/b3316/b3230/b3733/b3936/b3318/b3319/b3320/b3317/b2606/b3315/b3311/b3342/b3314/b3065/b3312/b3306/b3313/b3983/b3307 |
| CC | GO:0044424 | intracellular part | 7.65E-08 | 6 | b0585/b3932/b1716/b1717/b0188/b3343 | 54 | b0023/b3703/b4200/b1089/b4226/b4016/b2609/b3186/b4202/b1274/b3231/b3738/b2171/b3736/b3310/b3309/b3185/b4147/b0240/b3165/b0422/b3636/b3316/b3230/b3733/b3936/b3318/b3319/b3320/b3317/b2606/b2231/b4039/b3315/b0680/b3311/b1876/b1610/b0526/b3342/b3314/b2697/b3065/b3312/b0382/b1817/b3306/b3891/b1866/b3133/b3313/b3983/b3307/b3590 |
| CC | GO:0044444 | cytoplasmic part | 1.16E-07 | 2 | b1716/b1717 | 31 | b0023/b3703/b4200/b1089/b2609/b3186/b4202/b3231/b3310/b3309/b3185/b3165/b3636/b3316/b3230/b3936/b3318/b3319/b3320/b3317/b2606/b3315/b3311/b3342/b3314/b3065/b3312/b3306/b3313/b3983/b3307 |
| CC | GO:0043226 | organelle | 2.20E-07 | 2 | b1716/b1717 | 35 | b0023/b3703/b4200/b1089/b2609/b3186/b4202/b1274/b3231/b1944/b3310/b3309/b3185/b3165/b3636/b3316/b3230/b3936/b3318/b3319/b3320/b3317/b2606/b2231/b3315/b3311/b3342/b3314/b3065/b3312/b1945/b3306/b3313/b3983/b3307 |
| CC | GO:0043228 | non-membrane-bounded organelle | 2.20E-07 | 2 | b1716/b1717 | 35 | b0023/b3703/b4200/b1089/b2609/b3186/b4202/b1274/b3231/b1944/b3310/b3309/b3185/b3165/b3636/b3316/b3230/b3936/b3318/b3319/b3320/b3317/b2606/b2231/b3315/b3311/b3342/b3314/b3065/b3312/b1945/b3306/b3313/b3983/b3307 |
| CC | GO:0005737 | cytoplasm | 4.50E-07 | 5 | b0585/b1716/b1717/b0188/b3343 | 48 | b0023/b3703/b4200/b1089/b4226/b4016/b2609/b3186/b4202/b3231/b2171/b3310/b3309/b3185/b4147/b0240/b3165/b3636/b3316/b3230/b3936/b3318/b3319/b3320/b3317/b2606/b4039/b3315/b0680/b3311/b1876/b1610/b0526/b3342/b3314/b2697/b3065/b3312/b0382/b1817/b3306/b3891/b1866/b3133/b3313/b3983/b3307/b3590 |
| CC | GO:0005622 | intracellular | 6.94E-07 | 6 | b0585/b3932/b1716/b1717/b0188/b3343 | 61 | b0023/b3703/b4200/b1089/b4226/b4016/b2609/b3186/b4202/b1274/b3231/b3738/b2171/b3736/b3310/b3309/b3185/b4147/b0240/b3165/b0422/b3636/b3316/b3230/b3733/b3936/b3318/b3319/b3320/b3317/b2606/b0170/b2231/b2296/b4039/b3315/b0680/b3311/b1876/b1610/b0526/b3342/b3314/b2697/b1770/b3065/b3312/b0382/b1284/b1817/b3306/b3891/b2805/b1866/b2707/b3133/b3313/b3983/b3307/b3590/b4393 |
| CC | GO:0005623 | cell | 0.00082 | 12 | b3510/b3240/b0585/b3932/b0220/b1716/b1717/b0188/b4320/b3343/b2218/b3398 | 82 | b0023/b3703/b3194/b4200/b1089/b4226/b4016/b1126/b2609/b3186/b4202/b1274/b4314/b1841/b3231/b3738/b2171/b3736/b1944/b3310/b0411/b0781/b3309/b3185/b1859/b4618/b3059/b4147/b0240/b3165/b0422/b3636/b3316/b3230/b3733/b4484/b3936/b3318/b1488/b0772/b3319/b0740/b3320/b3317/b2606/b0170/b3603/b2231/b2296/b4315/b4039/b3315/b0680/b3311/b1876/b1610/b0526/b3342/b3314/b2697/b1770/b3065/b0196/b3312/b0382/b1284/b1817/b1945/b3306/b3891/b0762/b2805/b1866/b2008/b2707/b3133/b3313/b3983/b1424/b3307/b3590/b4393 |
| CC | GO:0044464 | cell part | 0.00082 | 12 | b3510/b3240/b0585/b3932/b0220/b1716/b1717/b0188/b4320/b3343/b2218/b3398 | 82 | b0023/b3703/b3194/b4200/b1089/b4226/b4016/b1126/b2609/b3186/b4202/b1274/b4314/b1841/b3231/b3738/b2171/b3736/b1944/b3310/b0411/b0781/b3309/b3185/b1859/b4618/b3059/b4147/b0240/b3165/b0422/b3636/b3316/b3230/b3733/b4484/b3936/b3318/b1488/b0772/b3319/b0740/b3320/b3317/b2606/b0170/b3603/b2231/b2296/b4315/b4039/b3315/b0680/b3311/b1876/b1610/b0526/b3342/b3314/b2697/b1770/b3065/b0196/b3312/b0382/b1284/b1817/b1945/b3306/b3891/b0762/b2805/b1866/b2008/b2707/b3133/b3313/b3983/b1424/b3307/b3590/b4393 |
| CC | GO:0043234 | protein complex | 0.034092 | 3 | b0978/b3932/b0733 | 8 | b3194/b1126/b3737/b3738/b3736/b0781/b1859/b3733 |
| MF | GO:0003735 | structural constituent of ribosome | 4.26E-06 | 2 | b1716/b1717 | 31 | b0023/b3703/b4200/b1089/b2609/b4202/b3231/b3637/b3310/b3309/b3185/b3165/b3636/b3316/b3230/b3936/b3318/b3319/b3320/b3317/b2606/b3315/b3311/b3342/b3314/b3065/b3312/b3306/b3313/b3983/b3307 |
| MF | GO:0005198 | structural molecule activity | 2.29E-05 | 3 | b1923/b1716/b1717 | 31 | b0023/b3703/b4200/b1089/b2609/b4202/b3231/b3637/b3310/b3309/b3185/b3165/b3636/b3316/b3230/b3936/b3318/b3319/b3320/b3317/b2606/b3315/b3311/b3342/b3314/b3065/b3312/b3306/b3313/b3983/b3307 |
